# Tailoring a Global Iron Regulon to a Uropathogen

**DOI:** 10.1128/mBio.00351-20

**Published:** 2020-03-24

**Authors:** Rajdeep Banerjee, Erin Weisenhorn, Kevin J. Schwartz, Kevin S. Myers, Jeremy D. Glasner, Nicole T. Perna, Joshua J. Coon, Rodney A. Welch, Patricia J. Kiley

**Affiliations:** a Department of Biomolecular Chemistry, University of Wisconsin—Madison, Madison, Wisconsin, USA; b Department of Medical Microbiology and Immunology, University of Wisconsin—Madison, Madison, Wisconsin, USA; c Great Lakes Bioenergy Research Center, University of Wisconsin—Madison, Madison, Wisconsin, USA; d Center for Genomic Science Innovation, University of Wisconsin—Madison, Madison, Wisconsin, USA; National Cancer Institute

**Keywords:** Fur, RyhB, iron regulation, CFT073, UPEC, ppGpp, metabolic adaptation, Sigma S

## Abstract

Pathogenicity islands and plasmids bear genes for pathogenesis of various Escherichia coli pathotypes. Although there is a basic understanding of the contribution of these virulence factors to disease, less is known about variation in regulatory networks in determining disease phenotypes. Here, we dissected a regulatory network directed by the conserved iron homeostasis regulator, ferric uptake regulator (Fur), in uropathogenic E. coli (UPEC) strain CFT073. Comparing anaerobic genome-scale Fur DNA binding with Fur-dependent transcript expression and protein levels of the uropathogen to that of commensal E. coli K-12 strain MG1655 showed that the Fur regulon of the core genome is conserved but also includes genes within the pathogenicity/genetic islands. Unexpectedly, regulons indicative of amino acid limitation and the general stress response were also indirectly activated in the uropathogen *fur* mutant, suggesting that induction of the Fur regulon increases amino acid demand. Using RpoS levels as a proxy, addition of amino acids mitigated the stress. In addition, iron chelation increased RpoS to the same levels as in the *fur* mutant. The increased amino acid demand of the *fur* mutant or iron chelated cells was exacerbated by aerobic conditions, which could be partly explained by the O_2_-dependent synthesis of the siderophore aerobactin, encoded by an operon within a pathogenicity island. Taken together, these data suggest that in the iron-poor environment of the urinary tract, amino acid availability could play a role in the proliferation of this uropathogen, particularly if there is sufficient O_2_ to produce aerobactin.

## Abstract (precis)

Host iron restriction is a common mechanism for limiting the growth of pathogens. We compared the regulatory network controlled by Fur in uropathogenic E. coli (UPEC) to that of nonpathogenic E. coli K-12 to uncover strategies that pathogenic bacteria use to overcome iron limitation. Although iron homeostasis functions were regulated by Fur in the uropathogen as expected, a surprising finding was the activation of the stringent and general stress responses in the uropathogen *fur* mutant, which was rescued by amino acid addition. This coordinated global response could be important in controlling growth and survival under nutrient-limiting conditions and during transitions from the nutrient-rich environment of the lower gastrointestinal (GI) tract to the more restrictive environment of the urinary tract. The coupling of the response of iron limitation to increased demand for amino acids could be a critical attribute that sets UPEC apart from other E. coli pathotypes.

## INTRODUCTION

Uropathogenic Escherichia coli (UPEC) represents a pathotype that, in addition to growing as a commensal in the intestinal tract, has the ability to colonize the urinary tract and cause disease (1–3). E. coli pathotypes, such as UPEC, have genomic insertions (i.e., pathogenicity islands) that carry genes that encode virulence factors required for pathogenesis and these factors are absent in nonpathogenic strains (4–6). Although the role of virulence factors in UPEC pathogenesis has been well studied (7–9), less is known about the contribution of host signals and pathotype regulatory networks in differentiating UPEC from other E. coli strains. Understanding how these regulatory networks vary between UPEC and nonpathogenic E. coli isolates can provide a framework for understanding the contribution of regulatory adaptations to the robustness of the pathogenic phenotype.

The types of metabolic pathways that are deployed when UPEC strains colonize the dissimilar niches of the intestinal and urinary tracts are also poorly understood. The intestinal tract is thought to have sufficient energetically favorable carbon sources (e.g., sugars), alternative electron acceptors for respiration, and micronutrients (10). In contrast, urine has a variety of poorer carbon sources (e.g., amino acids, nucleobases, citrate), and low levels of some micronutrients, such as iron, suggesting that the urinary tract is a more nutritionally challenging environment (11–13). Recent studies have highlighted the importance of UPEC metabolic adaptations that enable growth in urine to establishing a urinary tract infection (14, 15). In addition, transcriptomic and proteomic profiling, as well as mutant fitness analysis, support the notion that UPEC regulates the expression of genes that presumably optimize growth in the urinary tract (16–21).

Host restriction of trace minerals, including iron, is referred to as nutritional immunity and is a well-established mechanism for limiting the growth of pathogenic bacteria (22, 23). The ability of UPEC to upregulate iron acquisition pathways to sequester available iron appears to play a key role in overcoming the nutritional immunity of the host (24). Iron acquisition functions, particularly iron chelating siderophores, are known virulence factors for UPEC (8, 25). Mutation of *tonB*, a gene responsible for siderophore-mediated iron uptake, severely attenuated UPEC infection in a murine model of infection (26). Some iron uptake functions were found to be upregulated in a UPEC mutant lacking the iron-responsive DNA binding protein Fur (27), indicating that this regulator likely globally represses expression of this class of genes in UPEC as it does in other bacteria (28–31). Despite this, the scope of the pathways directly regulated by Fur for any UPEC strain has not been established, nor is it known how strain variation would impact the transcriptional profile when this master regulator is disrupted in a uropathogenic strain compared to nonpathogenic E. coli.

Fur and its regulon have been extensively studied in the nonpathogen E. coli K-12 (30, 32–36). Under iron-replete conditions, iron-bound Fur increases DNA binding, acting as a transcriptional repressor of most target genes. Under iron-limiting conditions, loss of Fur-DNA binding leads to derepression of genes that promote iron acquisition, among others, as well as the small RNA RyhB, which acts posttranscriptionally. Expression of some iron acquisition functions is increased by RyhB, but its major role is promoting an iron-sparing response by downregulating the translation of proteins with iron cofactors, preserving iron for essential proteins (37, 38). Genes regulated by Fur and RyhB in E. coli K-12 have been identified using a variety of biochemical assays, mutational analyses, reporter assays, genome-wide chromatin immunoprecipitation (ChIP) followed by high-throughput sequencing (ChIP-seq) and RNA expression assays (31–33, 35, 37, 39, 40). Importantly, ChIP-seq analysis of Fur binding was critical in distinguishing genes regulated directly or indirectly by Fur, or posttranscriptionally by RyhB in genome-scale studies (32). A recent study revealed a role of RyhB in UPEC siderophore production (27). However, information is lacking on the scope of Fur and RyhB regulation in uropathogenic E. coli and how this regulation compares to E. coli K-12.

In this study, we analyzed the roles of Fur and RyhB in the UPEC strain CFT073 to gain insight into the strategies that uropathogenic strains deploy in the iron-limiting environment of the urinary tract. Using genome-wide ChIP-seq, high-throughput RNA sequencing (RNA-seq), and proteomics, we identified genes regulated by Fur and RyhB. By comparing the Fur and RyhB regulatory network in strain CFT073 to that of E. coli K-12 (32), we defined common targets with roles in iron uptake, homeostasis, and the iron-sparing response. We also identified genes within the UPEC pathogenicity islands regulated by Fur and RyhB. Unexpectedly, we found a large number of conserved genes that were indirectly regulated by Fur or RyhB in UPEC, which were members of stress regulons. Measurement of RpoS as a proxy for the stress signal showed that amino acid addition mitigated the stress response, suggesting a drain on amino acid resources in the uropathogen *fur* mutant. We propose that UPEC may be well suited to occupy the iron-limiting environment of the urinary tract because it can offset its greater need for amino acid resources during periods of iron limitation with uptake of amino acids from urine.

## RESULTS

### Profile of genes differentially expressed in a *fur* mutant of strain CFT073.

**FIG 1:**
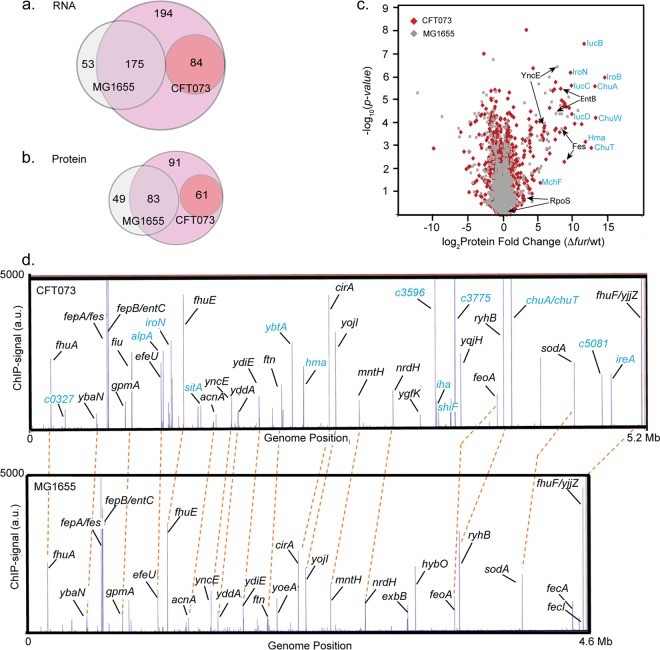
Genome-scale differences in Fur-dependent expression between UPEC strain CFT073 and commensal E. coli strain MG1655. E. coli strains were grown under anaerobic, iron-sufficient conditions. (a and b) Venn diagrams comparing the number of differentially expressed RNAs or proteins (>2-fold change in expression and *P* < 0.05) in the Δ*fur* mutant strains relative to parent strains CFT073 and MG1655 (32). The region of overlap indicates RNAs (a) or proteins (b) that are regulated by Fur in both CFT073 (pink circle) and MG1655 (gray circle). Fur-regulated CFT073 genes that have no orthologs in strain MG1655 are indicated by the smaller dark pink circle. (c) Volcano plot comparing Fur-regulated proteins from strains CFT073 and MG1655. The *x* axis indicates the log_2_ fold change in protein levels in the Δ*fur* mutant/wild-type (wt) strain of CFT073 (red) and MG1655 (gray). The *y* axis represents –log_10_ *P* values for individual proteins. Examples of CFT073-specific proteins (blue font) and orthologous proteins (black font) are indicated. (d) Comparison of genome-wide Fur binding from strains CFT073 and MG1655. The *x* axis indicates the genomic position of Fur ChIP-seq peaks from CFT073 (version NC_004431.1; top panel) or MG1655 (version U00096.2; bottom panel). The *y* axis indicates the normalized sequencing read count (in arbitrary units [a.u.]). Enrichment of Fur DNA binding is indicated by the height of the lines in each track. Examples of ChIP-seq peaks upstream of CFT073-specific genes are indicated in blue font, and conservation of ChIP-seq peaks upstream of orthologous genes (black font) is indicated by the orange dashed lines. A complete list of CFT073 ChIP-seq peaks is shown in Table S3 in the supplemental material.

To identify genes regulated by Fur, we compared RNA-seq profiles of the wild-type strain and the Δ*fur* mutant of UPEC strain CFT073. Anaerobic growth on morpholinepropanesulfonic acid (MOPS) minimal medium supplemented with glucose (glucose minimal medium) was used for this analysis because anaerobiosis maximized the number of genes regulated by Fur in E. coli K-12 (32) and because aerobic conditions severely impaired the growth of CFT073Δ*fur* in this medium (see Fig. S1 in the supplemental material). These results showed that 453 genes were differentially expressed at least twofold with a *P* value of 0.05 (see Tables S1 to S3 in the supplemental material), including 369 genes from the core genome and 84 genes from the pathogenicity islands. Comparison of these results to E. coli K-12 strain MG1655 (32) showed that 175 of the 369 orthologous genes were similarly regulated by Fur in the two strains (Fig. 1a). Many of the similarly regulated orthologs and pathogenicity island genes showed the expected signature of functions involved in iron homeostasis and acquisition—e.g., siderophore biosynthesis, heme, ferric, or ferrous iron uptake systems, iron storage proteins, the small RNA RyhB, and proteins predicted to be regulated by RyhB for iron sparing, etc. (Tables S1 and S2). However, an additional 194 orthologous genes were regulated by Fur in strain CFT073 but not in strain MG1655 (32), and these genes were enriched in stress response functions (Fig. 1a and Table S3). A direct proteomic comparison of proteins that were differentially expressed between MG1655 and CFT073 Δ*fur* mutants relative to their parent strains confirmed the pattern of Fur-regulated gene expression between the two strains; 83 orthologous proteins were similarly regulated by Fur in both strains, whereas 61 CFT073-specific proteins and 91 orthologous proteins were regulated by Fur only in CFT073 (Fig. 1b and c and Table S4). Taken together, these observations suggest that the loss of Fur in UPEC had a broader effect on gene expression than found with E. coli K-12.

10.1128/mBio.00351-20.1 FIG S1 Comparison of growth of strains CFT073 and MG1655 under aerobic conditions. Cell density was measured over time under aerobic conditions. (a) Comparison of CFT073 (red) and CFT073Δ*fur* (orange) grown in MOPS minimal glucose media under aerobic conditions. (b) Comparison of CFT073 grown in MOPS minimal media alone (red), with 100 μM each of all 20 amino acids (all AA; gold), with 1.0 mM DTPA iron chelator (orange); with 1.0 mM DTPA and 100 μM each of OAA-derived amino acids Asp, Lys, Thr, Ile, Met, and Asn (DTPA + OAA; blue), with 1.0 mM DTPA and 100 μM each of all 20 amino acids (DTPA + all AA; gray). (c) Comparison of MG1655 grown in MOPS minimal media alone (red), with 100 μM each of all 20 amino acids (all AA; gold), with 1.0 mM DTPA iron chelator (orange); with 1.0 mM DTPA and 100 μM each of OAA-derived amino acids Asp, Lys, Thr, Ile, Met, and Asn (DTPA + OAA; blue) with 1.0 mM DTPA and 100 μM each of all 20 amino acids (DTPA + all AA; gray). Download FIG S1, TIF file, 0.9 MB. Copyright © 2020 Banerjee et al. 2020 Banerjee et al. This content is distributed under the terms of the Creative Commons Attribution 4.0 International license.

10.1128/mBio.00351-20.5 TABLE S1 CFT073 Fur direct regulon. Data set of CFT073 operons that have a Fur ChIP-seq peak in the upstream regulatory region and showed a Fur-dependent change in RNA expression. Download Table S1, XLSX file, 0.02 MB. Copyright © 2020 Banerjee et al. 2020 Banerjee et al. This content is distributed under the terms of the Creative Commons Attribution 4.0 International license.

10.1128/mBio.00351-20.6 TABLE S2 Genes regulated by RyhB in both CFT073 and MG1655. Data set of CFT073 operons that have at least one gene regulated by RyhB in both CFT073 and MG1655 under the specified growth conditions. Download Table S2, PDF file, 0.1 MB. Copyright © 2020 Banerjee et al. 2020 Banerjee et al. This content is distributed under the terms of the Creative Commons Attribution 4.0 International license.

10.1128/mBio.00351-20.7 TABLE S3 CFT073 Fur indirect regulon. Data set of CFT073 operons that do not have a Fur ChIP-seq peak in the upstream regulatory region and a RyhB binding site at the 5’UTR but showed differential RNA expression under the specified growth conditions. Download Table S3, PDF file, 0.1 MB. Copyright © 2020 Banerjee et al. 2020 Banerjee et al. This content is distributed under the terms of the Creative Commons Attribution 4.0 International license.

10.1128/mBio.00351-20.8 TABLE S4 Proteomics data set of differentially expressed proteins comparing wt, *fur, ryhB*, and *fur ryhB* mutants of strains CFT073 and MG1655. Download Table S4, PDF file, 0.1 MB. Copyright © 2020 Banerjee et al. 2020 Banerjee et al. This content is distributed under the terms of the Creative Commons Attribution 4.0 International license.

### The direct Fur regulon of CFT073 includes genes from the core genome and pathogenicity islands.

**FIG 2:**
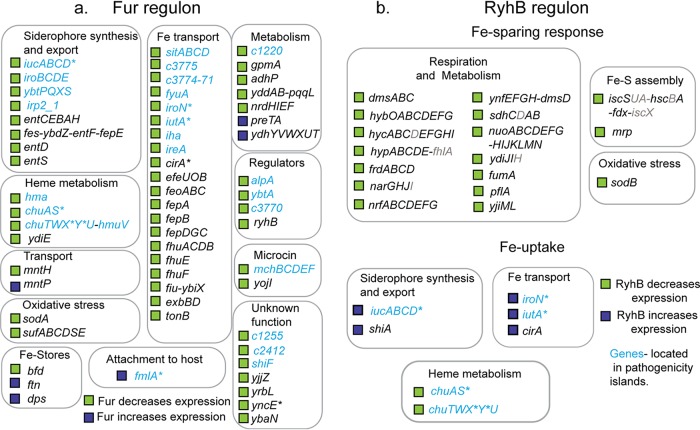
Role of Fur and RyhB in regulation of CFT073 genes differentially expressed in the *fur* mutant. (a) The CFT073 direct Fur regulon. CFT073 genes that had a Fur ChIP-seq peak in the 5′ upstream region and had >2-fold change (*P* < 0.05) in RNA-seq expression comparing the Δ*fur* strain to the wild-type strain were assigned to the direct regulon. (b) CFT073 genes predicted to be regulated by RyhB. Operons in which at least one gene (indicated in black type) showed >2-fold change (*P* < 0.05) in expression comparing the Δ*fur* strain to the wild-type strain and expression was at least partially reversed in Δ*fur*Δ*ryhB* strains indicated RyhB regulation; genes that showed a similar trend but did not meet the above-mentioned cutoff are marked in gray. Candidates for the iron-sparing response were operons decreased by RyhB, encoded iron-containing proteins, had predicted RyhB pairing sites, and regulation was conserved in MG1655 (32). Several iron uptake genes were regulated by both Fur and RyhB (indicated by an asterisk).

To determine which differentially expressed genes were directly regulated by Fur in strain CFT073, we used ChIP-seq to map Fur binding regions across the genome from cells cultured in the same anaerobic conditions used for the RNA-seq/proteomic experiments. Of the 349 Fur ChIP-seq peaks detected across the CFT073 genome, about half were present in intergenic regions characteristic of regulatory sites (Fig. 1d and Table S5). Genes were assigned to the direct Fur regulon if Fur bound within 300 bp upstream of a target gene in the ChIP-seq analysis and corresponding operons were differentially expressed in CFT073Δ*fur* from the RNA-seq analysis. By these criteria, we found that Fur directly regulated only 57 operons (119 genes) (Table S1 and Fig. 2a), despite the large number of Fur binding sites and differentially expressed genes in our analysis. Remarkably, none of the genes of the noted stress responses appeared to be directly regulated by Fur. Of the 57 operons regulated by Fur in CFT073, 35 belonged to the core genome, whereas 22 were located within the pathogenicity islands.

10.1128/mBio.00351-20.9 TABLE S5 CFT073 Fur ChIP-seq peaks. (A) CFT073 genes that have a Fur binding site as determined by ChIP-seq. (B) Fur binding motifs for ChIP-seq peaks for CFT073-specific genes. Download Table S5, PDF file, 0.2 MB. Copyright © 2020 Banerjee et al. 2020 Banerjee et al. This content is distributed under the terms of the Creative Commons Attribution 4.0 International license.

Fur-regulated genes of the core genome included iron homeostasis and acquisition functions—synthesis of the siderophore enterobactin (*entCEBAH*, *entD*, *entS*), siderophore-mediated ferric ion uptake (e.g., *cirA*, *fepA*, *fepB*, *fepDGC*, *fepE*, *exbBD*, *tonB*), ferrous ion transport (*feoABC*, *efeUOB*), iron storage (*dps*, *ftn*, *bfd*), Fe-S cluster biogenesis (*sufABCDE*), and the small RNA RyhB. In addition, the regulon included metabolic functions (*gpmA*, *nrdHIEF*, *adhP*, *ydhYVWXUT*, *yddAB-pqqL*), superoxide dismutase (*sodA*), ion transport (*mntH*), and several proteins of unknown function (*ybaN*, *yojI*, *yjjZ*, *yrbL*, and *yncE*) (Fig. 2a and Table S1). Comparing the CFT073 Fur regulon to that of strain MG1655 cultured under the same conditions (32) showed that 34 of the 35 operons located in the core genome were analogously regulated by Fur (Table S1). The Fur binding sites were also conserved between strains, indicating a highly conserved core regulon (Table S1). Minor differences between the direct Fur regulons of strains CFT073 and MG1655 could be explained in part by the absence of genes in CFT073 for the *fec* operon involved in ferric citrate transport, the sigma factor (*fecI*) governing this process, and the acid phosphatase regulator (*appY*). Expression of a predicted oxidoreductase (*ydhYVWXUT*) was regulated by Fur in CFT073, but this operon was below the limit of RNA detection in MG1655, although it had a ChIP-seq peak in the promoter region (32). Conversely, four genes regulated by Fur in MG1655—*map*, *gltA*, *recN*, and *amiA*—had no change in expression in CFT073, although a ChIP-seq peak was present in each promoter region (32).

The 22 operons directly repressed by Fur within the pathogenicity islands of strain CFT073 (Table S1 and Fig. 2a) are enriched in genes with iron acquisition functions, but these operons also encompass genes without a previously established connection to iron homeostasis. These genes include a predicted prophage transcription factor (*alpA*), the *mchBCDEF* operon that encodes the small antimicrobial peptide microcin H47 and predicted secretion machinery (Fig. S2), the F-pilin-like protein (*fmlA*), and genes of unknown function (*c2412*, *c1255*, and *shiF*). The known iron acquisition functions include a third ferrous iron uptake system (*sitABCD*), heme-mediated iron uptake and metabolism (*chuAS*, *chuTWXYU*, *hma*, *hmuV*), synthesis of two additional siderophores, aerobactin (*iucABCD*) and salmochelin (*iroBCDE*), and their respective receptors, *iutA* and *iroN*. Less well characterized siderophore receptors (*iha*, *ireA*, and *c3775*) and the Fit pathway of siderophore-mediated iron uptake (*c3771-c3774*) provide additional Fur-regulated pathways for ferric uptake (41). Fur also regulates *c1220* encoding an isozyme of 3-deoxy-7-phosphoheptulonate synthase that catalyzes the formation of shikimic acid, a precursor for enterobactin and salmochelin (19). A gene cluster encoding components of a siderophore biosynthetic pathway for yersiniabactin (*ybtPQXS*, *irp2*_1), its receptor (*fyuA*), and a transcription factor (*ybtA*) is also part of the direct Fur regulon, although this siderophore is not produced in CFT073 because several genes contain mutations (25, 42). In summary, our results demonstrate that Fur directly regulates iron uptake systems located in the pathogenicity islands of CFT073, enabling scavenging of diverse iron sources when iron is scarce.

10.1128/mBio.00351-20.2 FIG S2 Regulation of microcin operon by Fur. (a) Genome Browser view of Fur ChIP-seq peak (blue) in the upstream region of *mchB* and aligned RNA-seq reads of *mchB, mchC, mchD, mchE*, and *mchF* from wild-type (undetectable), Δ*fur* (purple), and Δ*fur*Δ*ryhB* (green) strains. Also shown are the averaged RNA expression levels from the same RNA-seq data (b) and protein abundance (c) from proteomic data from the same strains. Peptides that map back to MchB were not detected in our proteomic data set. Download FIG S2, TIF file, 0.7 MB. Copyright © 2020 Banerjee et al. 2020 Banerjee et al. This content is distributed under the terms of the Creative Commons Attribution 4.0 International license.

### RyhB positively controls the expression of siderophore-mediated iron uptake in strain CFT073.

Expression of the small RNA posttranscriptional regulator RyhB was upregulated in the CFT073 *fur* mutant, confirming that it is a Fur-repressed target (Table S1). Accordingly, deletion of *ryhB* in a Fur^+^ strain is not expected to cause a change in gene expression compared to the parent strain, since RyhB was not expressed. Thus, to identify genes that are regulated by RyhB, we compared genes differentially expressed in CFT073Δ*fur*, when RyhB is expressed, to CFT073Δ*fur*Δ*ryhB*, lacking RyhB. For a control, we analyzed the effect of deleting just *ryhB* in CFT073, which as expected, did not lead to significant changes in gene expression (Tables S1 to S3). This analysis also revealed the relative contribution of each regulator, RyhB or Fur, to gene expression.

**FIG 3:**
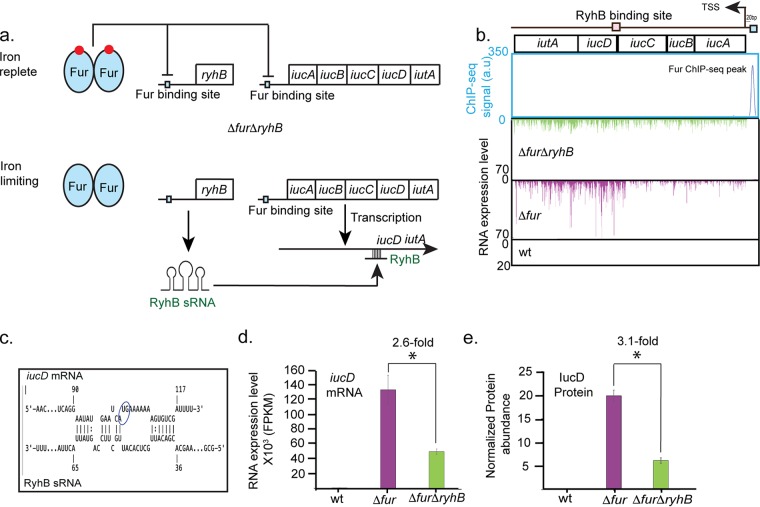
Both Fur and RyhB regulate aerobactin production. (a) Model depicting the independent roles of Fur in transcriptional repression of the *iucABCD-iutA* operon under iron-replete conditions and of RyhB in increasing RNA levels of the aerobactin biosynthesis gene *iucD* and its receptor *iutA* under iron-limiting conditions when Fur is inactivated. sRNA, small RNA. (b) Fur ChIP-seq peak and RNA-seq reads from the wild-type strain (not observed) and Δ*fur* (purple) and Δ*fur* Δ*ryhB* (green) mutant strains were aligned to the CFT073 genome and visualized with the MochiView genome browser depicting the region of aerobactin biosynthetic operon, which is transcribed counterclockwise. TSS, transcription start site. (c) Computational prediction of a RyhB pairing site overlapping the start codon (circled) of *iucD* aligned with a segment of RyhB. The binding energy was predicted to be −10.9 kcal/mol. (d) Averaged *iucD* RNA-seq expression from the wild-type strain (undetectable) and Δ*fur* (purple) and Δ*fur*Δ*ryhB* (green) mutant strains are taken from Table S1. (e) IucD protein abundance in the wild-type, Δ*fur*, and Δ*fur*Δ*ryhB* strains were taken from Table S4. An asterisk indicates *P* of <0.05 as determined from a paired Student’s *t* test. Similar results were observed for *iutA* (Tables S1 and S4). FPKM, fragments per kilobase per million.

Both Fur and RyhB were found to regulate the expression of several siderophore biosynthetic or receptor genes (Table S1 and Fig. 2). Within the pathogenicity islands, the entire *iucABCD-iutA* operon, encoding aerobactin synthesis enzymes and its receptor, was derepressed in the *fur* mutant, whereas only *iucD* (encoding the first enzyme of the pathway) and *iutA* (the receptor) were additionally upregulated by RyhB in the *fur* mutant (Fig. 3a to e). A predicted RyhB base-pairing site was found overlapping the *iucD* translational start site, suggesting a direct effect of the small RNA (Fig. 3c). The salmochelin receptor encoded by *iroN* was also positively regulated by RyhB and repressed by Fur, and the RyhB pairing site previously found for Salmonella enterica *iroN* (43) was conserved in strain CFT073, suggesting a direct effect. In comparison, expression of *chuS* and *chuXY*, genes involved in the uptake of iron from heme, was decreased by RyhB even though their expression is derepressed in a Fur mutant, suggesting that not all pathways of iron acquisition are regulated similarly by RyhB (Table S1 and Fig. 2). Within the core genome, *cirA*, the receptor for 2,3-dihydroxybenzoylserine breakdown products of enterobactin, was found to be regulated by both RyhB and Fur, whereas *shiA*, the shikimate:H^+^ symporter that transports a precursor of enterobactin, shikimate, was regulated only by RyhB (Tables S1 and S2 and Fig. 2), as found in E. coli K-12 (44). Taken together, these data suggest that RyhB may have a previously unrecognized role in directing a hierarchy of iron source utilization in CFT073; RyhB amplifies the positive effect of Fur inactivation by increasing expression of siderophore-mediated iron uptake but attenuates the positive effect of Fur inactivation with respect to heme uptake.

### RyhB equips CFT073 with a conserved iron-sparing response.

To address the function of RyhB in the iron-sparing response established in E. coli K-12, we found 17 operons that encode predicted iron-containing proteins for which at least one gene showed decreased expression in the presence of RyhB when comparing RNA-seq data from the CFT073Δ*fur*Δ*ryhB* strain to the Δ*fur* mutant (Fig. 2b and Table S2). These candidate RyhB-regulated CFT073 mRNAs were also found to be decreased by RyhB in E. coli K-12 (32) and encode the Fe-superoxide dismutase (SodB), the Isc Fe-S cluster biogenesis pathway, and the respiratory enzymes, NADH dehydrogenase I, succinate dehydrogenase, fumarate reductase, dimethyl sulfoxide reductase, hydrogenase 2, nitrate reductase, nitrite reductase, and other iron-containing proteins. Thus, comparable cellular functions are downregulated to preserve iron pools in strain CFT073 under anaerobic iron-limiting conditions as in *E. coli* K-12. Seven other operons showed a RyhB-dependent increase in RNA levels, and four showed a RyhB-dependent decrease in both CFT073 and MG1655, suggesting a conserved response by RyhB, although the connection to iron metabolism was unclear, and their direct control by RyhB has not been demonstrated.

### The absence of Fur indirectly impacts stress pathways in strain CFT073.

**FIG 4:**
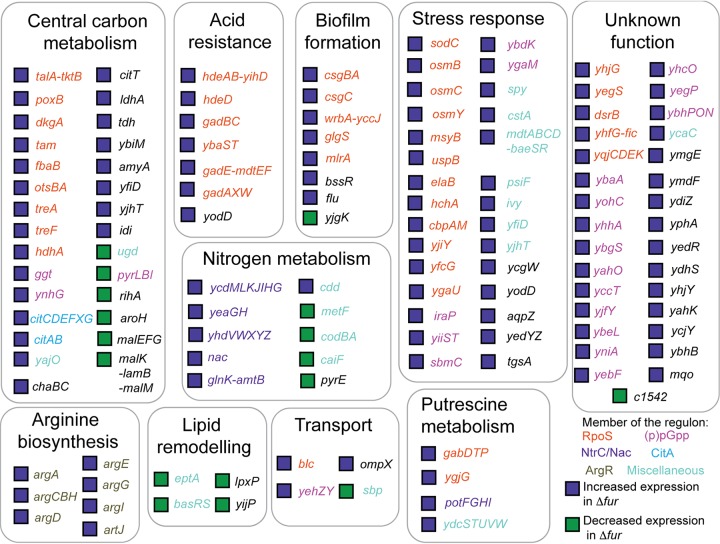
CFT073 genes indirectly regulated by Fur and RyhB include members of several stress regulons. CFT073 genes that lacked a Fur ChIP-seq peak and had >2-fold change (*P* < 0.05) in expression in comparing the Δ*fur* strain to the wild-type strain but lacked an analogous change in expression in strain MG1655 are shown. In addition, a subset of these genes also showed regulation by RyhB but a putative RyhB binding site was lacking in the 5′ upstream region (Table S3). Operons that show >2-fold upregulation in Δ*fur* strain (purple square) and those that show >2-fold downregulation in Δ*fur* strain (green square) were clustered based on their functions and are colored differently according to which regulon they are part of.

Further analysis of the 194 orthologous genes that were regulated by Fur and/or RyhB in strain CFT073 but not in strain MG1655 showed an absence of Fur ChIP-seq peaks or predicted RyhB base-pairing sites in the 5′ untranslated region (5′UTR) (Fig. 4 and Table S3), suggesting indirect regulation by Fur or RyhB. Clustering of these indirectly regulated genes using the regulatory networks of E. coli K-12 from the EcoCyc database (45) showed that 155 genes could be assigned to regulons that included the general stress response (controlled by RpoS) (46), the stringent response [controlled by (p)ppGpp/RNA polymerase] (47), the nitrogen limitation response (controlled by NtrC) (48–50), or arginine biosynthetic operons (e.g., controlled by ArgR), suggesting a connection between the indirect regulon of Fur and stress response pathways (Fig. 4 and Table S3). The remaining 39 genes encode functions that are not well described, or information was unavailable about their mode of regulation in the EcoCyc database.

A comparison of the regulatory molecules that activated these stress modules suggests that the *fur* mutant had an unexpected defect in amino acid pools. For example, activation of NtrC occurs in response to depletion of glutamine and increase in α-ketoglutarate (50). The ArgR regulon is induced when arginine levels are insufficient. Since amino acid biosynthetic operons were derepressed in the parent strain because of growth on minimal medium, the further derepression of the ArgR and NtrC regulon suggested an exaggerated response in the *fur* mutant. The stringent response is activated by increased (p)ppGpp in response to amino acid limitation (51–53). Furthermore, levels of RpoS, the sigma factor controlling the general stress response, increase in response to (p)ppGpp via the upregulation of and stabilization by the antiadaptor IraP (54, 55), providing a link between the stringent response and the general stress response.

**FIG 5:**
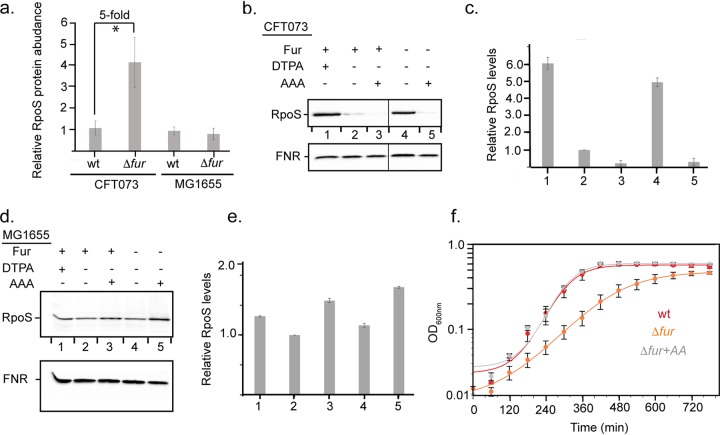
Amino acid addition to strain CFT073 mitigates stress induced by Fur inactivation. (a) RpoS protein abundance in wild-type and Δ*fur* strains of CFT073 and MG1655 as measured by proteomics (Table S4) and analyzed by a paired Student’s *t* test. (b) RpoS and FNR Western blot analysis of strain CFT073 (lanes 1 to 3) and Δ*fur* mutant (lanes 4 and 5) grown anaerobically in MOPS minimal medium supplemented with glucose and 1.0 mM DTPA (iron chelator; lane 1), no addition (lanes 2 and 5), 100 μM each of all 20 amino acids (AAA) (lanes 3 and 5). (c) Quantification of RpoS levels from Western blots in panel b. RpoS levels were normalized to FNR levels as an internal control and then scaled the wild-type RpoS value from lane 2 to one. (d) RpoS and FNR Western blot analysis of MG1655 (lanes 1 to 3) and MG1655Δ*fur* (lanes 4 and 5). Lane 1, 1.0 mM DTPA; lanes 2 and 4, no addition; lane 3 and 5, 100 μM each of all 20 amino acids. (e) RpoS levels were quantified using the same method as for strain CFT073. (f) Amino acids can rescue the growth defect of the CFT073 Δ*fur* mutant. Cell density was measured over time for the wild-type strain (red), Δ*fur* mutant without (orange) or with all 20 amino acids (+AA; gray) under anaerobic growth conditions in MOPS minimal medium supplemented with glucose. The data represent mean values for three biological replicates, and the error bars represent the standard deviations.

To test whether amino acid limitation played a role in the induction of the stress responses observed in the CFT073Δ*fur* mutant, we asked whether amino acid addition mitigated the stress. We reasoned that measuring RpoS (sigma S) levels would be a reasonable proxy for determining the stress levels in the *fur* mutant, since our proteomic data showed that RpoS levels increased fourfold in CFT073Δ*fur* (Fig. 5a). To assay RpoS in these experiments, we used quantitative Western blotting, normalizing RpoS to fumarate and nitrate reductase (FNR), a protein that should not change its levels under these conditions (32) (Fig. S3). As expected from the proteomic data, deletion of *fur* resulted in an increase in RpoS levels (ca. fivefold) in strain CFT073 but had no effect in strain MG1655 when cells grown anaerobically in glucose minimal medium were compared (Fig. 5b to e). Further, supplementing cultures of CFT073Δ*fur* with all 20 amino acids restored RpoS to wild-type levels. In contrast, addition of amino acids to MG1655Δ*fur* had only a minor effect on RpoS levels (Fig. 5b to e). We also found that the increase in RpoS levels in CFT073Δ*fur* was phenocopied by growth of CFT073 with an iron chelator, diethylenetriamine pentaacetic acid (DTPA) (Fig. 5b and c); in the presence of the chelator, the Fur regulon should be derepressed as in the *fur* mutant, but iron uptake will be inhibited, showing that this stress response was caused by induction of the Fur regulon and not a result of perturbation of intracellular iron pools. We also compared the growth characteristics of CFT073 with or without Fur. CFT073Δ*fur* was slightly impaired for growth in glucose minimal medium under anaerobic conditions, compared to the wild-type strain, but wild-type growth was restored to CFT073Δ*fur* by adding amino acids to the growth medium (Fig. 5f). Taken together, these results suggest that amino acid pools are perturbed by Fur inactivation in CFT073 but not MG1655, resulting in less resources available for protein synthesis, etc., and accordingly, the induction of multiple stress responses in the uropathogen.

10.1128/mBio.00351-20.3 FIG S3 Establishing the linear range of Western blot analysis for RpoS and FNR. (a) Samples of 250 μl (0.5×), 500 μl (1×), and 1,000 μl (2×) of wild-type and Δ*fur* CFT073 strains grown anaerobically in MOPS minimal medium to an OD of 0.2 were collected by centrifugation. Cells were lysed, and lysates were electrophoresed and then blotted with antibody specific for FNR (internal control) and RpoS. (Right) Plot of the imaged signal for each volume of cells. (b) Same as panel a but conducted with wild-type and Δ*fur* MG1655 strains. (c) Western blot analysis establishes the absence of Fur in CFT073Δ*fur*. Four biological replicates of wild-type and Δ*fur* CFT073 strains were grown aerobically in LB. Equivalent amounts of cell lysate were electrophoresed on 16% resolving SDS-PAGE gel, transferred to nitrocellulose membrane, and incubated with antibody specific for Fur. Download FIG S3, TIF file, 1.6 MB. Copyright © 2020 Banerjee et al. 2020 Banerjee et al. This content is distributed under the terms of the Creative Commons Attribution 4.0 International license.

**FIG 6:**
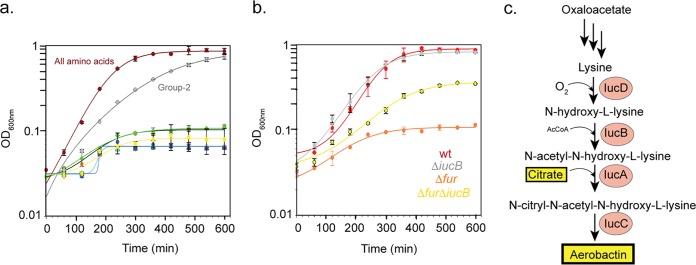
Elimination of Fur imparts a growth defect for strain CFT073 under aerobic conditions. (a) Growth of the Δ*fur* mutant can be largely rescued by adding amino acids derived from oxaloacetate, a metabolic precursor to aerobactin. Cell density was measured for the CFT073Δ*fur* strain grown under aerobic conditions in MOPS minimal medium supplemented with glucose and either all 20 amino acids (dark red) or six different mixtures of amino acids, which were grouped based upon common metabolic precursors. Relevant amino acids were added at a final medium concentration of 100 μM. Group 1 amino acids (Gln, Glu, Pro, and Arg) are derived from α-ketoglutarate (green). Group 2 amino acids (Asp, Lys, Thr, Ile, Met, and Asn) are derived from oxaloacetate (gray). Group 3 (Val, Ala, and Leu) are derived from pyruvate (orange). Group 4 amino acids (Ser, Cys, and Gly) are derived from 3-phosphoglycerate (cyan). Group 5 (His) is derived from phosphoribosyl pyrophosphate (blue). Group 6 amino acids (Trp, Tyr, and Phe) are derived from erythrose 4-phosphate and phosphoenolpyruvate (yellow). (b) Disrupting aerobactin biosynthesis partially recovers growth of CFT073 Δ*fur* under aerobic conditions. Cell density was measured over time for the wild-type strain (red) and Δ*fur* (orange), Δ*iucB* (gray), and Δ*fur* Δ*iucB* (yellow) mutant strains under aerobic conditions in MOPS minimal medium supplemented with glucose. The data represent the mean values for three biological replicates, and the error bars represent the standard deviation. (c) Aerobactin biosynthesis. AcCoA, acetyl coenzyme A.

We also tested whether amino acid addition could rescue the severe growth defect of CFT073Δ*fur* observed under aerobic conditions (Fig. S1). Addition of all 20 amino acids was sufficient to restore growth (Fig. 6a and b). Unlike strain MG1655, which lacked this severe aerobic growth phenotype (32), strain CFT073 produces large amounts of the siderophore aerobactin under aerobic conditions (27). Aerobactin synthesis should be limited to aerobic conditions, because the first step, catalyzed by IucD, requires O_2_ (56). Since the first step also requires lysine, we considered whether the severe growth defect of the CFT073 *fur* mutant under aerobic conditions was due to the diversion of lysine to aerobactin (Fig. 6c), shifting metabolic resources away from protein synthesis or peptidoglycan biosynthesis, which requires lysine or its precursors, and exacerbating the growth defect observed under aerobic conditions. To test this possibility, we subdivided the amino acids into pools based on biosynthetic precursors and showed that addition of just oxaloacetate (OAA)-derived amino acids (Asp, Lys, Thr, Ile, Met, and Asn) was sufficient to restore CFT073Δ*fur* to the same final cell density attained when all 20 amino acids were added, although the growth rate was twofold lower (Fig. 6a). We then tested whether inactivation of aerobactin synthesis could rescue growth by insertion of a polar mutation in *iucB* (26) in wild-type and Δ*fur* CFT073 backgrounds. CFT073Δ*fur*Δ*iucB* showed partial recovery of growth, achieving 3 times more biomass compared to the Δ*fur* strain (Fig. 6b). While the recovery was not complete, this result suggests that diversion of amino acid pools by aerobactin biosynthesis can be one driver for iron-dependent changes to UPEC physiology in the presence of O_2_.

We also found that adding an iron chelator largely mimics the severe growth defect of the CFT073 *fur* mutant under aerobic conditions. Growth can be partially rescued by OAA-derived amino acids and is fully rescued by adding all amino acids, as we observed with the CFT073 *fur* mutant (Fig. S1). In contrast, the effect of adding the external iron chelator to strain MG1655 has a less dramatic effect, consistent with previous results with the *fur* mutant (32).These data are most consistent with the growth defects of strain CFT073 resulting from both amino acid and iron limitation, while the growth defects of strain MG1655 likely reflect mainly iron limitation.

## DISCUSSION

In this study, we investigated the global role of the iron-dependent transcription factor Fur in the uropathogenic E. coli strain CFT073. Our results expand the number of direct and indirect targets regulated by Fur in strain CFT073, providing a comprehensive view of the global changes in gene expression that can occur when UPEC encounters an iron-limiting environment. Our results reaffirm that UPEC is armed with multiple pathways to acquire iron from its environment, using Fur to regulate their transcription and RyhB to further modulate levels of some components. Comparison of the Fur targets in this uropathogenic strain to that of commensal E. coli strain MG1655 (32) showed that many direct Fur regulon members are conserved and comprise a core Fur regulon in E. coli. However, CFT073 lacking Fur also had some expected phenotypes compared to strain MG1655. The induction of the stringent and general stress responses in the *fur* mutant, which was mitigated by amino acid supplementation, suggested that expression of the Fur regulon in CFT073 caused a drain on amino acid resources. This unexpected impact of iron limitation on amino acid resources may point to a novel mechanism for controlling proliferation of the uropathogen in the iron-limiting environment of the urinary tract.

Iron acquisition functions play a critical role in UPEC pathogenesis because the extracellular environment in the urinary tract is limited in iron availability (25). Indeed, many of the genes encoding components of the ferrous iron or heme uptake systems, components of the three siderophore biosynthetic pathways, various outer membrane siderophore receptors, and the TonB-ExbD system are upregulated when UPEC strains are grown in urine or in mouse models of urinary tract infections (18, 21, 57–59) (see Table S6 in the supplemental material). While in some cases the individual role of Fur or RyhB in controlling gene expression had been demonstrated or inferred (27, 43, 60), our global approaches of ChIP-seq, RNA-seq, and proteomics, allowed us to confirm predictions, distinguish between positive or negative impacts of Fur and RyhB, and identify new roles for these iron-responsive regulators on a genome-wide scale. Additional direct targets for Fur regulation within known pathogenicity islands were identified, such as a member of the major facilitator transport superfamily (ShiF), microcin H47 maturation enzymes (MchBCDEF), two predicted TonB dependent iron-siderophore receptors (Iha and IreA), a second system for ferric siderophore uptake referred to as the Fit pathway (c3771-3775) (41), an alternate isozyme (c1220) for synthesis of shikimate, an intermediate of aromatic amino acids, enterobactin and salmochelin synthesis. The latter isozyme is upregulated during a urinary tract infection (18) and promotes fitness during CFT073 systemic infection in mice (19). Thus, overall, Fur controls more operons that promote iron uptake in CFT073 than in MG1655.

10.1128/mBio.00351-20.10 TABLE S6 Genes differentially expressed in the CFT073 *fur* mutant compared to those grown in urine or obtained from a urinary tract infection. Download Table S6, PDF file, 0.1 MB. Copyright © 2020 Banerjee et al. 2020 Banerjee et al. This content is distributed under the terms of the Creative Commons Attribution 4.0 International license.

We found that RyhB along with Fur controls expression of the receptor for salmochelin (IroN) and aerobactin (IutA), and the enzyme catalyzing the first step in aerobactin synthesis (IucD), extending previous studies of RyhB regulation of siderophores in strain CFT073 (27). Expression of the entire *iucABCD-iutA* operon was found to be repressed by Fur, whereas only the last two genes were upregulated by RyhB, suggesting a need for higher levels of these products. A RyhB pairing site was predicted overlapping the Shine-Dalgarno sequence, but additional studies are needed to define the molecular mechanism of RyhB-mediated upregulation of *iucD* and *iutA* expression. In comparison, our data suggest that RyhB had a small negative effect on heme uptake systems in strain CFT073. Taken together, the use of posttranscriptional mechanisms to further regulate expression of iron acquisition functions could provide fine-tuning to the types of iron sources available to CFT073 in different host niches.

**FIG 7:**
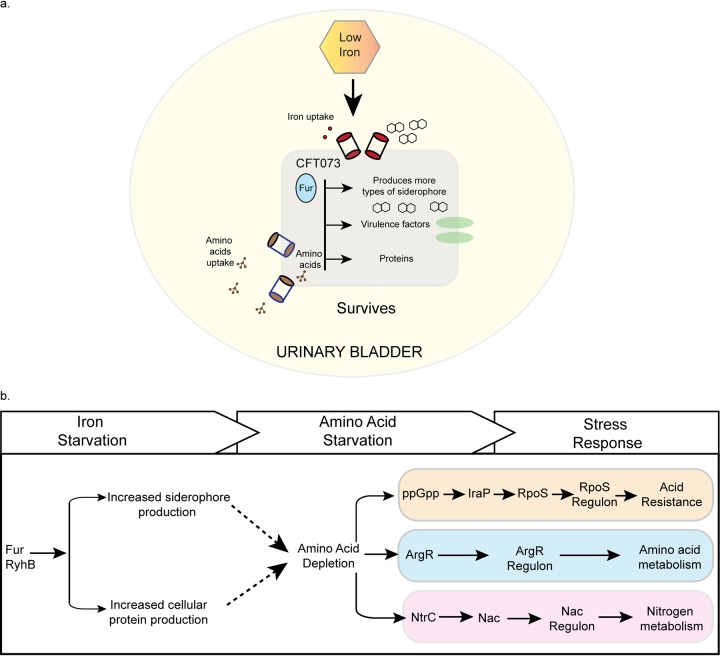
Induction of the Fur regulon indirectly induces stress responses. (a) Cellular overview of model showing how a low-iron environment of the urinary tract leads to induction of the Fur regulon, virulence factors (e.g., siderophores), and cellular proteins that give rise to an increased amino acid demand. Uropathogenic E. coli may offset its greater need for amino acid resources by transporting amino acids from the urinary tract. (b) A model describing our findings of the direct and indirect effects of Fur. Expression of the Fur regulon results in amino acid limitation, which in turn leads to induction of stress responses. The previously known genetic programs/regulons are shown in the right portion with the associated cause and effects.

We also observed unexpected changes in stress regulatory networks in the absence of Fur in strain CFT073. Activation of the stringent response in CFT073Δ*fur* was indicated by the differential expression of genes known to be regulated by ppGpp, the key regulator of this response (51, 52). When nutrients are limiting, (p)ppGpp increases, and changes in gene expression shift remaining resources away from growth to survival of bacteria until nutrient availability improves (51–53, 61). The observation that a stress indicator (RpoS) was returned to normal levels by the addition of amino acids and that two additional regulons (ArgR and NtrC), which respond to low amino acids, were also upregulated in CFT073Δ*fur* supports the notion that the stringent response was activated due to limiting amino acids. Induction of the stringent response was not observed in E. coli K-12 lacking Fur when comparing similar growth conditions (32). Yet, a previous study of E. coli K-12 showed that (p)ppGpp was induced under iron-limiting conditions, but this response was Fur independent, consistent with a different limiting nutrient input (e.g., iron) to increased ppGpp (62). Iron limitation also results in (p)ppGpp accumulation in the firmicute Enterococcus faecalis (63) and Bacillus subtilis (64), suggesting a similar mechanism to E. coli K-12. Thus, the amino acid limitation induced in CFT073Δ*fur* may reflect a uropathogen-specific adaptation for the urinary tract. We postulate that amino acid demand could be increased in the uropathogen because of production of abundant siderophores (27), derived from amino acids, and because of an increased protein synthesis burden associated with the 10% larger genome of strain CFT073, and the synthesis of Fur regulon proteins (Fig. 7). In support of an increased amino acid demand imposed by the expression of the Fur regulon, we found that the severe growth defect of the *fur* mutant under aerobic conditions could be relieved by supplementing the growth medium with all 20 amino acids. Siderophores appear to contribute to the burden, since eliminating the production of aerobactin or adding amino acids that feed into the pathway of aerobactin synthesis could partially relieve the severe growth defect.

The increase in ppGpp could also explain the upregulation of the RpoS regulon in the CFT073Δ*fur* mutant (Fig. S4). Previous studies showed that a *rpoS* mutant of strain CFT073 was outcompeted by the wild-type strain in a murine model of urinary tract infection, suggesting that the coordinated regulation and expression of the RpoS regulon, including genes involved in oxidative and acid stress resistance, may be important to UPEC virulence (65). In E. coli K-12, RpoS levels are controlled by antiadaptor proteins (Ira) that prevent RssB from promoting ClpXP-mediated degradation of RpoS (54, 66). The finding that expression of IraP is stimulated by (p)ppGpp (54, 55) and that its expression increased in CFT073 lacking Fur provides a link between activation of the stringent response with the coordinated expression of the RpoS regulon. In addition, the levels of the CFT073-specific RpoS antiadaptor IraL (the closest E. coli K-12 ortholog is IraM) were also increased in CFT073 lacking Fur. Thus, the increase in both IraL and IraP antiadaptor proteins likely explains the severalfold increase in RpoS protein levels in CFT073 lacking Fur. IraL has been shown to promote higher levels of RpoS during exponential growth of strain CFT073 than found in E. coli K-12 (67), suggesting a difference in the wiring and dynamic range for expression of the RpoS regulon between CFT073 and E. coli K-12. Perhaps regulation of RpoS levels in CFT073 is tuned in part to ensure expression of the RpoS regulon and accordingly, survival during periods of amino acid limitation in the urinary tract.

10.1128/mBio.00351-20.4 FIG S4 RpoS level are elevated in CFT073 Δ*fur* strain posttranscriptionally. (a) Model describing the connection of inactivation of Fur to increased stress response by upregulating the stability of RpoS. (b) The RNA-seq reads from the wild-type strain (orange) and Δ*fur* mutant (purple) were aligned to the CFT073 genome and visualized on the MochiView genome browser encompassing the RpoS gene. Download FIG S4, TIF file, 0.6 MB. Copyright © 2020 Banerjee et al. 2020 Banerjee et al. This content is distributed under the terms of the Creative Commons Attribution 4.0 International license.

The gene expression data also indicated that the cell surface may be modified in the CFT073 *fur* mutant. Previous studies have shown that UPEC strains have a repertoire of fimbriae, 10 chaperone-usher family and 2 putative type IV pili that are temporally expressed during infection (6, 58, 68, 69). Although we did not observe significant changes in expression of the type 1 pilin operon or the *fimB* and *fimE* recombinases that regulated *fimS* inversion in the *fur* mutant, we did observe negative effects of the mutant on expression of the two *pap* operons in strain CFT073 as well as one of the type 1-like fimbrial genes, C1936 (*fmlA*), suggesting that Fur enhances their expression under iron-sufficient conditions. In contrast, expression of the transcription regulator, *csgD* (activated by ppGpp and RpoS), and its downstream target genes *csgBAC*, are upregulated in the CFT073 *fur* mutant. CsgBAC encode curli, a nonfimbrial adhesin that plays a key role during biofilm formation in the urinary bladder in the initial stages of urinary tract infections (UTI) (69). Overall, this provides further evidence of the complex overlapping control of UPEC adhesins.

In summary, by taking a genome-wide approach, we found expected and unexpected changes in global transcription networks in strain CFT073 lacking Fur, indicative of iron limitation and metabolic stress. We postulate that direct and indirect effects of Fur on gene expression impart a metabolic burden on amino acid pools that is related to increased Fur regulon expression and activation of metabolic pathways (e.g., siderophores) in this uropathogen. The ability of UPEC to transport external amino acids from the urinary tract may provide a competitive advantage for the uropathogen by allowing it to overcome this metabolic burden and proliferate in nutritionally challenging and iron-limiting urine. Given the role of RyhB and iron acquisition functions in the virulence of this uropathogenic strain, the pathways found to be indirectly regulated by Fur and RyhB in strain CFT073 but not in the commensal MG1655 strain define previously underappreciated functions (stress responses, amino acid biosynthesis, cell surface assembly) beyond iron acquisition that may be relevant to pathogenesis. Furthermore, our findings also suggest that dilution of amino acid levels in the urinary tract might be an unexplored mechanism to control growth of this uropathogen.

## MATERIALS AND METHODS

### Strain construction.

The sequences of the DNA primers used in strain construction are available upon request. E. coli CFT073 (WAM4505) harboring λ-Red recombination machinery (WAM4507) was used to make gene deletions. The primers for deleting *fur* in frame were designed such that the entire open reading frame (ORF) was replaced with a chloramphenicol resistance gene, whereas for deleting RyhB, the primer was designed such that the entire gene (starting from +1 to 90 bp) was replaced with a kanamycin cassette. The deletion/insertion alleles were transduced into CFT073 wild-type strain WAM4505 using phage EB49 (70). Finally, the antibiotic resistance cassettes were removed by transforming strains with pCP20 that encodes FLP recombinase, thereby generating CFT073Δ*fur* (WAM5491) and Δ*ryhB* (WAM5497) (71). To generate the Δ*fur* Δ*ryhB* mutant strain (WAM5499), *ryhB*, replaced with a kanamycin cassette, was transduced into strain WAM5491, followed by removal of antibiotic cassette with pCP20. All mutations were confirmed by DNA sequencing, and deletion of *fur* was further confirmed by Western blot analysis (see Fig. S3c in the supplemental material). To create a mutant strain defective in aerobactin biosynthesis, an allele of *iucB* that was replaced by a chloramphenicol cassette (26) was transduced in CFT073 wild-type (WAM4505) and *fur* mutant (WAM5491) using phage EB49.

### Growth of cultures.

Strains were grown in morpholinepropanesulfonic acid (MOPS) minimal medium (pH 7.4) supplemented with 0.2% glucose, which contains 10.0 μM FeSO_4_, an amount considered sufficient for iron (32). In some experiments, 0.2% Casamino Acids (final concentration) was added to the growth medium. Anaerobic growth of cultures was achieved by sparging cells with a gas mix containing 95% N_2_ and 5% CO_2_, whereas aerobic growth cultures were sparged with 70% N_2_, 25% O_2_, and 5% CO_2_ as described previously (32). Optical density at 600 nm (OD_600_) was measured in a Perkin Elmer Lambda 25 UV-visible (UV-Vis) spectrophotometer. For experiments where the impact of defined amino acid mixes was examined, cultures were grown in aerated test tubes or capped tubes in standing conditions for anaerobic experiments.

### Chromatin immunoprecipitation followed by high-throughput sequencing.

Chromatin immunoprecipitation (ChIP) followed by high-throughput sequencing (ChIP-seq) was conducted by the method of Beauchene et al. (32). Briefly, three biological replicates of wild-type CFT073 (WAM4505) and one biological replicate of the Δ*fur* mutant strain (WAM5491) were grown anaerobically in MOPS minimal medium supplemented with 0.2% glucose sparged with a gas mix of 95% N_2_ and 5% C O_2_ until an OD_600_ of 0.25 was reached. Cellular DNA was cross-linked to proteins by the addition of 1% formaldehyde. Cells were harvested at 7,500 rpm for 10 min at 4°C, which was followed by sonication using a water bath sonicator (Misonix) to shear the DNA to a fragment size of 150 to 400 bp to facilitate library production. The cell lysate was incubated with antibody specific to Fur that was affinity purified over His_6_-Fur-bound HiTrap *N*-hydroxysuccinimide (NHS)-activated high-performance (HP) column (GE Healthcare) (72). DNA was reverse cross-linked and eluted from the DNA-protein complex using 50 mM Tris (pH 8.0), 10 mM EDTA, and 1% sodium dodecyl sulfate (SDS). Ten nanograms of purified input or immunoprecipitated (IP) DNA was used to construct DNA libraries as described in the Illumina TruSeq ChIP preparation kit (catalog no. IP-202-1012), except the products of ligation reactions were purified using 2% size select agarose gel (Invitrogen). After library construction and amplification, quantity and quality were validated using a Qubit 2.0 fluorometer (Invitrogen, Carlsbad, CA) and Agilent 2100 bioanalyzer before submitting them to the University of Wisconsin—Madison DNA Sequencing facility for Illumina sequencing (Illumina HiSeq 2500) for single-end 1x50-bp reads per the manufacturer’s recommendations.

ChIP-seq data were analyzed as previously described (73, 74), with the following modifications. Sequence reads of low quality were removed from each file using Trimmomatic (version 0.30) using standard settings (75). Furthermore, the first and last five bases from each read were trimmed to remove the locations with the lowest average quality scores. This removed between 0.1% and 2.6% of reads. Filtered sequence reads were aligned to the published E. coli CFT073 genome (GenBank accession no. NC_004431.1) using the software package Bowtie 2 (version 2.2.2), using default settings (76). Sequence reads that did not align to the CFT073 genome at all or were aligned to more than one location were eliminated (an average of 15% of reads were removed). Areas of Fur enrichment were identified using MOSAiCS (version 1.6.0) with peaks with a false-discovery rate (FDR) of <0.05 being included as significant (77). For visualization, aligned BAM files were converted into WIG files using QuEST (version 1.2) using standard settings (78). All WIG files were visualized in MochiView (79). For identification of Fur ChIP-seq peaks in the promoters of genes, a cutoff of 19.5 was selected for peak summit height.

### Bioinformatic analysis.

To identify a Fur binding site in the promoter region of the CFT073-specific genes that had a Fur ChIP-seq peak, we searched ∼300 bp around the ChIP-seq peak summit for a Fur binding motif ( GATAATGATAATCATTATC) that was previously reported for strain MG1655 using the MEME suite (http://meme-suite.org/) (80) with standard settings and a q-value cutoff of <0.02. To identify possible RyhB pairing sites, sequences 200 bp upstream of the translational start site of the target genes were used to predict putative RyhB binding sites by Freiburg IntaRNA-tool (http://rna.informatik.uni-freiburg.de/IntaRNA/Input.jsp) using default settings.

### High-throughput RNA sequencing (RNA-seq).

Total RNA was isolated from three biological replicates each of wild-type CFT073 (WAM4505), CFT073Δ*fur* (WAM5491), CFT073Δ*ryhB* (WAM5497), and CFT073Δ*fur*Δ*ryhB* (WAM5499) strains grown anaerobically in MOPS minimal medium supplemented with 0.2% glucose sparged with a gas mix of 95% N_2_ and 5% CO_2_ until an OD_600_ of 0.25 was reached. Depletion of rRNA was performed using Ribozero rRNA removal kit (Bacteria probe) (Illumina, San Diego, CA, USA). Strand-specific RNA-sequencing libraries were prepared using NEBNext Ultra RNA Library Prep kit (NEB, Ipswich, MA, USA) per the manufacturer’s instructions. Briefly, enriched RNAs were fragmented for 15 min at 94°C. First and second strands of cDNA were sequentially synthesized, end repaired, adenylated at 3′ ends and universal adaptors were ligated, followed by index addition and library enrichment with limited cycle PCR. Sequencing libraries were validated using the Agilent TapeStation 4200 (Agilent Technologies, Palo Alto, CA, USA) and quantified by using a Qubit 2.0 fluorometer (Invitrogen, Carlsbad, CA) as well as by quantitative PCR (Applied Biosystems, Carlsbad, CA, USA) using the adaptor sequences as primers. The sequencing libraries were multiplexed and clustered in one lane of a flow cell. After clustering, the flow cell was loaded on the Illumina HiSeq instrument per the manufacturer’s instructions. The samples were sequenced using a 2 × 150 paired-end (PE) configuration. Image analysis and base calling were conducted by the HiSeq Control Software (HCS). Raw sequence data (.bcl files) generated from Illumina HiSeq were converted into fastq files and demultiplexed using Illumina’s bcl2fastq 2.17 software. One mismatch was allowed for index sequence identification. Library generation and sequencing were conducted at Genewiz (NJ, USA).

The reads from next-generation sequencing (NGS) were mapped to the complete CFT073 genome (NC004431.1) with the bwa-mem program (version 0.7.12) (81) using default settings. The mapped reads in SAM file format were converted to BAM file format using SAMtools (82). An average of 10% of reads from each data set did not align to the CFT073 genome and were removed. For visualization purposes, the aligned BAM files were converted to WIG files using QuEST (version 1.2) (78) and viewed in the MochiView genome browser (79). Differentially expressed genes were assigned to regulons using the regulatory information present in the EcoCyc database for E. coli K-12 (45) and ASAP database (83).

The transcript expression levels for all CFT073 genes were estimated using the software package RSEM (version 1.3.0) (84) using the Bowtie 2 aligner (76) to map reads against the set of all predicted CFT073 transcripts downloaded from the ASAP database (83). The expected read counts generated with RSEM (84) for all three replicates were used as an input for the DEseq2 software package (85) using the default settings on the RNA-seq 2G server (http://rnaseq2g.awsomics.org) to obtain the relative fold change of mutant versus wild-type transcript levels and the statistical significance of the changes for each gene. Genes that showed greater than 2.0-fold change in expression with a false-discovery rate (FDR) of <0.05 were labeled as differentially expressed genes in our analysis.

### Proteomic analysis.

Wild-type and mutant MG1655 and CFT073 strains were grown anaerobically in 1× MOPS minimal medium supplemented with 0.2% glucose at 37°C to an OD_600_ of 0.25. Cells were harvested at 7,550 × *g* for 10 min at 4^ο^C. Pelleted protein was resuspended in 200 μl lysis buffer [8 M urea, 100 mM Tris (pH 8.0), 20 mM Tris(2-carboxyethyl)phosphine (TCEP), 80 mM chloroacetamide] and subjected to three freeze-thaw cycles by submersing the tubes in liquid nitrogen. Samples were then diluted with 50 mM Tris (pH 8.0) to a concentration of 1.5 M urea. Protein digestion was performed overnight with trypsin (4 μg) before centrifuging for 5 min at 14,000 × *g* and desalting cleared supernatant with 10 mg Strata C_18_ solid-phase extraction cartridges. The resulting peptides were then quantified using a quantitative colorimetric peptide assay (Thermo Fisher Scientific) and dried in a vacuum centrifuge before resuspension in 0.2% formic acid.

Samples were analyzed using a liquid chromatography-mass spectrometry (LC-MS) instrument comprising an Orbitrap Fusion Lumos Tribrid mass spectrometer (Thermo Fisher Scientific). Mobile phase A consisted of 0.2% formic acid in water, and mobile phase B consisted of 0.2% formic acid in 70% acetonitrile. A 105-minute gradient ranging from 0% to 53% mobile phase B was employed spanning a total run time of 120 min. Analytes were injected onto a 1.7-μm C_18_ column packed in-house to a length of 35 cm and heated to 46°C. Survey scans of peptide precursors were collected from 300 to 1,350 Thomson with an AGC target of 1,500,000 and a resolution of 240,000 in the Orbitrap followed by higher-energy collisional dissociation (HCD) tandem mass spectrometry (MS/MS) scans taken at rapid speed in the ion trap.

The resulting LC-MS proteomic data were processed using Maxquant software version 1.5.2.8 and searched against a concatenated database of strains MG1655 and CFT073 using the ASAP database (83). The digestion enzyme was set to trypsin with up to two missed cleavages, carbamidomethylation of cysteines as a fixed modification, and oxidation of methionines and protein N-terminal acetylation as variable modifications. The match between runs feature was utilized to decrease missing data values within the data set. The precursor mass tolerance was 20 ppm, and product ions were searched at 4.5 ppm tolerances. Peptides were filtered to a 1% FDR and combined to protein groups based on the rules of parsimony. A cutoff of twofold change in protein abundance in Δ*fur* mutant compared to the wild-type strain was selected. There was a good correlation between our transcriptomic and proteomic data sets (*r* = 0.65).

### Western blotting.

Western blotting was conducted as previously described (32). Briefly, wild-type and Δ*fur*, Δ*ryhB*, and Δ*fur* Δ*ryhB* MG1655 and CFT073 strains (32; this study) were grown aerobically in LB or anaerobically in MOPS minimal medium with 0.2% glucose to an OD_600_ of 0.2. For some experiments, wild-type and mutant cells were also grown in the presence of an iron chelator, 1 mM diethylenetriamine pentaacetic acid (DTPA) or with all 20 amino acids, each having a final concentration of 100 μM. Cells (500 μl) were harvested, lysed in 10 μl of 1×  sodium dodecyl sulfate-polyacrylamide gel electrophoresis (SDS-PAGE) solubilization buffer, and proteins were resolved by 16% SDS-PAGE. The resolved proteins were transferred to nitrocellulose, and Western blotting was conducted using an affinity-purified primary antibody to Fur or RpoS and horseradish peroxidase (HRP)-tagged secondary antibody (diluted 1:5,000) (Santa Cruz Biotechnologies Inc., Dallas, TX) as previously described. The blots were visualized with an Azure Biosystems c600 imager using Western Lightning Plus-ECL detection system (Perkin Elmer Inc.) per the manufacturer’s instructions. In some experiments, the membrane was stripped with 62.mM Tris-Cl (pH 6.7), 2% SDS, 0.7% 2-mercaptoethanol and reprobed with affinity-purified polyclonal antibody against fumarate and nitrate reductase (FNR) (dilution, 1:10,000) that acted as an internal control. Band intensities were quantified using ImageJ software.

### Data availability.

ChIP-seq and RNA-Seq data sets were deposited at Gene Expression Omnibus (GEO) with the accession code (GSE145424), and raw MS data were deposited into PRIDE (project accession no. PXD017582).
