# Supplementary figures and images for: Tailoring a Global Iron Regulon to a Uropathogen
Source: mBio. 2020 Mar 24;11(2):e00351-20. doi: 10.1128/mBio.00351-20 (PMC7157518; doi:10.1128/mBio.00351-20)

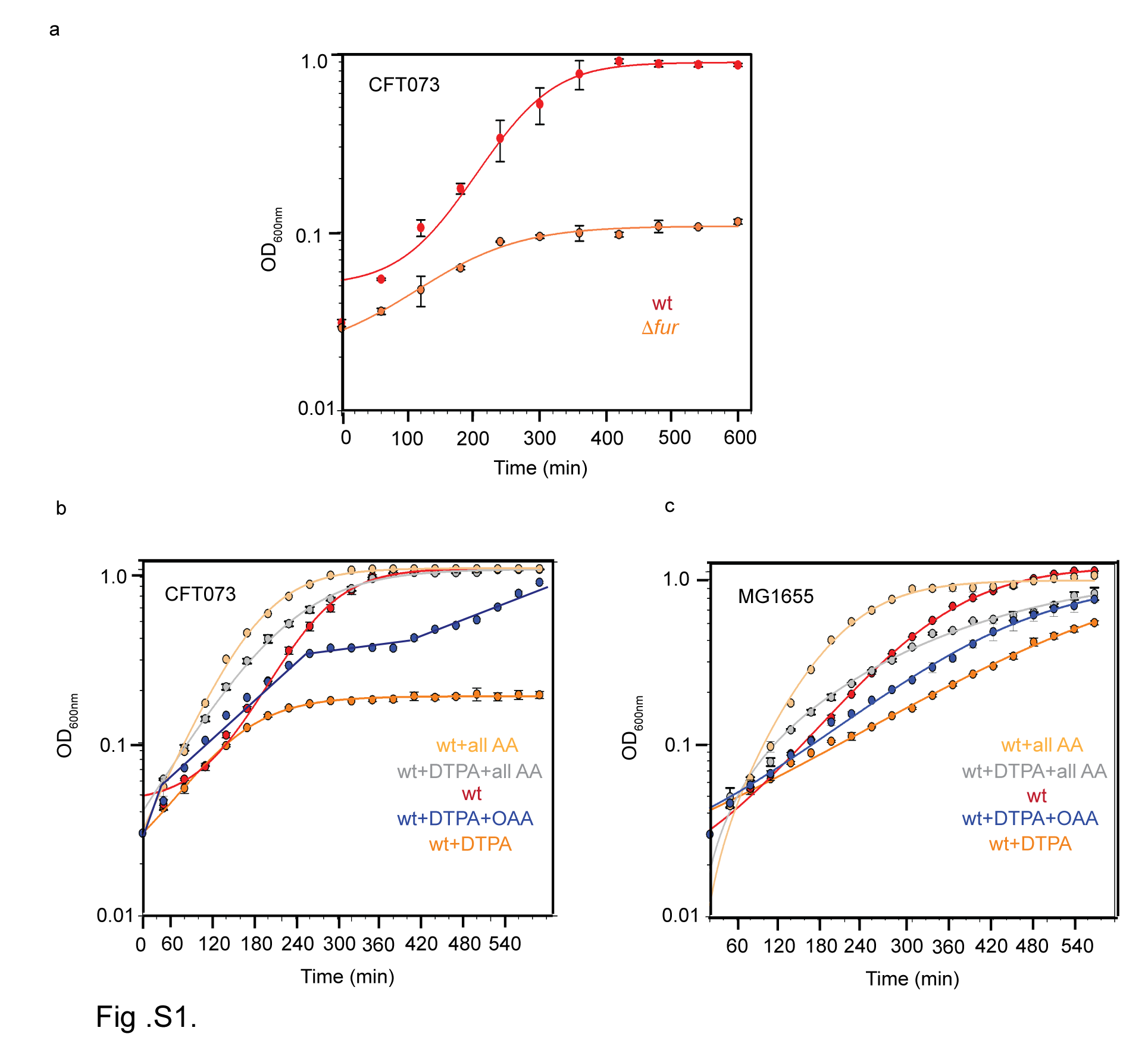

Supplement: FIG S1 [file mBio.00351-20-sf001.tif]

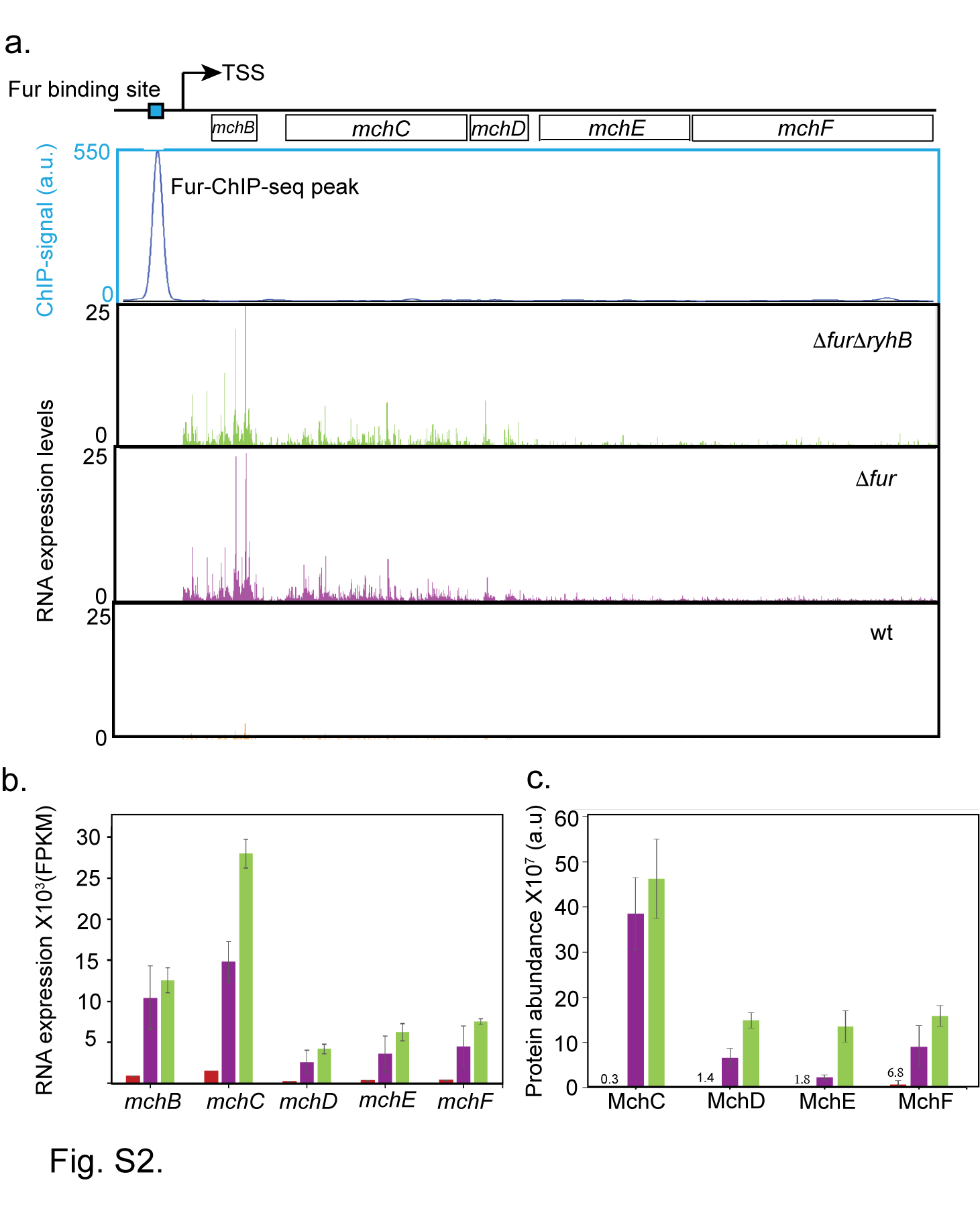

Supplement: FIG S2 [file mBio.00351-20-sf002.tif]

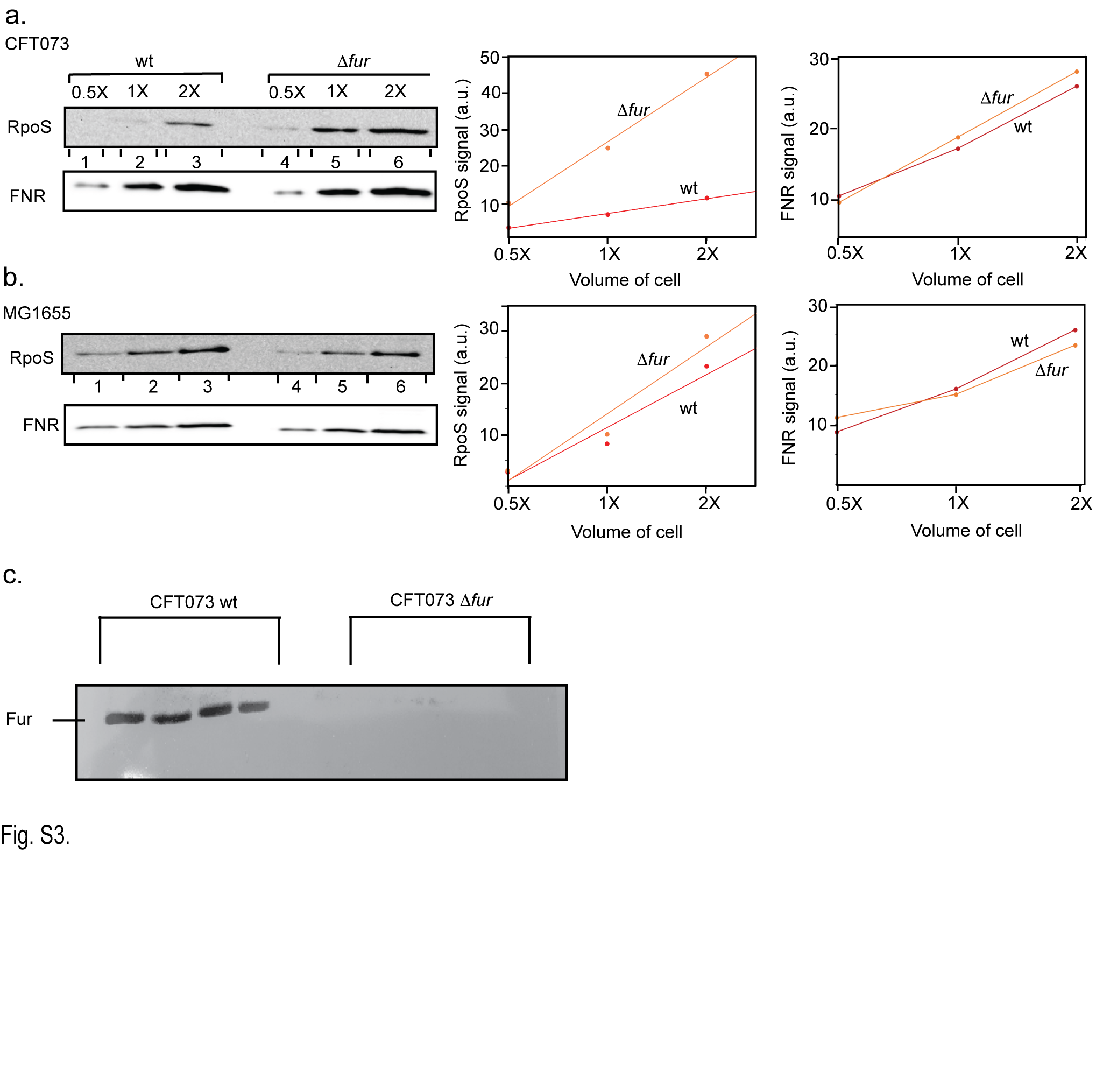

Supplement: FIG S3 [file mBio.00351-20-sf003.tif]

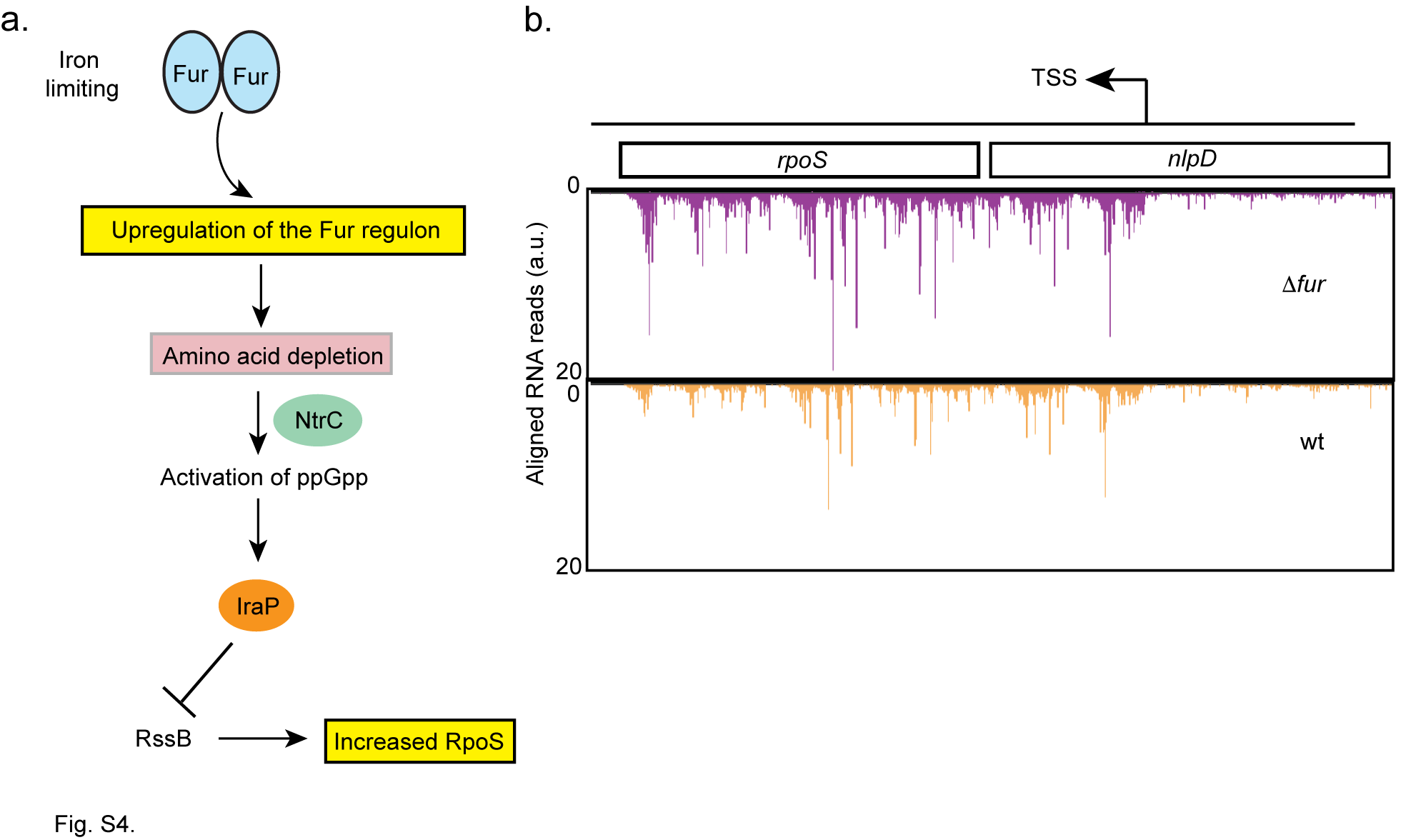

Supplement: FIG S4 [file mBio.00351-20-sf004.tif]
