# Supplementary material for: Tailoring a Global Iron Regulon to a Uropathogen
Source: mBio. 2020 Mar 24;11(2):e00351-20. doi: 10.1128/mBio.00351-20 (PMC7157518; doi:10.1128/mBio.00351-20)
Supplement: TABLE S2 [file mBio.00351-20-st002.pdf]

Table S2: Genes regulated by RyhB in both CFT073 and MG1655[1]

Operons regulated by RyhB in both CFT073 and MG1655, which encode at least one protein with iron as cofactor

Functions[2]	Operon[2]	Gene[2]	Protein product[2]	c-number[2]	RNA-seq fold change[3]		
					fur^{-}/wt	ryhB^{-}/wt	fur^{-}ryhB^{-}/wt
**Respiration and Metabolism**							
	dmsABC	***dmsA***	dimethyl sulfoxide reductase, anaerobic, subunit A	c1031	0.15	1.00	1.00
		dmsB	dimethyl sulfoxide reductase, anaerobic, subunit B	c1032	0.22	0.93	1.00
		dmsC	dimethyl sulfoxide reductase, anaerobic, subunit C	c1033	0.38	1.00	1.00
	hybOABCDEFG	***hybO***	hydrogenase 2, small subunit	c3734	0.38	1.07	0.93
		hybA	hydrogenase 2 4Fe-4S ferredoxin-type component	c3733	0.29	1.07	0.93
		hybB	predicted hydrogenase 2 cytochrome b type component	c3732	0.27	1.07	0.87
		hybC	hydrogenase 2, large subunit	c3731	0.41	1.07	0.87
		hybD	predicted maturation element for hydrogenase 2	c3730	0.41	1.00	0.81
		hybE	hydrogenase 2-specific chaperone	c3729	0.41	1.00	0.87
		hybF	protein involved with the maturation of hydrogenases 1 and 2	c3728	0.47	1.00	0.81
		hybG	hydrogenase 2 accessory protein	c3727	0.54	0.87	0.81
	hycABCDEFGHI	***hycA***	regulator of the transcriptional regulator FhlA	c3285	0.57	1.07	0.87
		hycB	hydrogenase 3, Fe-S subunit	c3284	0.57	1.07	0.76
		hycC	hydrogenase 3, membrane subunit	c3283	0.57	1.07	0.81
		hycD	hydrogenase 3, membrane subunit	c3282	0.66	1.07	0.87
		hycE	hydrogenase 3, large subunit	c3281	0.54	1.07	0.81
		hycF	formate hydrogenlyase complex iron-sulfur protein	c3280	0.54	1.07	0.87
		hycG	hydrogenase 3 and formate hydrogenase complex, HycG subunit	c3279	0.57	1.07	0.87
		hycH	protein required for maturation of hydrogenase 3	c3278	0.50	1.00	0.81
		hycI	protease involved in processing C-terminal end of HycE	c3277	0.57	1.00	0.81
	hypABCDE-fhlA	***hypA***	protein involved in nickel insertion into hydrogenases 3	c3286	0.57	1.00	0.76
		hypB	GTP hydrolase involved in nickel liganding into hydrogenases	c3287	0.41	1.07	1.07
		hypC	protein required for maturation of hydrogenases 1 and 3	c3288	0.44	1.00	1.00
		hypD	protein required for maturation of hydrogenases	c3289	0.44	1.00	0.93
		hypE	carbamoyl phosphate phosphatase, hydrogenase 3 maturation protein	c3290	0.57	1.00	0.93
		fhlA	DNA-binding transcriptional activator	c3291	0.71	1.00	0.87
	frdABCD	***frdA***	fumarate reductase (anaerobic) catalytic and NAD/flavoprotein subunit	c5242	0.57	1.07	1.41
		frdB	fumarate reductase (anaerobic), Fe-S subunit	c5241	0.54	1.07	1.41
		frdC	fumarate reductase (anaerobic), membrane anchor subunit	c5240	0.54	1.07	1.32
		frdD	fumarate reductase (anaerobic), membrane anchor subunit	c5239	0.50	1.00	1.32
	narGHJI	***narG***	nitrate reductase 1, alpha subunit	c1685	0.47	0.93	1.00
		narH	nitrate reductase 1, beta (Fe-S) subunit	c1686	0.54	0.93	1.00
		narJ	molybdenum-cofactor-assembly chaperone subunit (delta subunit) of nitr	c1687	0.57	0.93	0.93
		narI	nitrate reductase 1, gamma (cytochrome b(NR)) subunit	c1688	0.71	1.00	1.00
	nrfABCDEFG	***nrfA***	nitrite reductase, formate-dependent, cytochrome	c5066	0.13	1.00	1.41
		nrfB	nitrite reductase, formate-dependent, penta-heme cytochrome c	c5067	0.09	0.93	1.32
		nrfC	formate-dependent nitrite reductase, 4Fe4S subunit	c5068	0.11	0.93	1.23
		nrfD	formate-dependent nitrite reductase, membrane subunit	c5069	0.09	1.00	1.32
		nrfE	heme lyase (NrfEFG) for insertion of heme into c552, subunit NrfE	c5070	0.18	1.00	1.07
		nrfF	heme lyase (NrfEFG) for insertion of heme into c552, subunit NrfF	c5071	0.33	0.93	0.87
		nrfG	heme lyase (NrfEFG) for insertion of heme into c552, subunit NrfG	c5072	0.44	1.00	1.00
	ynfEFGH-dmsD	***ynfE***	oxidoreductase subunit	c1977	0.50	1.00	1.07
		ynfF	oxidoreductase subunit	c1978	0.14	1.07	0.87
		ynfG	oxidoreductase, Fe-S subunit	c1979	0.18	1.07	1.00
		ynfH	oxidoreductase, membrane subunit	c1981	0.19	1.00	0.93
		dmsD	twin-argninine leader-binding protein for DmsA and TorA	c1982	0.54	1.00	1.00
	sdhCDAB	***sdhC***	succinate dehydrogenase, membrane subunit, binds cytochrome b556	c0798	0.57	1.00	0.66

		sdhD	succinate dehydrogenase, membrane subunit, binds cytochrome b556	c0800	0.62	1.00	1.07
		sdhA	succinate dehydrogenase, flavoprotein subunit	c0801	0.54	1.00	0.93
		sdhB	succinate dehydrogenase, FeS subunit	c0802	0.54	1.00	0.93
	nuoABCEFGHIJKLMN	***nuoA***	NADH:ubiquinone oxidoreductase, membrane subunit A	c2829	0.54	0.93	0.93
		nuoB	NADH:ubiquinone oxidoreductase, chain B	c2828	0.47	1.00	1.00
		nuoC	NADH:ubiquinone oxidoreductase, chain C,D	c2827	0.38	1.00	1.00
		nuoE	NADH:ubiquinone oxidoreductase, chain E	c2826	0.47	1.00	1.07
		nuoF	NADH:ubiquinone oxidoreductase, chain F	c2825	0.44	1.00	1.00
		nuoG	NADH:ubiquinone oxidoreductase, chain G	c2824	0.38	1.00	1.00
		nuoH	NADH:ubiquinone oxidoreductase, membrane subunit H	c2823	0.35	1.00	1.00
		nuoI	NADH:ubiquinone oxidoreductase, chain I	c2822	0.38	1.00	1.00
		nuoJ	NADH:ubiquinone oxidoreductase, membrane subunit J	c2821	0.41	1.00	1.07
		nuoK	NADH:ubiquinone oxidoreductase, membrane subunit K	c2820	0.50	1.15	1.07
		nuoL	NADH:ubiquinone oxidoreductase, membrane subunit L	c2819	0.41	1.00	1.00
		nuoM	NADH:ubiquinone oxidoreductase, membrane subunit M	c2818	0.47	1.07	0.93
		nuoN	NADH:ubiquinone oxidoreductase, membrane subunit N	c2817	0.47	1.00	0.93
	ydiJIH	***ydiJ***	predicted FAD-linked oxidoreductase	c2082	0.35	1.00	0.93
		ydiI	esterase	c2081	0.54	1.00	0.93
		ydiH	predicted protein	c2080	1.87	1.00	0.87
	fumA	***fumA***	fumarate hydratase (fumarase A), aerobic Class I	c2004	0.41	1.00	0.81
	pflA	***pflA***	pyruvate formate lyase activating enzyme 1	c1038	0.62	1.00	1.23
**Unknown**	***yjiML***	***yjiM***	predicted 2-hydroxyglutaryl-CoA dehydratase	c5418	0.44	1.00	1.07
		yjiL	predicted ATPase, activator of (R)-hydroxyglutaryl-CoA dehydratase	c5417	0.38	1.00	0.93
**Fe-S assembly**							
	iscSUA-hscBA-fdx-iscX	***iscS***	cysteine desulfurase (tRNA sulfurtransferase), PLP-dependent	c3056	0.57	1.07	1.15
		iscU	FeS cluster assembly scaffold	c3055	0.76	1.07	1.07
		iscA	FeS cluster assembly protein	c3053	0.76	1.00	1.07
		hscB	DnaJ-homologue co-chaperone protein Hsc20	c3052	0.71	1.00	1.07
		hscA	DnaK-like molecular chaperone specific for IscU	c3051	0.66	1.00	1.23
		fdx	[2Fe-2S] ferredoxin	c3050	0.57	1.00	1.23
		iscX	conserved protein	c3049	0.62	1.07	1.15
	mrp	*mrp*	P-loop NTPase family protein mrp	c2641	0.54	1.00	0.87
**Oxidative stress**							
	sodB	***sodB***	superoxide dismutase, Fe	c2050	0.09	1.07	1.23
Genes regulated by RyhB in both CFT073 and MG1655 but lack iron as cofactor							
**Transport**							
	oppABCDF	***oppA***	periplasmic-binding component of an ABC superfamily oligopeptide trans	c1707	0.50	1.15	0.87
		oppB	oligopeptide transporter subunit; membrane component of ABC superfam	c1708	0.41	1.15	0.76
		oppC	membrane component of an ABC superfamily oligopeptide transporter	c1709	0.38	1.07	0.81
		oppD	oligopeptide transporter subunit; ATP-binding component of ABC superfa	c1710	0.44	1.15	0.76
		oppF	ATP-binding subunit of oligopeptide ABC transporter	c1711	0.41	1.07	0.76
	dppBCDF	***dppB***	dipeptide transporter; membrane component of ABC superfamily	c4358	0.22	1.07	1.23
		dppC	dipeptide transporter; membrane component of ABC superfamily	c4357	0.20	1.15	1.23
		dppD	dipeptide transporter; ATP-binding component of ABC superfamily	c4356	0.27	1.15	1.23
		dppF	dipeptide transporter; ATP-binding component of ABC superfamily	c4355	0.25	1.15	1.15
	ydeA	***sotB***	predicted arabinose transporter	c1950	2.14	1.07	1.07
	ynfM	***ynfM***	predicted transporter	c1987	2.83	0.93	0.71
	yohJK	***yohJ***	conserved inner membrane protein	c2673	2.00	0.93	1.23
		yohK	predicted inner membrane protein	c2674	0.87	0.93	1.00
	shiA	***shiA***	shikimate transporter	c2443	3.48	1.00	0.76
**Metabolism**							
	arnBCADTEF	***arnB***	UDP-4-amino-4-deoxy-L-arabinose alpha-ketoglutarate aminotransferase	c2795	0.31	0.93	0.93

		arnC	Undecaprenyl-phosphate 4-amino-4-deoxy-L-arabinose transferase	c2796	0.35	0.93	0.76
		arnA	bifunctional UDP-L-Ara4N formyltransferase/UDP-GlcA C-4'-decarboxylas	c2797	0.31	0.93	0.76
		yfbH	conserved protein	c2798	0.31	0.93	0.76
		arnT	4-amino-4-deoxy-L-arabinose transferase	c2799	0.27	0.93	0.71
		arnE	hypothetical protein	c2800	0.29	0.93	0.66
		arnF	Hypothetical protein yfbJ	c2801	0.35	0.93	0.76
	ilvBN	***ilvB***	acetolactate synthase I, large subunit	c4596	0.44	1.07	1.07
		ilvN	acetolactate synthase I, small subunit	c4595	0.54	1.07	1.07
	bioA	***bioA***	adenosylmethionine-8-amino-7-oxononanoate aminotransferase	c0853	2.30	1.07	1.15
**Unknown Transport**							
	psiE	***psiE***	predicted phosphate starvation inducible protein	c5001	4.00	1.07	1.00
	yjfY	***yjfY***	predicted protein	c5289	2.14	1.00	1.00

[1] CFT073 operons that have at least one gene regulated by RyhB in both CFT073 and MG1655 grown in glucose minimal media anaerobically.

[2] Gene functions, predicted operons, gene names, protein annotations and c numbers were obtained from EcoCyc.

[3] Fold change in RNA-seq expression of CFT073 Δ*fur* (WAM5491), Δ*ryhB* (WAM5497) or Δ*fur* Δ*ryhB* (WAM5499) compared to wild type (WAM4505).
Those genes which showed > 2-fold change (upregulation or down-regulation) in RNA-seq with p-value<0.05 in Δ*fur* but returned to wild type level in Δ*fur* Δ*ryhB* are indicated in bold.
