# Supplementary material for: Tailoring a Global Iron Regulon to a Uropathogen
Source: mBio. 2020 Mar 24;11(2):e00351-20. doi: 10.1128/mBio.00351-20 (PMC7157518; doi:10.1128/mBio.00351-20)
Supplement: TABLE S3 [file mBio.00351-20-st003.pdf]

Table S3: CFT073 Fur Indirect Regulon[1]

Genes whose expression is regulated by RpoS

Functions[2]	Operon[2]	Gene[2]	Protein product[2]	c-number[2]	Regulators[3]			RNA-seq fold change[4]		
								fur$^-$/wt	ryhB$^-$/wt	fur$^-$ryhB$^-$/wt
**Carbon metabolism**										
	talA-tktB	***talA***	Transaldolase A	c2989	RpoS			2.83	1.07	0.76
		tktB	Transketolase 2	c2990				3.03	1.07	0.76
	poxB	***poxB***	Pyruvate oxidase	c1004	RpoS			3.03	1.07	0.76
	dkgA	***dkgA***	Methylglyoxal reductase	c3746	RpoS			2.64	1.00	1.15
	tam	***tam***	Trans-aconitate methyltransferase	c1942	RpoS			2.14	1.07	0.81
	fbaB	***fbaB***	Fructose bisphosphate aldolase I	c2623	RpoS			2.14	1.07	0.87
	otsBA	***otsB***	Trehalose-6-phosphate phosphatase, biosynthetic	c2311	RpoS			2.30	1.00	0.62
		otsA	Trehalose-6-phosphate synthase	c2310				2.14	1.07	0.62
	treA	***treA***	Periplasmic trehalase	c1654	RpoS			2.14	1.00	0.87
	treF	***treF***	Cytoplasmic trehalase	c4330	RpoS			2.00	1.00	1.00
	hdhA	***hdhA***	7-α-hydroxysteroid dehydrogenase	c2011	RpoS			2.00	1.00	0.76
**Putrescine degradation**										
	gabDTP	***gabD***	Succinate-semialdehyde dehydrogenase I, NADP-dependent	c3209	RpoS	NtrC, Nac	ppGpp	2.14	1.00	0.81
		gabT	4-aminobutyrate aminotransferase, PLP-dependent	c3210				2.14	1.07	0.93
		gabP	Gamma-aminobutyrate transporter	c3211				2.14	1.07	0.93
	patA	***ygjG***	Putrescine aminotransferase	c3828	RpoS	NtrC		3.73	1.07	0.93
	ydcSTUVW	***ydcS***	Periplasmic-binding component of an ABC superfamily Predicted s	c1864	RpoS	Nac		2.46	1.00	0.87
		ydcT	ATP-binding component of an ABC superfamily Predicted spermid	c1865				2.00	1.00	0.87
		ydcU	Membrane component of an ABC superfamily Predicted spermidi	c1866				1.74	1.00	0.76
		ydcV	Membrane component of an ABC superfamily Predicted spermidi	c1867				ND	ND	ND
		ydcW	Medium chain aldehyde dehydrogenase	c1869				2.46	1.15	1.07
**Biofilm formation**										
	csgBA	***csgB***	Curlin, minor subunit	c1305	RpoS			5.66	1.07	0.87
		csgA	Curlin, major subunit	c1306				3.25	1.15	1.00
	csgC	***csgC***	Predicted curli production protein	c1307	RpoS			2.46	1.00	1.00
	wrbA-yccJ	***wrbA***	NADH:quinone oxidoreductase flavoprotein	c1140	RpoS			2.30	1.00	0.76
		yccJ	Conserved protein	c1139				2.30	1.00	0.71
	mlrA	***mlrA***	DNA binding transcriptional activator	c2657	RpoS			2.83	1.07	0.76
	glgS	***glgS***	Predicted glycogen synthesis protein	c3797	RpoS			2.14	0.93	0.93
**Acid resistance**										
	hdeAB-yhiD	***hdeA***	Periplasmic acid stress chaperone	c4321	RpoS			8.00	1.00	1.23
		hdeB	Periplasmic acid stress chaperone	c4320				8.57	1.00	1.32
		yhiD	Predicted Mg(2+) transport ATPase inner membrane protein	c4319				7.46	1.00	1.52
	hdeD	***hdeD***	Acid stress membrane protein	c4322	RpoS			6.96	1.00	0.81
	gadBC	***gadB***	Glutamate decarboxylase B	c1922	RpoS			7.46	1.00	0.87
		gadC	L-glutamate:4-aminobutyrate antiporter	c1921				8.00	1.00	1.15
	ybaST	***ybaS***	Glutaminase I	c0605	RpoS	GadX		6.50	1.00	0.76
		ybaT	Putative transporter	c0606				3.73	0.93	0.76

gadE-mdtEF	***gadE***	DNA binding transcriptional activator	c4323	RpoS			6.50	1.00	0.81
	mdtE	Multi-drug efflux pump fusion protein	c4324				6.06	1.00	1.07
	mdtF	Multi-drug efflux pump RND permease	c4325				5.66	1.00	1.00
gadAXW	***gadA***	Glutamate decarboxylase A	c4328	RpoS			6.50	0.93	0.76
	gadX	DNA binding transcriptional dual regulator	c4327				3.03	0.93	0.87
	gadW	DNA binding transcriptional dual regulator	c4326				3.03	0.93	0.93
**Transport**									
blc	***blc***	Outer membrane protein	c5237	RpoS			2.30	1.00	0.81
**Stress response**									
sodC	***sodC***	Superoxide dismutase (Cu-Zn)	c2038	RpoS			2.64	1.00	0.81
osmB	***osmB***	Osmotically induced lipoprotein	c1753	RpoS		ppGpp	3.48	1.00	1.41
osmC	***osmC***	Osmotically inducable peroxiredoxin	c1916	RpoS			3.25	1.07	0.71
osmY	***osmY***	Periplasmic chaperone	c5457	RpoS			2.14	1.00	0.50
mysB	***msyB***	Heat stress response	c1318	RpoS			3.03	1.00	0.87
uspB	***uspB***	Universal stress protein	c4292	RpoS		ppGpp*	2.83	1.07	0.81
yfcG	***yfcG***	Disulfide reductase	c2845	RpoS			2.46	1.07	0.66
elaB	***elaB***	Stress response protein	c2810	RpoS			2.30	1.07	0.62
kbp	***ygaU***	K+ binding protein	c3213	RpoS		ppGpp	3.25	1.00	0.81
yjiY	***yjiY***	Pyruvate:H+ symporter	c5429	RpoS			6.96	0.87	1.15
hchA	***hchA***	Hsp31 molecular chaperone	c2385	RpoS		ppGpp*	2.30	1.07	0.71
cbpAM	***cbpA***	Curved DNA binding protein	c1136	RpoS	Fis	ppGpp	2.14	1.00	1.07
	cbpM	Chaperone modulator	c1135				2.00	1.00	1.07
**Unknown function**									
yhjG	***yhjG***	Hypothetical protein	c4335	RpoS			2.46	1.00	0.81
yegS	***yegS***	Putative lipid kinase	c2614	RpoS			2.14	1.00	0.57
dsrB	***dsrB***	Hypothetical protein	c2370	RpoS			2.00	1.00	0.87
yfhG-fic	*yhfG*	Hypothetical protein	c3078	RpoS			1.52	1.00	0.81
	fic	stationary-phase protein, cell division	c4136				2.64	0.93	0.81
yqjCDEK	***yqjC***	Hypothetical protein	c3855	RpoS			2.00	1.00	0.87
	yqjD	Hypothetical protein	c3856				2.46	1.07	0.87
	yqjE	Hypothetical protein	c3857				2.14	1.00	0.93
	yqjK	Hypothetical protein	c3858				2.14	1.00	0.93
Genes whose expression is regulated by ppGpp									
**RpoS protein stabilization**									
iraP	***iraP***	Anti adapter protein	c0489	ppGpp			2.83	1.07	1.07
**Stress response**									
yiiST	***yiiS***	DUF406 domain containing protein	c4874	ppGpp			2.30	1.07	1.07
	yiiT	Universal stress protein	c4875				2.30	1.07	1.00
sbmC	***sbmC***	DNA gyrase inhibitor	c2537	ppGpp*			2.14	1.00	1.07
ygaM	***ygaM***	Oxidative stress response	c3223	ppGpp*			2.46	1.00	0.76
ybdK	***ybdK***	Gamma-glutamyl:cysteine ligase	c0667	ppGpp			2.30	0.93	0.81
**Carbon metabolism**									
ggt	***ggt***	Glutathione hydrolase	c4236	ppGpp			2.00	1.07	0.71
ynhG	***ynhG***	Transpeptidase	c2073	ppGpp			2.83	1.00	0.93
pyrLBI	*pyrL*	PyrBI operon leader peptide	c5502	ppGpp			1.00	1.00	1.00
	pyrB	Aspartate carbamoyltransferase, catalytic subunit	c5345				0.50	1.00	0.87
	pyrI	Aspartate carbamoyltransferase, regulatory subunit	c5344				0.50	1.07	0.81
**Transport**									
yehZY	***yehZ***	Predicted transporter subunit: periplasmic-binding component of	c2661	ppGpp			2.00	1.00	0.71

	yehY	Predicted transporter subunit: membrane component of ABC supe	c2660				1.52	1.00	0.66
**Unknown function**									
yegP	***yegP***	Hypothetical protein	c2606	ppGpp			3.73	1.07	0.66
ybhP-clsB-ybhN	***ybhP***	Predicted DNase	c0873	ppGpp			2.14	0.93	0.66
	ybhO	Cardiolipin synthase 2	c0872				2.14	1.00	0.62
	ybhN	Conserved inner membrane protein	c0871				2.14	1.00	0.76
ybaA	***ybaA***	Hypothetical protein	c0574	ppGpp	CsgD		3.73	1.07	1.07
yohC	***yohC***	Putative inner membrane protein	c2667	ppGpp			3.48	1.15	1.00
yhhA	***yhhA***	Hypothetical protein	c4237	ppGpp			3.25	1.07	0.87
ybgS	***ybgS***	Hypothetical protein	c0829	ppGpp			2.64	1.00	0.62
yahO	***yahO***	Hypothetical protein	c0449	ppGpp			2.83	1.07	0.62
yccT	***yccT***	Hypothetical protein	c1101	ppGpp	CsgD		2.46	1.07	0.66
yjfY	***yjfY***	Hypothetical protein	c5289	ppGpp			2.14	1.00	1.00
ybeL	***ybeL***	Hypothetical protein	c0734	ppGpp			2.14	1.00	0.93
yniA	***yniA***	Putative kinase	c2124	ppGpp			2.00	1.00	1.00
yebF	***yebF***	Secreted protein	c2259	ppGpp			2.00	1.00	1.15
yhcO	***yhcO***	Putative barnase inhibitor	c3994	ppGpp			2.64	1.00	0.87
Genes whose expression is regulated by NtrC									
**Nitrogen metabolism**									
rutABCDEFG	***ycdM***	Pyrimidine oxygenase	c1149	NtrC (yeaG)			5.66	1.00	2.00
	ycdL	Peroxyureidoacrylate/ureidoacrylate aminohydrolase	c1148				4.59	1.00	1.41
	ycdK	Putative aminoacrylate peracid reductase	c1147				4.59	1.00	1.52
	ycdJ	Putative aminoacrylate hydrolase	c1146				3.73	1.00	1.52
	ycdI	Putative malonic semialdehyde reductase	c1145				3.25	1.00	1.07
	ycdH	Flavin reductase	c1144				2.64	1.00	1.15
	ycdG	Pyrimidine:H+ symporter	c1143				2.14	1.00	1.00
nac	***nac***	DNA-binding transcriptional dual regulator of nitrogen assimilatio	c2446	NtrC			3.48	1.00	2.83
glnK-amtB	***glnK***	Nitrogen assimilation regulatory protein for GlnL, GlnE, and AmtB	c0568	NtrC	GadX		5.28	1.00	3.73
	amtB	Ammonium transporter	c0570				6.06	1.07	4.00
yeaGH	***yeaG***	Protein kinase	c2188	NtrC			4.00	1.00	1.23
	yeaH	DUF444 domain containing protein	c2189				4.29	1.00	1.23
yhdVWXYZ	*yhdV*	Predicted outer membrane protein	c4033	NtrC			1.52	0.93	2.30
	yhdW	Putative amino-acid ABC transporter binding protein yhdW precur	c4034				1.52	1.07	2.30
	yhdX	Membrane component of an ABC superfamily predicted amino-ac	c4035				2.30	1.00	2.46
	yhdY	Membrane component of an ABC superfamily predicted amino-ac	c4036				2.46	1.00	2.14
	yhdZ	ATP-binding component of an ABC superfamily predicted amino-a	c4037				2.46	1.00	1.87
**Putrescine metabolism**									
potFGHI	***potF***	Putrescine transporter subunit: periplasmic-binding component of	c0987	NtrC			2.64	1.07	1.32
	potG	Putrescine transporter subunit: ATP-binding component of ABC su	c0988				2.46	1.07	1.23
	potH	Putrescine transporter subunit: membrane component of ABC sup	c0989				2.46	1.07	1.15
	potI	Putrescine transporter subunit: membrane component of ABC sup	c0990				2.14	1.00	1.07
Genes whose expression is regulated by miscellaneous regulators									
**Carbon metabolism**									
citCDEFXG	***citC***	Citrate lyase synthase	c0709	CitB			14.93	1.00	1.32
	citD	Citrate lyase, acyl carrier (gamma) subunit	c5685				9.85	1.00	1.07
	citE	Citrate lyase, citryl-ACP lyase (beta) subunit	c0706				18.38	1.00	1.52
	citF	Citrate lyase, citrate-ACP transferase (alpha) subunit	c0704				16.00	1.00	1.74
	citX	Apo-citrate lyase phosphoribosyl-dephospho-CoA transferase	c0702				13.93	1.00	1.62

	citG	Triphosphoribosyl-dephospho-CoA transferase	c0701				14.93	1.00	1.07
citT	***citT***	Citrate:succinate antiporter	c0700				9.19	1.07	1.41
citAB	***citA***	Sensory histidine kinase in two-component regulatory system wit	c0710	CitB			2.14	0.93	1.23
	citB	DNA-binding response regulator in two-component regulatory sys	c0711				2.64	1.00	1.15
yajO	***yajO***	1-deoxyxylulose 5-phosphate synthase	c0530	Lrp			2.14	1.07	0.87
ugd	***ugd***	UDP-glucose 6-dehydrogenase	c2555	Nac			0.35	0.87	0.76
yjhT	***yjhT***	N-acetylneuraminate mutarotase	c5388	Crp			2.00	0.93	0.66
**Ariginine biosynthesis**									
argA	***argA***	N-acetylglutamate synthase	c3412	ArgR			3.03	0.93	1.52
argBCH	***argC***	N-acteylglutamyl phosphate reductase	c4917	ArgR			2.83	0.93	1.32
	argB	Acetylglutamate synthase	c4918				2.83	1.00	1.41
	argH	Argininosuccinate lyase	c4919				2.64	0.93	1.41
argD	***argD***	N-acetylornithine aminotransferase	c4134	ArgR			2.30	0.93	1.41
argE	*argE*	N-acteylornithine deacetylase	c4916	ArgR			1.87	1.00	1.15
argG	***argG***	Argininosuccinate synthetase	c3929	ArgR			2.30	1.00	1.32
argI	***argI***	Ornithine carbamoyltransferase 1	c5353	ArgR			4.00	0.93	1.52
artJ	***artJ***	Arginine-binding periplasmic protein 2 precursor	c0993	ArgR			3.25	1.00	1.15
**Stress response**									
spy	***spy***	Envelope stress induced periplasmic protein	c2143	BaeR			4.92	1.15	4.92
cstA	***cstA***	Carbon starvation protein	c0685	Crp			4.92	1.00	6.96
mdtABCD-baeSR	***mdtA***	Multidrug efflux system, subunit A	c2600	BaeR	CpxR		2.30	1.00	2.30
	mdtB	Multidrug efflux system, subunit B	c2601				1.74	1.00	2.00
	mdtC	Multidrug efflux system, subunit C	c2602				1.74	0.93	1.74
	mdtD	Multidrug efflux system protein	c2603				1.62	1.00	1.62
	baeS	Sensory histidine kinase in two-component regulatory system wit	c2604				1.87	1.00	1.52
	baeR	DNA-binding response regulator in two-component regulatory sys	c2605				1.32	1.00	1.00
**Nitrogen metabolism**									
metF	***metF***	5,10-methylenetetrahydrofolate reductase	c4899	MetJ			0.44	1.15	0.33
**Transport**									
sbp	***sbp***	sulfate transporter subunit; periplasmic-binding component of AB	c4869				0.38	1.07	0.44
**Lipid remodelling**									
eptA-basRS	***eptA***	Phosphoethanolamine transferase	c5119				0.25	0.93	0.54
	basR	DNA-binding response regulator in two-component regulatory sys	c5118				0.35	1.00	0.62
	basS	Sensor histidine kinase	c5117				0.35	0.93	0.57
**Nucleotide metabolism**									
codBA	***codB***	Cytosine transporter	c0455	PurR	Nac		0.54	1.07	0.93
	codA	Cytosine/isoguanine deaminase	c0456				0.62	1.07	1.00
caiF	***caiF***	DNA-binding transcriptional activator	c0043	H-NS	NarL		0.47	1.07	1.07
cdd	***cdd***	Cytidine/deoxycytidine deaminase	c2675	Crp			2.14	1.07	0.87
**Stress response**									
psiF	***psiF***	Phosphate starvation inducible protein	c0491	PhoB			2.46	0.93	0.81
ivy	***ivy***	Periplasmic chaperone, inhibitor of vertebrate C-type lysozyme	c0370		Nac		4.00	1.00	1.00
yfiD	***yfiD***	Stress induced alternative pyruvate-formate lyase subunit	c3103	ArcA	Crp	Fis	2.30	1.00	1.32
**Unknown function**									
ycaC	***ycaC***	Putative hydrolase	c1034	BaeR			3.03	1.00	0.66
Genes whose expression is regulated by unknown regulators/mechanisms									
**RpoS protein stabilization**									
iraL	***ycgW***	Anti adapter protein	c1429				2.00	1.00	1.23
**Stress response**									

yodD	***yodD***	Stress induced protein	c2372				3.48	1.00	0.87
aqpZ	***aqpZ***	Water channel	c1009				3.48	1.07	1.15
yedYZ	***yedY***	Periplasmic protein-L-methionine sulfoxide reductase heme bindi	c2389				2.00	1.00	1.07
	yedZ	Periplasmic protein-L-methionine sulfoxide reductase catalytic su	c2390				1.52	0.93	0.76
tsgA	***tsgA***	Putative transporter	c4139				2.00	1.00	1.15
**Biofilm formation**									
bssR	***bssR***	Regulator of biofilm formation	c0921				3.73	1.00	1.07
yjgK	***yjgK***	DUF386 domain containing toxin-antotoxin biofilm protein	c5352				0.50	1.00	0.93
flu	***flu***	Ag43a, antigen 43 phase-variable biofilm formation autotranspor	c1273				1.74	0.93	1.15
**Carbon metabolism**									
ldhA	***ldhA***	Lactate dehdydrogenase A	c1827				2.00	1.07	1.00
tdh	***tdh***	Threonine dehydrogenase	c4443				2.00	1.07	1.32
ybiM	***ybiM***	DUF1471 domain containing protein	c0891				2.83	1.00	1.00
amyA	***amyA***	α-amylase	c2342				2.30	1.00	0.71
malEFG	***malE***	Periplasmic-binding component of an ABC superfamily maltose tr	c5004				0.38	1.07	1.23
	malF	Membrane component of an ABC superfamily maltose transporte	c5003				0.54	1.07	1.23
	malG	Membrane component of an ABC superfamily maltose transporte	c5002				0.54	1.07	1.32
malK-lamB-malM	***malK***	ATP-binding component of an ABC superfamily maltose transport	c5005				0.47	1.07	1.62
	lamB	Maltose outer membrane porin (maltoporin)	c5006				0.50	1.07	2.00
	malM	Maltose regulon periplasmic protein	c5007				0.47	1.00	1.62
rihA	***rihA***	Pyrimidine specific ribonucleoside hydrolase	c0735				0.44	1.00	1.15
aroH	***aroH***	3-deoxy-D-arabino-heptulosonate-7-phosphate synthase, tryptoph	c2100				0.50	1.00	0.62
idi	***idi***	Isopentenyl diphosphate isomerase	c3467				1.62	1.00	0.81
**Transport**									
ompX	***ompX***	Outer membrane protein	c0900				2.46	1.00	1.23
chaBC	***chaB***	Predicted cation regulator	c1677				2.14	1.00	0.81
	chaC	Glutathione specific-g-glutamylcyclotransferase	c1678				2.00	1.00	0.81
**Nitrogen metabolism**									
pyrE	***pyrE***	Orotate phosphoribosyltransferase	c4466				0.41	1.00	0.54
**Lipid remodelling**									
lpxP	***lpxP***	Palmitoleoyl acyltransferase	c2915				0.50	1.00	0.93
eptC	***yijP***	Phosphoethanolamine transferase	c4914				0.47	1.00	0.93
**Unknown function**									
ymgE	***ymgE***	Predicted inner membrane protein	c1645				3.73	1.00	1.00
ymdF	***ymdF***	Hypothetical protein	c5722				3.73	1.00	1.07
ydiZ	***ydiZ***	Hypothetical protein	c2123				2.64	1.07	0.93
yphA	***yphA***	Hypothetical protein	c3065				2.46	1.07	0.81
yedR	***yedR***	Putative inner membrane protein	c2382				2.14	1.00	0.81
ydhS	***ydhS***	Hypothetical protein	c2060				2.14	1.00	0.71
yhjY	***yhjY***	Putative outer membrane protein	c4366				2.00	1.07	0.93
yahK	***yahK***	Predicted oxidoreductase, Zn-dependent and NAD(P)-binding	c0447				1.74	1.00	0.66
ycjY	***ycjY***	Predicted hydrolase	c1801				1.41	1.07	0.87
mqo	***mqo***	malate dehydrogenase, FAD/NAD(P)-binding domain	c2751				3.25	1.00	3.03
c1542	***c1542***	Lambda ant-restriction protein	c1542				0.50	1.00	1.07
CFT073 specific genes									
**Attachment to host**									
papBAHCDJKEFG	***papG***	PapG protein	c3583	PapX			0.62	0.87	0.57
	papF	PapF protein	c3584				0.44	0.93	0.54
	papE	PapE protein	c3585				0.57	0.87	0.57

	papK	PapK protein	c3586				0.62	0.87	0.66
	papJ	PapJ protein	c3588				0.76	0.93	0.71
	papD	PapD protein	c3589				1.00	1.00	0.76
	papC	PapC protein	c3590				0.50	0.93	0.62
	papH	PapH protein	c3591				0.38	0.93	0.50
	papA	PapA protein	c3592				0.33	0.93	0.38
	papB	PapB protein	c5636				0.35	1.00	0.33
papBAHCDJKEFG (2	***papG_2***	PapG protein	c5179	PapX			0.50	0.87	0.57
	papF_2	PapF protein	c5180				0.44	0.93	0.54
	papE_2	PapE protein	c5181				0.57	0.87	0.57
	papK_2	PapK protein	c5182				0.62	0.87	0.66
	papJ_2	PapJ protein	c5184				0.76	0.93	0.71
	papD_2	PapD protein	c5185				0.66	0.93	0.66
	papC_2	PapC protein	c5186				0.54	0.93	0.62
	papH_2	PapH protein	c5187				0.33	0.93	0.44
	papA_2	PapA protein	c5188				0.29	0.93	0.35
	papB_2	PapB protein	c5637				0.47	0.81	0.47
ksl(k2)C	***ksl(k2)C***	Predicted glycosyl transferase	c3694				0.54	1.15	0.71
**Transport**									
c0334	***c0334***	putative integral membrane protein	c0334				0.38	0.93	0.93
c0335	***c0335***	Hypothetical protein	c0335				0.47	0.93	0.93
c0336	***c0336***	PTS system, mannitol (Cryptic)-specific IIA component (EIIA-(C)MT	c0336				0.35	0.93	0.93
c2348	***c2348***	Outer membrane porin protein nmpC precursor	c2348				0.41	1.00	1.41
**Transposition**									
c5167	***c5167***	Putative transposase for IS629	c5167				0.14	1.00	1.00
**Unknown function**									
c0946	***c0946***	Hypothetical protein	c0946				0.38	1.00	0.81
c3276	***c3276***	Hypothetical protein	c3276				0.41	0.93	0.71
c5426	***c5426***	Hypothetical protein	c5426				0.44	1.00	0.71
c0933	***c0933***	Hypothetical protein	c0933				0.54	1.07	0.76
c2454	***c2454***	C-terminal fragment of a putative peptide synthetase (pseudogen	c2454				ND	ND	ND
c1536	***c1536***	Putative recombination protein Bet of prophage	c1536				0.81	1.07	3.03
c1583	***c1583***	Putative tail component of prophage	c1583				1.07	1.15	2.83
c2457	***c2457***	Putative amidase	c2457				1.74	0.93	1.32
c0261	***c0261***	Hypothetical protein	c0261				2.00	0.93	1.07

[1]CFT073 operons that do not have a Fur ChIP-seq peak in the upstream regulatory region and a predicted RyhB binding site in the 5'UTR but showed differential RNA expression in our experiments.

[2]Gene functions, predicted operons, gene names, protein annotations and c numbers are obtained from Ecocyc.

[3]Regulators that were previously reported to regulate these genes from Ecocyc and (47).

[4]Fold change in RNA expression of CFT073 Δ*fur* (WAM5491), Δ*ryhB* (WAM5497) or Δ*fur* Δ*ryhB* (WAM5499) compared to wild type (WAM4505), obtained from the RNA-seq dataset.
A cut-off of 2-fold in the change in RNA expression in Δfur (upregulation or down-regulation) with $p<0.05$ are marked in bold.
ND=Not detected in our dataset.
DNA transcription factors are marked in red.
