# Supplementary material for: Tailoring a Global Iron Regulon to a Uropathogen
Source: mBio. 2020 Mar 24;11(2):e00351-20. doi: 10.1128/mBio.00351-20 (PMC7157518; doi:10.1128/mBio.00351-20)
Supplement: TABLE S4 [file mBio.00351-20-st004.pdf]

**Table S4: Proteomics dataset of differentially expressed proteins[1] comparing *wt, fur, ryhB* and *fur ryhB* mutants of CFT073 and MG1655.**

*Orthologous proteins similarly regulated in both CFT073 and MG1655 Δfur strains*

| Gene[2] | Protein product[2] | c-number[2] | CFT073 Proteomics fold change[3] | | | | | | MG1655 Proteomics fold change[4] | | | | | |
|---|---|---|---|---|---|---|---|---|---|---|---|---|---|---|
| | | | fur$^-$/wt | p-value | ryhB$^-$/wt | p-value | fur$^-$ryhB$^-$/wt | p-value | fur$^-$/wt | p-value | ryhB$^-$/wt | p-value | fur$^-$ryhB$^-$/wt | p-value |
| ***aegA*** | predicted oxidored | c2995 | 0.278 | 0.000 | 1.050 | 0.788 | 0.881 | 0.325 | 0.149 | 0.023 | 0.802 | 0.492 | 0.904 | 0.620 |
| ***bioA*** | adenosylmethionin | c0853 | 2.875 | 0.001 | 0.836 | 0.285 | 0.634 | 0.012 | 2.768 | 0.004 | 0.979 | 0.920 | 1.027 | 0.893 |
| ***bioB*** | biotin synthase | c0855 | 0.409 | 0.000 | 0.922 | 0.189 | 0.678 | 0.001 | 0.457 | 0.002 | 1.024 | 0.816 | 1.075 | 0.510 |
| ***csgD*** | DNA-binding transc | c1302 | 9.030 | 0.030 | 0.655 | 0.581 | 2.526 | 0.237 | 20.618 | 0.016 | 4.142 | 0.169 | 7.684 | 0.053 |
| ***cspA*** | major cold shock p | c4377 | 0.097 | 0.007 | 0.508 | 0.282 | 0.307 | 0.056 | 0.338 | 0.003 | 0.744 | 0.485 | 0.701 | 0.432 |
| ***cspG*** | DNA-binding transc | c1123 | 0.022 | 0.002 | 0.589 | 0.353 | 0.290 | 0.043 | 0.295 | 0.002 | 0.589 | 0.299 | 1.050 | 0.549 |
| ***cysI*** | sulfite reductase, b | c3322 | 0.303 | 0.011 | 0.918 | 0.733 | 0.622 | 0.160 | 0.399 | 0.000 | 1.042 | 0.414 | 1.088 | 0.499 |
| ***dmsB*** | dimethyl sulfoxide | c1032 | 0.125 | 0.001 | 0.972 | 0.884 | 1.077 | 0.752 | 0.083 | 0.012 | 1.241 | 0.519 | 1.533 | 0.057 |
| ***dppD*** | dipeptide transport | c4356 | 0.159 | 0.000 | 0.981 | 0.701 | 0.895 | 0.062 | 0.141 | 0.001 | 1.025 | 0.804 | 1.079 | 0.231 |
| ***dppF*** | dipeptide transport | c4355 | 0.138 | 0.000 | 1.034 | 0.456 | 0.936 | 0.314 | 0.043 | 0.015 | 1.053 | 0.633 | 1.175 | 0.151 |
| ***dps*** | Fe-binding and sto | c0898 | 0.197 | 0.000 | 0.757 | 0.127 | 0.146 | 0.002 | 0.064 | 0.000 | 0.932 | 0.651 | 0.062 | 0.000 |
| ***efeB*** | redox component c | c1157 | 14.631 | 0.001 | 0.643 | 0.246 | 11.859 | 0.001 | 40.688 | 0.001 | 2.161 | 0.170 | 31.514 | 0.001 |
| ***efeO*** | component of a tri | c1156 | 21.198 | 0.000 | 0.980 | 0.859 | 14.973 | 0.000 | 42.501 | 0.010 | 0.782 | 0.150 | 19.930 | 0.000 |
| ***entA*** | 2,3-dihydro-2,3-dih | c0683 | 8.954 | 0.002 | 0.120 | 0.089 | 10.735 | 0.001 | 27.835 | 0.000 | 0.143 | 0.015 | 23.848 | 0.000 |
| ***entB*** | isochorismatase/e | c0682 | 328.080 | 0.000 | 1.260 | 0.637 | 340.273 | 0.000 | 204.746 | 0.000 | 0.272 | 0.015 | 196.763 | 0.000 |
| ***entC*** | isochorismate synt | c0680 | 492.907 | 0.000 | 0.374 | 0.190 | 647.091 | 0.000 | 2218.599 | 0.001 | 0.500 | 0.590 | 2756.070 | 0.001 |
| ***entE*** | Enterobactin synth | c0681 | 147.917 | 0.000 | 0.504 | 0.105 | 180.298 | 0.000 | 15.159 | 0.000 | 0.242 | 0.032 | 11.594 | 0.000 |
| ***entF*** | enterobactin synth | c0673 | 564.156 | 0.000 | 1.182 | 0.791 | 534.714 | 0.000 | 313.373 | 0.000 | 0.760 | 0.793 | 485.988 | 0.000 |
| ***exbB*** | membrane spannin | c3741 | 7.262 | 0.006 | 0.698 | 0.459 | 2.028 | 0.151 | 37.260 | 0.000 | 0.957 | 0.614 | 43.104 | 0.000 |
| ***exbD*** | membrane spannin | c3740 | 41.224 | 0.000 | 0.881 | 0.776 | 24.689 | 0.000 | 355.772 | 0.000 | 0.776 | 0.506 | 400.541 | 0.000 |
| ***fdx*** | [2Fe-2S] ferredoxin | c3050 | 0.410 | 0.001 | 1.053 | 0.633 | 0.897 | 0.458 | 0.240 | 0.004 | 0.656 | 0.327 | 1.146 | 0.624 |
| ***feoA*** | ferrous iron transp | c4185 | 13.952 | 0.000 | 0.245 | 0.368 | 18.133 | 0.000 | 116.212 | 0.000 | 1.405 | 0.150 | 123.894 | 0.000 |
| ***fepA*** | iron-enterobactin c | c0669 | 8.780 | 0.003 | 0.944 | 0.825 | 3.156 | 0.010 | 1807.144 | 0.000 | 0.151 | 0.111 | 1413.436 | 0.000 |
| ***fepB*** | periplasmic-binding | c0679 | 15.118 | 0.001 | 0.933 | 0.824 | 20.626 | 0.000 | 359.603 | 0.000 | 1.527 | 0.733 | 864.874 | 0.000 |
| ***fepC*** | Ferric enterobactin | c0675 | 29.054 | 0.000 | 0.581 | 0.083 | 30.564 | 0.000 | 299.141 | 0.000 | 1.770 | 0.486 | 294.427 | 0.000 |
| ***fes*** | enterobactin/ferric | c0671 | 468.407 | 0.005 | 0.959 | 0.976 | 536.639 | 0.005 | 643.230 | 0.000 | 1.802 | 0.506 | 455.123 | 0.000 |
| ***fhuA*** | ferrichrome outer r | c0185 | 75.985 | 0.004 | 3.602 | 0.220 | 19.129 | 0.017 | 18.163 | 0.016 | 1.389 | 0.702 | 12.361 | 0.006 |
| ***fhuC*** | ATP-binding compo | c0186 | 49.845 | 0.000 | 1.397 | 0.519 | 47.309 | 0.000 | 48.090 | 0.001 | 1.339 | 0.630 | 46.869 | 0.001 |
| ***fhuF*** | ferric iron reductas | c5446 | 9.475 | 0.026 | 3.316 | 0.227 | 5.416 | 0.059 | 8.387 | 0.032 | 0.454 | 0.411 | 3.671 | 0.185 |
| ***fiu*** | predicted iron oute | c0890 | 22.787 | 0.033 | 1.995 | 0.332 | 4.786 | 0.066 | 83.811 | 0.001 | 0.039 | 0.010 | 118.702 | 0.000 |
| ***fldA*** | flavodoxin 1 | c0771 | 2.318 | 0.029 | 1.489 | 0.534 | 4.088 | 0.055 | 7.089 | 0.001 | 1.642 | 0.083 | 5.843 | 0.001 |
| ***frdB*** | fumarate reductase | c5241 | 0.388 | 0.000 | 0.982 | 0.799 | 1.012 | 0.896 | 0.294 | 0.004 | 1.112 | 0.448 | 1.179 | 0.414 |
| ***ftn*** | ferritin iron storage | c2321 | 0.362 | 0.000 | 0.354 | 0.003 | 0.231 | 0.000 | 0.276 | 0.006 | 0.865 | 0.599 | 0.255 | 0.006 |
| ***fumA*** | fumarate hydratase | c2004 | 0.259 | 0.003 | 0.693 | 0.228 | 0.963 | 0.847 | 0.130 | 0.000 | 1.058 | 0.511 | 0.847 | 0.119 |
| ***gpmA*** | phosphoglyceromu | c0831 | 10.691 | 0.000 | 1.297 | 0.004 | 10.087 | 0.000 | 8.828 | 0.000 | 0.992 | 0.909 | 7.721 | 0.000 |
| ***hybC*** | hydrogenase 2, larg | c3731 | 0.304 | 0.000 | 0.815 | 0.214 | 0.923 | 0.413 | 0.238 | 0.000 | 1.139 | 0.043 | 0.727 | 0.029 |
| ***hybO*** | hydrogenase 2, sm | c3734 | 0.035 | 0.009 | 0.709 | 0.092 | 0.856 | 0.130 | 0.038 | 0.000 | 1.091 | 0.717 | 0.726 | 0.209 |
| ***hypB*** | GTP hydrolase invo | c3287 | 0.204 | 0.001 | 0.862 | 0.475 | 0.900 | 0.542 | 0.095 | 0.000 | 0.854 | 0.092 | 0.924 | 0.455 |
| ***hypD*** | protein required fo | c3289 | 0.235 | 0.000 | 0.818 | 0.015 | 0.532 | 0.017 | 0.192 | 0.021 | 0.860 | 0.678 | 0.896 | 0.727 |

| | | | | | | | | | | | | | | |
|---|---|---|---|---|---|---|---|---|---|---|---|---|---|---|
| ***iscS*** | cysteine desulfuras | c3056 | 0.323 | 0.000 | 0.998 | 0.962 | 0.917 | 0.106 | 0.383 | 0.001 | 0.985 | 0.710 | 1.166 | 0.125 |
| ***iscU*** | FeS cluster assemb | c3055 | 0.390 | 0.000 | 0.983 | 0.629 | 0.942 | 0.343 | 0.483 | 0.000 | 1.026 | 0.754 | 1.301 | 0.016 |
| ***mdtA*** | multidrug efflux sy | c2600 | 3.500 | 0.000 | 1.043 | 0.754 | 4.536 | 0.000 | 2.390 | 0.004 | 1.271 | 0.127 | 3.357 | 0.006 |
| ***miaB*** | isopentenyl-adenos | c0747 | 0.436 | 0.000 | 1.023 | 0.364 | 0.913 | 0.012 | 0.402 | 0.000 | 1.031 | 0.390 | 0.965 | 0.542 |
| ***napC*** | nitrate reductase, | c2739 | 0.378 | 0.001 | 1.415 | 0.169 | 1.095 | 0.716 | 0.206 | 0.001 | 0.930 | 0.551 | 0.873 | 0.557 |
| ***narG*** | nitrate reductase 1 | c1685 | 0.484 | 0.001 | 1.525 | 0.012 | 1.038 | 0.835 | 0.129 | 0.000 | 1.719 | 0.213 | 1.271 | 0.001 |
| ***narH*** | nitrate reductase 1 | c1686 | 0.473 | 0.003 | 1.330 | 0.055 | 1.093 | 0.586 | 0.151 | 0.000 | 0.925 | 0.558 | 0.840 | 0.072 |
| ***nirD*** | nitrite reductase, N | c4142 | 0.470 | 0.027 | 1.268 | 0.276 | 0.935 | 0.724 | 0.335 | 0.000 | 1.042 | 0.771 | 1.245 | 0.328 |
| ***nrdE*** | ribonucleoside-dipl | c3228 | 514.119 | 0.000 | 2.011 | 0.359 | 454.223 | 0.000 | 150.041 | 0.006 | 1.098 | 0.864 | 210.998 | 0.000 |
| ***nrdF*** | ribonucleoside-dipl | c3229 | 18.797 | 0.020 | 0.664 | 0.633 | 15.998 | 0.032 | 8.458 | 0.021 | 0.530 | 0.584 | 10.838 | 0.014 |
| ***nrfA*** | nitrite reductase, f | c5066 | 0.062 | 0.041 | 1.352 | 0.692 | 2.105 | 0.224 | 0.002 | 0.000 | 0.937 | 0.728 | 0.749 | 0.009 |
| ***nuoA*** | NADH:ubiquinone | c2829 | 0.415 | 0.002 | 1.092 | 0.364 | 1.100 | 0.614 | 0.315 | 0.000 | 1.013 | 0.896 | 0.956 | 0.775 |
| ***nuoB*** | NADH:ubiquinone | c2828 | 0.319 | 0.000 | 1.086 | 0.278 | 1.073 | 0.416 | 0.273 | 0.000 | 0.900 | 0.198 | 0.927 | 0.340 |
| ***nuoC*** | NADH:ubiquinone | c2827 | 0.331 | 0.000 | 1.063 | 0.139 | 1.065 | 0.151 | 0.266 | 0.001 | 0.994 | 0.886 | 0.982 | 0.733 |
| ***nuoE*** | NADH:ubiquinone | c2826 | 0.331 | 0.000 | 1.036 | 0.214 | 1.024 | 0.630 | 0.333 | 0.001 | 1.134 | 0.256 | 1.045 | 0.673 |
| ***nuoG*** | NADH:ubiquinone | c2824 | 0.448 | 0.028 | 1.184 | 0.653 | 1.352 | 0.236 | 0.224 | 0.000 | 0.995 | 0.946 | 0.957 | 0.611 |
| ***nuoI*** | NADH:ubiquinone | c2822 | 0.327 | 0.001 | 1.007 | 0.935 | 0.927 | 0.517 | 0.308 | 0.000 | 0.989 | 0.894 | 0.773 | 0.200 |
| ***pflA*** | pyruvate formate l | c1038 | 0.459 | 0.000 | 0.997 | 0.953 | 1.068 | 0.177 | 0.298 | 0.000 | 1.016 | 0.900 | 1.066 | 0.754 |
| ***pqqL*** | predicted peptidas | c1923 | 90.193 | 0.000 | 0.724 | 0.429 | 42.005 | 0.001 | 712.904 | 0.000 | 0.775 | 0.721 | 103.926 | 0.000 |
| ***sdhA*** | succinate dehydrog | c0801 | 0.298 | 0.000 | 1.155 | 0.079 | 1.072 | 0.213 | 0.222 | 0.000 | 1.035 | 0.633 | 1.042 | 0.580 |
| ***sodA*** | superoxide dismuta | c4859 | 3.687 | 0.035 | 0.715 | 0.383 | 1.127 | 0.732 | 3.453 | 0.011 | 2.006 | 0.057 | 4.076 | 0.001 |
| ***sodB*** | superoxide dismuta | c2050 | 0.116 | 0.001 | 0.812 | 0.249 | 1.559 | 0.006 | 0.074 | 0.000 | 1.348 | 0.126 | 1.530 | 0.031 |
| ***sufC*** | transport protein a | c2077 | 3.185 | 0.007 | 1.303 | 0.366 | 4.585 | 0.002 | 2.308 | 0.000 | 1.163 | 0.412 | 0.324 | 0.250 |
| ***tonB*** | TonB protein | c1717 | 9.537 | 0.001 | 0.194 | 0.076 | 8.072 | 0.002 | 64.356 | 0.000 | 1.141 | 0.641 | 66.527 | 0.000 |
| ***ybdB*** | conserved protein | c0684 | 361.446 | 0.000 | 0.690 | 0.588 | 489.785 | 0.000 | 99.807 | 0.001 | 0.666 | 0.426 | 96.672 | 0.001 |
| ***ybdZ*** | conserved protein | c0672 | 57.364 | 0.000 | 1.967 | 0.176 | 46.579 | 0.001 | 109.411 | 0.001 | 1.140 | 0.843 | 106.098 | 0.001 |
| ***ybiX*** | conserved protein | c0889 | 2.606 | 0.001 | 0.925 | 0.451 | 1.927 | 0.014 | 11.111 | 0.000 | 0.526 | 0.382 | 12.615 | 0.000 |
| ***yddA*** | membrane and AT | c1925 | 297.931 | 0.000 | 3.247 | 0.313 | 90.548 | 0.001 | 85.570 | 0.000 | 1.842 | 0.180 | 20.136 | 0.003 |
| ***yddB*** | predicted porin pro | c1924 | 2613.127 | 0.000 | 1.345 | 0.837 | 213.039 | 0.001 | 35.034 | 0.012 | 0.635 | 0.760 | 40.961 | 0.006 |
| ***ydeN*** | conserved protein | c1927 | 0.225 | 0.047 | 1.259 | 0.512 | 1.416 | 0.395 | 0.294 | 0.002 | 0.737 | 0.072 | 0.978 | 0.921 |
| ***ydiE*** | conserved protein | c2101 | 17.926 | 0.000 | 2.379 | 0.067 | 30.441 | 0.000 | 37.872 | 0.015 | 0.791 | 0.817 | 39.780 | 0.014 |
| ***ydiI*** | esterase | c2081 | 0.418 | 0.000 | 1.120 | 0.184 | 1.127 | 0.055 | 0.344 | 0.000 | 0.901 | 0.121 | 0.733 | 0.088 |
| ***ydiJ*** | predicted FAD-link | c2082 | 0.193 | 0.006 | 0.678 | 0.223 | 0.325 | 0.154 | 0.204 | 0.000 | 1.038 | 0.513 | 1.072 | 0.548 |
| ***ygiQ*** | conserved protein | c3749 | 0.173 | 0.000 | 0.989 | 0.891 | 0.770 | 0.020 | 0.326 | 0.007 | 0.793 | 0.160 | 0.876 | 0.532 |
| ***yhgG*** | predicted DNA-bin | c4187 | 15.565 | 0.001 | 0.468 | 0.282 | 20.061 | 0.002 | 31.341 | 0.000 | 1.535 | 0.640 | 26.131 | 0.000 |
| ***yjiM*** | predicted 2-hydrox | c5418 | 0.182 | 0.011 | 1.094 | 0.802 | 0.928 | 0.849 | 0.332 | 0.002 | 0.909 | 0.597 | 0.821 | 0.453 |
| ***yncE*** | conserved protein | c1877 | 204.629 | 0.000 | 0.699 | 0.503 | 7.041 | 0.008 | 232.716 | 0.000 | 0.714 | 0.272 | 28.021 | 0.000 |
| ***ynfE*** | oxidoreductase sub | c1977 | 0.307 | 0.022 | 0.956 | 0.932 | 1.366 | 0.428 | 0.013 | 0.000 | 1.233 | 0.325 | 1.056 | 0.804 |
| ***ynfF*** | oxidoreductase sub | c1978 | 0.042 | 0.000 | 0.632 | 0.070 | 0.785 | 0.294 | 0.013 | 0.000 | 1.135 | 0.009 | ND | ND |
| ***ynfG*** | oxidoreductase, Fe | c1979 | 0.066 | 0.000 | 0.818 | 0.127 | 1.033 | 0.688 | 0.039 | 0.000 | 0.946 | 0.649 | 0.961 | 0.829 |
| ***yohM*** | membrane protein | c2633 | 16.583 | 0.000 | 1.494 | 0.521 | 25.059 | 0.000 | 47.199 | 0.008 | 6.970 | 0.170 | 65.467 | 0.005 |
| ***yojI*** | membrane and AT | c2752 | 17.686 | 0.000 | 0.721 | 0.172 | 12.989 | 0.000 | 33.83478 | 0.000 | 2.045 | 0.025 | 19.482 | 0.000 |
| ***yqjH*** | predicted sideroph | c3823 | 2.487 | 0.030 | 0.436 | 0.056 | 4.566 | 0.006 | 11.856 | 0.001 | 5.931 | 0.003 | 32.668 | 0.000 |
| ***zntA*** | zinc, cobalt and lea | c4262 | 4.033 | 0.000 | 1.032 | 0.691 | 3.045 | 0.021 | 7.902 | 0.023 | 1.122 | 0.109 | 2.990 | 0.000 |

*CFT073 specific proteins that are differentially abundant in $\Delta$fur*

| Gene[2] | Protein product[2] | c-number[2] | CFT073 Proteomics fold change[3] | | | | | |
|---|---|---|---|---|---|---|---|---|
| | | | $fur^-$/wt | p-value | $ryhB^-$/wt | p-value | $fur^- ryhB^-$/wt | p-value |
| ***chuA*** | outer membrane h | c4308 | 9760.412 | 0.000 | 1.515 | 0.776 | 5209.093 | 0.000 |
| ***chuS*** | putative heme/her | c4307 | 15.837 | 0.000 | 0.266 | 0.005 | 32.782 | 0.000 |
| ***chuT*** | putative periplasm | c4313 | 6862.837 | 0.001 | 4.698 | 0.335 | 7789.146 | 0.001 |
| ***chuW*** | putative oxygen inc | c4314 | 10460.765 | 0.000 | 20.673 | 0.043 | 12559.608 | 0.000 |
| ***chuX*** | orf; hypothetical pr | c4315 | 194.875 | 0.000 | 1.073 | 0.831 | 390.095 | 0.000 |
| ***chuY*** | orf; hypothetical pr | c4316 | 428.876 | 0.000 | 1.177 | 0.792 | 1205.477 | 0.000 |
| ***hma*** | haem receptor | c2482 | 3834.065 | 0.001 | 3.831 | 0.495 | 2082.252 | 0.001 |
| ***iha*** | catecholate siderop | c3610 | 452.572 | 0.000 | 0.859 | 0.798 | 273.728 | 0.000 |
| ***iroB*** | putative glucosyltra | c1254 | 25761.933 | 0.000 | 1.629 | 0.217 | 21235.987 | 0.000 |
| ***iroC*** | ATP binding casset | c1253 | 62.085 | 0.000 | 1.020 | 0.948 | 47.458 | 0.000 |
| ***iroD*** | ferric enterochelin | c1252 | 107.271 | 0.000 | 0.723 | 0.294 | 116.989 | 0.000 |
| ***iroE*** | IroE protein | c1251 | 82.967 | 0.001 | 0.256 | 0.183 | 141.087 | 0.001 |
| ***iroN*** | siderophore recept | c1250 | 867.363 | 0.000 | 1.241 | 0.140 | 3.565 | 0.014 |
| ***iucA*** | aerobactin synthet | c3627 | 189.961 | 0.001 | 1.886 | 0.349 | 204.201 | 0.000 |
| ***iucB*** | iucB protein | c3626 | 3369.844 | 0.000 | 1.963 | 0.043 | 2998.155 | 0.000 |
| ***iucC*** | iucC protein | c3625 | 942.647 | 0.000 | 0.629 | 0.588 | 789.994 | 0.000 |
| ***iucD*** | iucD protein | c3624 | 983.543 | 0.000 | 1.235 | 0.867 | 321.434 | 0.000 |
| ***iutA*** | iutA protein | c3623 | 171.439 | 0.000 | 0.914 | 0.708 | 63.876 | 0.000 |
| ***mchC*** | mchC protein | c1229 | 1301.817 | 0.000 | 0.173 | 0.135 | 1652.994 | 0.000 |
| ***mchD*** | mchD protein | c1230 | 61.195 | 0.000 | 5.440 | 0.083 | 144.310 | 0.000 |
| ***mchE*** | Microcin H47 secre | c1231 | 8.886 | 0.044 | 1.242 | 0.798 | 53.433 | 0.006 |
| ***mchF*** | Probable microcin | c1232 | 43.127 | 0.042 | 2.876 | 0.454 | 286.329 | 0.010 |
| ***papA*** | papA protein | c3592 | 0.185 | 0.033 | 0.813 | 0.753 | 0.724 | 0.589 |
| ***papA_2*** | papA protein | c5188 | 0.054 | 0.044 | 0.692 | 0.548 | 0.460 | 0.150 |
| ***papG*** | papG protein | c3583 | 0.238 | 0.033 | 0.773 | 0.634 | 0.630 | 0.545 |
| ***papJ_2*** | papJ protein | c5184 | 0.177 | 0.037 | 1.121 | 0.757 | 0.886 | 0.738 |
| ***sitB*** | sitB protein | c1599 | 109.072 | 0.002 | 0.710 | 0.657 | 46.879 | 0.002 |
| ***ybiJ*** | predicted protein | c0886 | 2.510 | 0.027 | 0.658 | 0.153 | 0.853 | 0.591 |
| ***ybtA*** | Putataive AraC type | c2423 | 56.528 | 0.001 | 0.063 | 0.015 | 46.771 | 0.000 |
| ***ybtS*** | Putative anthranila | c2419 | 21.629 | 0.003 | 1.603 | 0.334 | 6.245 | 0.009 |
| ***yeaE*** | predicted oxidored | c2186 | 0.361 | 0.001 | 0.897 | 0.245 | 0.929 | 0.377 |
| ***c0292*** | Hypothetical protei | c0292 | 0.480 | 0.013 | 0.886 | 0.598 | 1.016 | 0.928 |
| ***c0336*** | PTS system, manni | c0336 | 0.224 | 0.011 | 0.790 | 0.574 | 0.966 | 0.925 |
| ***c0933*** | Hypothetical protei | c0933 | 0.267 | 0.044 | 1.317 | 0.552 | 0.811 | 0.658 |
| ***c0946*** | Hypothetical protei | c0946 | 0.132 | 0.026 | 1.278 | 0.579 | 1.776 | 0.200 |
| ***c1220*** | Phospho-2-dehydrc | c1220 | 764.308 | 0.000 | 0.790 | 0.568 | 712.658 | 0.000 |
| ***c1536*** | putative recombina | c1536 | 2.614 | 0.004 | 1.353 | 0.192 | 2.913 | 0.001 |
| ***c1583*** | putative tail compc | c1583 | 3.839 | 0.004 | 1.977 | 0.016 | 3.504 | 0.007 |
| ***c1843*** | Glyceraldehyde 3-p | c1843 | 2.951 | 0.003 | 0.074 | 0.010 | 0.220 | 0.024 |

| | | | | | | | | |
|---|---|---|---|---|---|---|---|---|
| ***c2411*** | DNA-binding protei | c2411 | 2.100 | 0.025 | 1.660 | 0.304 | 2.269 | 0.016 |
| ***c2454*** | C-terminal fragmer | c2454 | 2.498 | 0.024 | 0.858 | 0.621 | 1.490 | 0.071 |
| ***c2457*** | Putative amidase | c2457 | 6.167 | 0.016 | 0.663 | 0.520 | 4.910 | 0.027 |
| ***c2458*** | Putative nonriboso | c2458 | 2.770 | 0.021 | 0.855 | 0.629 | 1.850 | 0.079 |
| ***c2460*** | Putative polyketide | c2460 | 4.886 | 0.016 | 0.909 | 0.874 | 4.016 | 0.018 |
| ***c2461*** | predicted nonribos | c2461 | 4.433 | 0.005 | 0.886 | 0.806 | 3.121 | 0.021 |
| ***c2463*** | Putative transacyla | c2463 | 6.022 | 0.025 | 1.425 | 0.543 | 3.902 | 0.067 |
| ***c2464*** | Putative acyl-coa d | c2464 | 4.774 | 0.016 | 0.958 | 0.941 | 3.117 | 0.029 |
| ***c2468*** | Putative polyketide | c2468 | 7.391 | 0.001 | 1.539 | 0.201 | 4.153 | 0.001 |
| ***c2469*** | C-terminal fragmer | c2469 | 8.420 | 0.032 | 0.623 | 0.598 | 4.515 | 0.075 |
| ***c2470*** | N-terminal fragme | c2470 | 4.716 | 0.024 | 0.831 | 0.830 | 4.358 | 0.022 |
| ***c3568*** | Hypothetical protei | c3568 | 0.254 | 0.046 | 0.999 | 0.999 | 1.206 | 0.735 |
| ***c3639*** | N-acetylneuramina | c3639 | 0.361 | 0.002 | 1.761 | 0.028 | 0.795 | 0.378 |
| ***c3736*** | putative enzyme | c3736 | 3.076 | 0.012 | 0.909 | 0.499 | 0.993 | 0.979 |
| ***c3770*** | orf; hypothetical pr | c3770 | 15.384 | 0.000 | 1.390 | 0.207 | 8.308 | 0.000 |
| ***c3771*** | putative iron comp | c3771 | 8.320 | 0.003 | 1.289 | 0.336 | 6.820 | 0.001 |
| ***c3774*** | Ferric enterobactin | c3774 | 65.936 | 0.000 | 0.546 | 0.080 | 28.725 | 0.000 |
| ***c4318*** | putative ATP-bindi | c4318 | 32.734 | 0.011 | 0.059 | 0.033 | 72.187 | 0.005 |
| ***c5036*** | Succinyl-CoA synth | c5036 | 2.584 | 0.005 | 0.718 | 0.208 | 1.600 | 0.522 |
| ***c5174*** | Putative iron-regul | c5174 | 185.197 | 0.000 | 0.464 | 0.281 | 170.835 | 0.000 |
| ***c5382*** | N-terminal fragme | c5382 | 0.448 | 0.000 | 0.760 | 0.182 | 1.123 | 0.552 |
| ***c5424*** | putative restriction | c5424 | 0.282 | 0.037 | 2.197 | 0.109 | 0.432 | 0.117 |

*Orthologous proteins that are differentially abundant in CFT073 but not in MG1655 $\Delta fur$*

| **Gene**[2] | **Protein product**[2] | **c-number**[2] | **CFT073 Proteomics fold change**[3] | | | | | | **MG1655 Proteomics fold change**[4] | | | | | |
|---|---|---|---|---|---|---|---|---|---|---|---|---|---|---|
| | | | **fur$^-$/wt** | **p-value** | **ryhB$^-$/wt** | **p-value** | **fur$^-$ryhB$^-$/wt** | **p-value** | **fur$^-$/wt** | **p-value** | **ryhB$^-$/wt** | **p-value** | **fur$^-$ryhB$^-$/wt** | **p-value** |
| ***adhP*** | alcohol dehydroger | c1911 | 3.370 | 0.009 | 1.790 | 0.014 | 2.676 | 0.040 | 1.356 | 0.422 | 0.822 | 0.342 | 0.926 | 0.750 |
| ***amtB*** | ammonium transp | c0570 | 3.429 | 0.028 | 0.550 | 0.225 | 2.235 | 0.057 | ND | ND | ND | ND | ND | ND |
| ***argT*** | periplasmic-bindin | c5772 | 0.039 | 0.008 | 0.277 | 0.326 | 0.076 | 0.008 | 0.649 | 0.003 | 0.878 | 0.250 | ND | ND |
| ***azoR*** | NADH-azoreductas | c1839 | 0.499 | 0.000 | 0.998 | 0.991 | 0.881 | 0.184 | 0.783 | 0.563 | 0.915 | 0.777 | 0.529 | 0.423 |
| ***betA*** | choline dehydroger | c0431 | 19.670 | 0.030 | 4.266 | 0.450 | 5.288 | 0.140 | ND | ND | ND | ND | ND | ND |
| ***cadA*** | lysine decarboxylas | c5140 | 24.171 | 0.013 | 16.841 | 0.005 | 11.072 | 0.006 | 0.900 | 0.192 | 0.119 | 0.006 | 0.343 | 0.273 |
| ***cchA*** | predicted carboxys | c2982 | 2.353 | 0.011 | 1.273 | 0.304 | 1.066 | 0.751 | 1.766 | 0.430 | 0.589 | 0.483 | 1.545 | 0.701 |
| ***chbG*** | conserved protein | c2132 | 2.270 | 0.014 | 0.975 | 0.880 | 1.343 | 0.175 | 1.729 | 0.013 | 0.811 | 0.183 | 0.955 | 0.833 |
| ***citE*** | citrate lyase, citryl- | c0706 | 2.989 | 0.023 | 0.514 | 0.375 | 2.496 | 0.046 | 1.942 | 0.663 | 0.852 | 0.924 | 0.151 | 0.058 |
| ***cybC*** | Soluble cytochrome | c5335 | 0.114 | 0.000 | 0.780 | 0.363 | 1.058 | 0.564 | 1.140 | 0.915 | 0.290 | 0.190 | 1.462 | 0.749 |
| ***cysW*** | membrane compo | c2957 | 0.467 | 0.016 | 0.685 | 0.353 | 0.244 | 0.004 | 1.169 | 0.626 | 1.158 | 0.564 | 0.780 | 0.290 |
| ***deaD*** | ATP-dependent RN | c3916 | 0.283 | 0.017 | 0.786 | 0.527 | 0.627 | 0.215 | 0.528 | 0.002 | 0.857 | 0.403 | 0.761 | 0.034 |
| ***dld*** | D-lactate dehydrog | c2664 | 2.126 | 0.016 | 0.740 | 0.077 | 1.276 | 0.036 | 1.474 | 0.001 | 1.025 | 0.753 | 1.306 | 0.006 |
| ***dxs*** | 1-deoxyxylulose-5- | c0531 | 0.424 | 0.011 | 0.876 | 0.552 | 1.101 | 0.853 | 0.564 | 0.000 | 0.954 | 0.485 | 1.136 | 0.382 |
| ***ecnB*** | entericidin B meml | c5235 | 2.102 | 0.020 | 0.882 | 0.783 | 0.637 | 0.074 | 1.247 | 0.715 | 1.268 | 0.705 | 0.650 | 0.372 |
| ***emrA*** | multidrug efflux sy | c3238 | 0.450 | 0.001 | 0.860 | 0.356 | 0.989 | 0.891 | 0.715 | 0.042 | 1.069 | 0.528 | 1.035 | 0.156 |
| ***eutB*** | ethanolamine amn | c2975 | 2.002 | 0.016 | 1.785 | 0.296 | 1.252 | 0.306 | 1.228 | 0.020 | 1.022 | 0.872 | 0.700 | 0.372 |

| | | | | | | | | | | | | | | |
|---|---|---|---|---|---|---|---|---|---|---|---|---|---|---|
| ***fadL*** | long-chain fatty aci | c2889 | 0.312 | 0.001 | 0.198 | 0.004 | 0.077 | 0.012 | 0.987 | 0.978 | 1.459 | 0.486 | 2.041 | 0.168 |
| ***fis*** | global DNA-binding | c4027 | 0.473 | 0.000 | 0.755 | 0.037 | 0.538 | 0.001 | 0.774 | 0.068 | 1.077 | 0.459 | 0.855 | 0.511 |
| ***fumB*** | anaerobic class I fu | c5127 | 0.265 | 0.001 | 0.724 | 0.403 | 1.059 | 0.718 | 1.002 | 0.528 | 0.905 | 0.316 | 0.684 | 0.010 |
| ***gadC*** | predicted glutamat | c1921 | 2.014 | 0.004 | 0.702 | 0.078 | 1.135 | 0.489 | 1.210 | 0.769 | 1.130 | 0.386 | 1.043 | 0.866 |
| ***glnK*** | nitrogen assimilati | c0568 | 3.662 | 0.006 | 0.668 | 0.349 | 2.588 | 0.017 | 1.121 | 0.596 | 0.854 | 0.071 | 1.249 | 0.169 |
| ***gltP*** | glutamate/asparta | c5074 | 0.432 | 0.007 | 1.897 | 0.142 | 1.435 | 0.177 | 0.580 | 0.194 | 0.850 | 0.644 | 1.322 | 0.020 |
| ***grxD*** | monothiol glutarec | c2048 | 0.475 | 0.002 | 1.168 | 0.128 | 1.061 | 0.416 | 0.506 | 0.001 | 1.239 | 0.057 | 1.257 | 0.045 |
| ***hscA*** | DnaK-like molecula | c3051 | 0.446 | 0.000 | 0.972 | 0.477 | 0.918 | 0.086 | 0.774 | 0.120 | 1.082 | 0.333 | 1.163 | 0.188 |
| ***hybA*** | hydrogenase 2 4Fe | c3733 | 0.042 | 0.000 | 0.721 | 0.313 | 0.886 | 0.397 | 1.045 | 0.128 | 1.146 | 0.371 | 0.707 | 0.021 |
| ***hybD*** | predicted maturati | c3730 | 0.362 | 0.007 | 0.589 | 0.078 | 0.441 | 0.041 | 0.591 | 0.149 | 1.106 | 0.364 | 1.641 | 0.263 |
| ***hycB*** | hydrogenase 3, Fe- | c3284 | 0.174 | 0.007 | 0.272 | 0.017 | 0.276 | 0.043 | 1.249 | 0.112 | 1.198 | 0.591 | 0.716 | 0.029 |
| ***hycG*** | hydrogenase 3 and | c3279 | 0.054 | 0.004 | 0.225 | 0.012 | 0.136 | 0.016 | 1.856 | 0.478 | 1.720 | 0.549 | 2.795 | 0.317 |
| ***idi*** | isopentenyl diphos | c3467 | 2.366 | 0.013 | 1.011 | 0.947 | 1.660 | 0.028 | 1.068 | 0.764 | 0.988 | 0.932 | 0.955 | 0.736 |
| ***ilvB*** | acetolactate syntha | c4596 | 0.353 | 0.001 | 1.107 | 0.495 | 1.256 | 0.061 | 0.542 | 0.010 | 0.948 | 0.262 | 1.025 | 0.887 |
| ***ilvN*** | acetolactate syntha | c4595 | 0.382 | 0.002 | 1.300 | 0.009 | 1.287 | 0.023 | 0.660 | 0.006 | 1.053 | 0.355 | 0.977 | 0.773 |
| ***kpsF*** | predicted sugar ph | c3686 | 0.448 | 0.007 | 1.005 | 0.984 | 0.747 | 0.111 | 1.191 | 0.874 | 0.317 | 0.438 | 0.485 | 0.610 |
| ***lysP*** | lysine transporter | c2691 | 0.293 | 0.003 | 1.150 | 0.538 | 1.119 | 0.683 | 1.196 | 0.339 | 1.072 | 0.587 | 0.991 | 0.964 |
| ***maa*** | maltose O-acetyltr | c0577 | 0.351 | 0.000 | 0.941 | 0.839 | 1.022 | 0.797 | 0.681 | 0.008 | 1.131 | 0.313 | 0.935 | 0.151 |
| ***malE*** | periplasmic-bindin | c5004 | 0.483 | 0.037 | 1.215 | 0.418 | 1.129 | 0.577 | 1.251 | 0.484 | 1.195 | 0.225 | 1.757 | 0.091 |
| ***mdtB*** | multidrug efflux sy | c2601 | 3.009 | 0.000 | 1.004 | 0.980 | 4.160 | 0.000 | 1.347 | 0.113 | 0.744 | 0.083 | 1.374 | 0.238 |
| ***mdtC*** | multidrug efflux sy | c2602 | 4.167 | 0.004 | 1.249 | 0.435 | 8.389 | 0.002 | 0.738 | 0.675 | 0.532 | 0.439 | 0.665 | 0.578 |
| ***mdtE*** | multidrug resistanc | c4324 | 3.075 | 0.000 | 0.949 | 0.394 | 1.582 | 0.000 | 0.666 | 0.018 | 1.110 | 0.264 | 0.579 | 0.014 |
| ***mdtF*** | multidrug transpor | c4325 | 3.729 | 0.005 | 0.729 | 0.016 | 1.501 | 0.013 | 1.188 | 0.044 | 0.961 | 0.558 | 1.075 | 0.486 |
| ***moaB*** | molybdopterin bios | c0863 | 2.263 | 0.007 | 1.355 | 0.170 | 1.785 | 0.026 | 1.139 | 0.278 | 0.924 | 0.436 | 1.007 | 0.942 |
| ***nac*** | DNA-binding transc | c2446 | 3.609 | 0.008 | 0.501 | 0.370 | 1.998 | 0.065 | ND | ND | ND | ND | ND | ND |
| ***narK*** | nitrate/nitrite tran | c1684 | 5.508 | 0.007 | 1.849 | 0.284 | 0.621 | 0.182 | 1.201 | 0.602 | 0.646 | 0.235 | 0.662 | 0.254 |
| ***narP*** | DNA-binding respo | c2730 | 0.343 | 0.002 | 0.809 | 0.312 | 1.204 | 0.206 | 0.515 | 0.074 | 0.858 | 0.388 | 1.260 | 0.113 |
| ***narZ*** | nitrate reductase 2 | c1900 | 0.098 | 0.000 | 0.509 | 0.025 | 0.412 | 0.006 | 0.567 | 0.519 | 1.031 | 0.939 | 0.291 | 0.016 |
| ***oppA*** | periplasmic-bindin | c1707 | 0.401 | 0.008 | 1.003 | 0.990 | 0.983 | 0.926 | 0.900 | 0.011 | 1.038 | 0.399 | 1.072 | 0.078 |
| ***oppB*** | oligopeptide transp | c1708 | 0.165 | 0.000 | 0.610 | 0.516 | 0.576 | 0.024 | 0.529 | 0.011 | 1.139 | 0.412 | 0.850 | 0.310 |
| ***oppC*** | membrane compor | c1709 | 0.281 | 0.000 | 0.955 | 0.336 | 0.617 | 0.000 | 1.481 | 0.187 | 1.071 | 0.038 | 0.984 | 0.834 |
| ***oppD*** | oligopeptide transp | c1710 | 0.293 | 0.000 | 0.992 | 0.949 | 0.719 | 0.008 | 0.509 | 0.001 | 1.065 | 0.379 | 0.951 | 0.602 |
| ***oppF*** | ATP-binding subuni | c1711 | 0.323 | 0.000 | 1.028 | 0.804 | 0.738 | 0.002 | 0.747 | 0.767 | 1.062 | 0.095 | 0.999 | 0.995 |
| ***pheS*** | phenylalanine tRN/ | c2112 | 0.478 | 0.004 | 1.098 | 0.464 | 0.642 | 0.023 | 0.973 | 0.705 | 1.086 | 0.311 | 0.984 | 0.861 |
| ***pitA*** | phosphate transpo | c4291 | 0.320 | 0.000 | 0.681 | 0.002 | 0.200 | 0.001 | 0.724 | 0.133 | 1.065 | 0.560 | 1.029 | 0.712 |
| ***potF*** | putrescine transpo | c0987 | 2.041 | 0.010 | 1.233 | 0.187 | 1.524 | 0.046 | 0.724 | 0.256 | 0.938 | 0.389 | 0.981 | 0.794 |
| ***potG*** | putrescine transpo | c0988 | 2.255 | 0.002 | 1.362 | 0.032 | 1.661 | 0.019 | 1.215 | 0.217 | 0.884 | 0.070 | 1.077 | 0.539 |
| ***poxB*** | pyruvate dehydrog | c1004 | 3.064 | 0.010 | 1.354 | 0.158 | 2.114 | 0.050 | 1.141 | 0.051 | 1.067 | 0.202 | 1.060 | 0.577 |
| ***proC*** | pyrroline-5-carboxy | c0493 | 0.496 | 0.023 | 0.294 | 0.120 | 0.413 | 0.079 | 0.603 | 0.004 | 1.200 | 0.074 | ND | ND |
| ***rbbA*** | ribosome-associate | c4286 | 2.353 | 0.038 | 2.221 | 0.045 | 2.162 | 0.079 | 1.263 | 0.159 | 1.074 | 0.299 | 1.047 | 0.776 |
| ***rpoH*** | RNA polymerase, s | c4254 | 0.322 | 0.005 | 1.164 | 0.691 | 0.365 | 0.013 | 1.283 | 0.619 | 1.420 | 0.390 | 4.695 | 0.020 |
| ***rpoS*** | RNA polymerase, s | c5629 | 3.855 | 0.001 | 1.313 | 0.278 | 1.656 | 0.042 | 0.765 | 0.190 | 0.767 | 0.517 | 0.405 | 0.032 |
| ***rsxC*** | inner membrane ir | c2021 | 0.372 | 0.004 | 0.930 | 0.639 | 0.751 | 0.222 | 0.805 | 0.087 | 1.444 | 0.623 | 2.427 | 0.274 |
| ***sdaC*** | predicted serine tra | c3364 | 0.294 | 0.009 | 1.218 | 0.483 | 1.099 | 0.648 | 0.866 | 0.466 | 1.174 | 0.271 | 1.130 | 0.340 |

| | | | | | | | | | | | | | | |
|---|---|---|---|---|---|---|---|---|---|---|---|---|---|---|
| ***sdhD*** | succinate dehydrog | c0800 | 0.098 | 0.021 | 1.593 | 0.410 | 0.708 | 0.543 | 0.542 | 0.394 | 3.239 | 0.102 | 0.950 | 0.954 |
| ***sra*** | 30S ribosomal subu | c1913 | 2.401 | 0.003 | 0.962 | 0.814 | 1.432 | 0.038 | 0.821 | 0.113 | 0.929 | 0.487 | 0.722 | 0.001 |
| ***ssuD*** | alkanesulfonate m | c1078 | 0.304 | 0.015 | 0.893 | 0.737 | 0.617 | 0.163 | 1.169 | 0.636 | 1.155 | 0.839 | 1.999 | 0.173 |
| ***sufD*** | component of SufB | c2076 | 2.659 | 0.029 | 1.596 | 0.413 | 5.715 | 0.002 | 1.788 | 0.002 | 0.860 | 0.920 | 0.246 | 0.430 |
| ***sufS*** | selenocysteine lyas | c2075 | 2.825 | 0.022 | 1.495 | 0.223 | 2.463 | 0.081 | 1.878 | 0.001 | 0.947 | 0.614 | 2.312 | 0.001 |
| ***tktB*** | transketolase 2, thi | c2990 | 2.176 | 0.000 | 1.059 | 0.531 | 1.284 | 0.000 | 1.240 | 0.014 | 1.103 | 0.175 | 0.981 | 0.672 |
| ***ucpA*** | predicted oxidored | c2960 | 4.080 | 0.018 | 2.321 | 0.169 | 1.437 | 0.534 | 0.803 | 0.291 | 1.145 | 0.093 | 1.140 | 0.103 |
| ***ugpB*** | periplasmic-bindin | c4242 | 0.435 | 0.004 | 1.062 | 0.541 | 0.685 | 0.234 | 0.795 | 0.164 | 1.194 | 0.226 | 1.317 | 0.093 |
| ***waaL*** | lipid A-core, surfac | c4448 | 0.350 | 0.004 | 0.871 | 0.597 | 0.996 | 0.975 | ND | ND | ND | ND | ND | ND |
| ***ybaS*** | predicted glutamin | c0605 | 2.032 | 0.047 | 1.823 | 0.051 | 1.096 | 0.814 | 0.804 | 0.149 | 0.885 | 0.226 | 0.592 | 0.003 |
| ***ybhB*** | predicted kinase in | c0850 | 2.028 | 0.014 | 0.413 | 0.069 | 0.487 | 0.021 | 1.198 | 0.268 | 1.083 | 0.589 | ND | ND |
| ***ybhO*** | cardiolipin synthas | c0872 | 2.763 | 0.016 | 3.567 | 0.146 | 1.964 | 0.221 | 1.169 | 0.764 | 0.354 | 0.123 | 1.127 | 0.836 |
| ***ycgB*** | conserved protein | c1637 | 2.581 | 0.000 | 0.947 | 0.773 | 1.427 | 0.006 | 0.513 | 0.023 | 1.002 | 0.994 | 0.712 | 0.294 |
| ***yciE*** | conserved protein | c1723 | 2.054 | 0.009 | 0.926 | 0.747 | 1.253 | 0.192 | 1.430 | 0.006 | 1.134 | 0.426 | 1.223 | 0.279 |
| ***ycjY*** | predicted hydrolase | c1801 | 2.600 | 0.007 | 1.036 | 0.905 | 1.633 | 0.036 | 1.185 | 0.477 | 0.350 | 0.009 | 0.955 | 0.938 |
| ***ydfK*** | Hypothetical protei | c3145 | 0.265 | 0.009 | 0.630 | 0.156 | 0.553 | 0.115 | 0.517 | 0.058 | 0.869 | 0.707 | 2.533 | 0.011 |
| ***ydhV*** | predicted oxidored | c2067 | 0.027 | 0.011 | 1.113 | 0.817 | 0.068 | 0.061 | 0.282 | 0.066 | 0.875 | 0.594 | 0.205 | 0.021 |
| ***ydjN*** | predicted transport | c2128 | 0.282 | 0.000 | 1.159 | 0.103 | 0.201 | 0.000 | 0.979 | 0.802 | 2.143 | 0.740 | 3.835 | 0.552 |
| ***yeaG*** | conserved protein v | c2188 | 2.457 | 0.000 | 0.945 | 0.445 | 1.697 | 0.001 | 0.793 | 0.018 | 1.030 | 0.464 | 1.017 | 0.859 |
| ***yeaH*** | conserved protein | c2189 | 3.852 | 0.002 | 1.016 | 0.936 | 2.163 | 0.013 | 0.648 | 0.046 | 1.062 | 0.732 | 0.879 | 0.326 |
| ***yfaZ*** | predicted outer me | c2792 | 0.248 | 0.005 | 0.589 | 0.058 | 0.260 | 0.006 | 1.257 | 0.587 | 0.605 | 0.654 | 0.134 | 0.330 |
| ***yfdZ*** | prediected aminotr | c2916 | 11.842 | 0.035 | 4.660 | 0.057 | 0.888 | 0.735 | 1.318 | 0.009 | 1.177 | 0.148 | 1.278 | 0.073 |
| ***ygjG*** | putrescine:2-oxoglu | c3828 | 4.114 | 0.046 | 0.453 | 0.292 | 3.419 | 0.054 | 1.327 | 0.591 | 1.290 | 0.662 | 1.186 | 0.698 |
| ***yhdA*** | conserved inner me | c4008 | 2.554 | 0.047 | 0.919 | 0.672 | 1.178 | 0.337 | 1.200 | 0.142 | 1.109 | 0.409 | 1.109 | 0.276 |
| ***yheT*** | predicted hydrolase | c4128 | 0.374 | 0.010 | 0.602 | 0.054 | 0.299 | 0.003 | 0.776 | 0.718 | 2.030 | 0.338 | 2.091 | 0.338 |
| ***yhfG*** | predicted protein | c4137 | 2.077 | 0.001 | 1.151 | 0.320 | 1.047 | 0.853 | 1.193 | 0.259 | 0.844 | 0.261 | 0.797 | 0.171 |
| ***yiiT*** | stress-induced prot | c4875 | 2.159 | 0.003 | 1.221 | 0.306 | 1.340 | 0.285 | 1.454 | 0.013 | 1.056 | 0.742 | 1.312 | 0.171 |
| ***yjgB*** | predicted alcohol d | c5370 | 0.438 | 0.037 | 1.004 | 0.988 | 0.707 | 0.187 | 1.492 | 0.177 | 1.127 | 0.646 | 1.126 | 0.652 |
| ***yjiL*** | predicted ATPase, a | c5417 | 0.271 | 0.011 | 0.791 | 0.531 | 0.476 | 0.085 | 1.003 | 0.115 | 1.073 | 0.822 | 1.389 | 0.368 |
| ***ynjE*** | predicted thiosulfa | c2158 | 0.473 | 0.000 | 0.773 | 0.070 | 0.551 | 0.002 | 0.803 | 0.022 | 1.151 | 0.136 | 1.009 | 0.971 |

[1]CFT073 proteins that are differentially expressed in Δ*fur* strains of CFT073 and MG1655.

[2]Gene functions, predicted operons, gene names, protein annotations and c numbers are obtained from Ecocyc.

[3]Fold change in protein abundance of CFT073 Δ*fur* (WAM5491), Δ*ryhB* (WAM5497) or Δ*fur* Δ*ryhB* (WAM5499) compared to wild type (WAM4505) obtained from our proteomics dataset.

[4]Fold change in protein abundance of MG1655 Δ*fur* (PK9427), Δ*ryhB* (PK10474) orf Δ*fur* Δ*ryhB* (PK10475) compared to wild type, obtained from our proteomics dataset.
A 2-fold change in protein levels (up-regulation or down-regulation) in Δ*fur* as compared to wild type was selected as the cut-off for both CFT073 and MG1655 proteomics data.
ND=not detected in our dataset.
