# Supplementary material for: Tailoring a Global Iron Regulon to a Uropathogen
Source: mBio. 2020 Mar 24;11(2):e00351-20. doi: 10.1128/mBio.00351-20 (PMC7157518; doi:10.1128/mBio.00351-20)
Supplement: TABLE S5 [file mBio.00351-20-st005.pdf]

## Table S5A: CFT073 Fur Chip-seq peaks[1]

| Gene[2] | Protein product[2] | c-number[2] | ChIP-seq[3] | | Present in |
|---|---|---|---|---|---|
| | | | Peak Max Location | Peak Summit | pathogenicity islands[4] |
| ***acnA*** | aconitate hydratase 1 | c1745 | 1579788 | 315 | N |
| ***adhE*** | fused acetaldehyde-CoA dehydrogenase and iron-dependent alcohol dehydrogenase and pyruvate-forn | c1705 | 1542065 | 19.25 | Y |
| ***adhP*** | alcohol dehydrogenase, 1-propanol preferring | c1911 | 1746856 | 50.15 | N |
| ***adk*** | adenylate kinase | c0594 | 571836 | 143 | N |
| ***alpA*** | Prophage CP4-57 regulatory protein alpA | c1169 | 1131856 | 1845 | Y |
| ***amiA*** | N-acetylmuramoyl-l-alanine amidase I | c2969 | 2829830 | 119 | N |
| ***ansB*** | periplasmic L-asparaginase II | c3543 | 3396623 | 27 | N |
| ***araF*** | periplasmic-binding component of an ABC superfamily L-arabinose transporter | c2314 | 2134854 | 36.3 | N |
| ***arnA*** | bifunctional UDP-L-Ara4N formyltransferase/UDP-GlcA C-4'-decarboxylase | c2797 | 2655988 | 36.4 | N |
| ***bcsE*** | conserved protein | c4348 | 4135675 | 24.45 | N |
| ***bfd*** | bacterioferritin-associated ferredoxin | c4108 | 3898419 | 120.5 | N |
| ***chuA*** | outer membrane heme/hemoglobin receptor | c4308 | 4088366 | 3245 | Y |
| ***chuT*** | putative periplasmic binding protein | c4313 | 4088708 | 2315 | Y |
| ***cirA*** | outer membrane transporter for monomeric catechol-containing ferric ion-siderophore complexes | c2690 | 2538669 | 3050 | N |
| ***clpP*** | proteolytic subunit of ClpA-ClpP and ClpX-ClpP ATP-dependent serine proteases | c0553 | 530696 | 27.95 | N |
| ***csrB*** | regulatory sRNA | c5674 | 3199204 | 34 | N |
| ***cydA*** | cytochrome d terminal oxidase, subunit I | c0811 | 793227 | 20.25 | N |
| ***dapA*** | dihydrodipicolinate synthase | c3006 | 2870418 | 17 | N |
| ***dhaM*** | dihydroxyacetone kinase phosphoryl donor subunit | c1656 | 1491491 | 20.45 | Y |
| ***div*** | predicted flagella assembly protein | c2866 | 2720156 | 74.65 | N |
| ***dmsA*** | dimethyl sulfoxide reductase, anaerobic, subunit A | c1031 | 997107 | 15.15 | N |
| ***dps*** | Fe-binding and storage protein | c0898 | 871437 | 26.65 | N |
| ***ecnB*** | entericidin B membrane lipoprotein | c5235 | 4895400 | 18.4 | N |
| ***efeU*** | ferrous iron permease | c5651 | 1114470 | 1620 | N |
| ***emrD*** | multidrug efflux system protein | c4597 | 4359496 | 35.95 | N |
| ***emrK*** | EmrKY-TolC multidrug resistance efflux pump, membrane fusion protein component | c2904 | 2765770 | 18.85 | N |
| ***endA*** | DNAase I | c3531 | 3386400 | 15.2 | Y |
| ***entC*** | isochorismate synthase 1 | c0680 | 666892 | 2860 | N |
| ***entS*** | enterobactin exporter | c0678 | 664482 | 3485 | N |
| ***eptA*** | predicted metal dependent hydrolase | c5119 | 4892546 | 16.1 | N |
| ***evgA*** | DNA-binding response regulator in two-component regulatory system with EvgS | c2905 | 2765770 | 18.85 | N |
| ***exbB*** | membrane spanning protein in TonB-ExbB-ExbD complex | c3741 | 3573722 | 676 | N |
| ***fadD*** | acyl-CoA synthetase, long-chain-fatty-acid--CoA ligase | c2209 | 2040306 | 30.25 | N |
| ***feoA*** | ferrous iron transporter, protein A | c4185 | 3966911 | 751 | N |
| ***fepA*** | iron-enterobactin outer membrane transporter | c0669 | 654906 | 3285 | N |
| ***fepB*** | periplasmic-binding component of an ABC superfamily iron-enterobactin transporter | c0679 | 666892 | 2860 | N |
| ***fepD*** | Ferric enterobactin transport system permease protein fepD | c0677 | 664482 | 3485 | N |
| ***fes*** | enterobactin/ferric enterobactin esterase | c0671 | 654906 | 3285 | N |
| ***fhuA*** | ferrichrome outer membrane transporter | c0185 | 177917 | 1530 | N |
| ***fhuE*** | ferric-rhodotorulic acid outer membrane transporter | c1374 | 1305180 | 3310 | N |
| ***fhuF*** | ferric iron reductase involved in ferric hydroximate transport | c5446 | 5194712 | 5300 | N |
| ***fimB*** | tyrosine recombinase/inversion of on/off regulator of fimA | c5391 | 5134891 | 16 | N |
| ***fiu*** | predicted iron outer membrane transporter | c0890 | 864152 | 1765 | N |
| ***fliE*** | flagellar basal-body component | c2353 | 2164945 | 34.5 | N |
| ***fliF*** | flagellar basal-body MS-ring and collar protein | c2354 | 2164945 | 34.5 | N |
| ***flu*** | Ag43b, antigen 43 phase-variable biofilm formation autotransporter | c3655 | 3493929 | 26.85 | Y |
| ***fmlA*** | major subunit of F9 fimbriae | c1936 | 1778955 | 79.65 | Y |
| ***fmlB*** | F9 fimbriae chaperone | c1935 | 1777552 | 52.1 | Y |

| | | | | | |
|---|---|---|---|---|---|
| ***frr*** | ribosome recycling factor | c0208 | 202883 | 20.05 | N |
| ***ftn*** | ferritin iron storage protein (cytoplasmic) | c2321 | 2137279 | 1065 | N |
| ***fyuA*** | Putative pesticin receptor precursor | c2436 | 2247059 | 48.9 | Y |
| ***galK*** | galactokinase | c0833 | 811412 | 16.6 | N |
| ***garD*** | D-galactarate dehydratase | c3883 | 3712337 | 56.35 | N |
| ***garP*** | predicted (D)-galactarate transporter | c3882 | 3712337 | 56.35 | N |
| ***gcvR*** | DNA-binding transcriptional repressor, regulatory protein accessory to GcvA | c3007 | 2870418 | 17 | N |
| ***glcG*** | conserved protein | c3706 | 3538872 | 22.15 | Y |
| ***gltA*** | citrate synthase | c0796 | 781330 | 15.3 | N |
| ***gltB*** | glutamate synthase, large subunit | c3973 | 3751590 | 35.5 | N |
| ***glyA*** | serine hydroxymethyltransferase | c3073 | 2987843 | 18.45 | N |
| ***gmr*** | modulator of Rnase II stability | c1756 | 1589434 | 18.5 | N |
| ***gpmA*** | phosphoglyceromutase 1 | c0831 | 803233 | 677 | N |
| ***gpt*** | guanine-xanthine phosphoribosyltransferase | c0384 | 363950 | 16 | N |
| ***greB*** | transcription elongation factor | c4183 | 3963648 | 16.15 | N |
| ***grxB*** | glutaredoxin 2 (Grx2) | c1331 | 1268000 | 70 | N |
| ***gsp*** | glutathionylspermidine synthetase/amidase | c3725 | 3560129 | 16.2 | Y |
| ***gspC*** | general secretory pathway component, cryptic | c4095 | 3887074 | 28.7 | Y |
| ***hemA*** | glutamyl tRNA reductase | c1668 | 1503638 | 22.5 | Y |
| ***hinT*** | purine nucleoside phosphoramidase | c1376 | 1305180 | 3310 | N |
| ***hipA*** | regulator with hipB | c1940 | 1782581 | 17.9 | N |
| ***hma*** | haem receptor | c2482 | 2322265 | 1383 | Y |
| ***hybO*** | hydrogenase 2, small subunit | c3734 | 3568364 | 369 | N |
| ***idnK*** | D-gluconate kinase, thermosensitive | c5369 | 5114115 | 16 | Y |
| ***iha*** | catecholate siderophore receptor/adhesion protein | c3610 | 3450905 | 649 | Y |
| ***intT*** | integrase for prophage | c0932 | 910468 | 19.25 | Y |
| ***ipuB*** | Type 1 fimbriae regulatory protein fimB | c2898 | 2758740 | 24.3 | N |
| ***iroB*** | putative glucosyltransferase | c1254 | 1208113 | 1125 | Y |
| ***iroN*** | siderophore receptor IroN | c1250 | 1198978 | 2115 | Y |
| ***irp2_1*** | N-terminal fragment of yersiniabactin synthase irp2 pseudogene | c2424 | 2227070 | 2150 | Y |
| ***iucA*** | aerobactin synthetase complex subunit | c3627 | 3469166 | 341 | Y |
| ***ivbL*** | ilvB operon leader peptide | c5498 | 4359496 | 35.95 | N |
| ***kdgT*** | 2-keto-3-deoxy-D-gluconate transporter | c4861 | 4621116 | 35 | N |
| ***kefA*** | mechanosensitive channel protein | c0584 | 562094 | 16.15 | N |
| ***kpsM*** | transmembrane component of polysialic acid transporter | c3698 | 3529799 | 59 | Y |
| ***ksl(k2)C*** | predicted glycosyl transferase | c3694 | 3525001 | 23.25 | Y |
| ***ksl(k2)E*** | Hypothetical protein | c3692 | 3521018 | 16 | Y |
| ***lolB*** | chaperone for lipoproteins | c1667 | 1503638 | 22.5 | N |
| ***map*** | methionine aminopeptidase | c0203 | 199905 | 78.4 | N |
| ***mchB*** | mchB protein | c1227 | 1176371 | 625 | Y |
| ***mchF*** | Probable microcin H47 secretion ATP-binding protein | c1232 | 1182622 | 18 | Y |
| ***metB*** | cystathionine gamma-synthase, PLP-dependent | c4892 | 4648097 | 14.7 | N |
| ***metC*** | cystathionine beta-lyase, PLP-dependent | c3742 | 3573722 | 676 | N |
| ***metJ*** | DNA-binding transcriptional repressor, S-adenosylmethionine-binding | c4891 | 4648097 | 14.7 | N |
| ***metK*** | methionine adenosyltransferase 1 | c3528 | 3382444 | 15.45 | N |
| ***mglC*** | membrane component of an ABC superfamily methyl-galactoside transporter | c2682 | 2529416 | 15.1 | N |
| ***mltD*** | membrane-bound lytic murein transglycosylase D | c0248 | 244880 | 17.8 | Y |
| ***mntH*** | proton-dependent divalent metal cation transporter | c2931 | 2795193 | 618.5 | N |
| ***modB*** | molybdate transporter subunit; membrane component of ABC superfamily | c0841 | 818028 | 29.6 | N |
| ***mutL*** | methyl-directed mismatch repair protein | c5254 | 5005732 | 25.85 | N |
| ***nanR*** | DNA-binding transcriptional dual regulator | c3980 | 3799316 | 20.4 | N |
| ***narQ*** | sensory histidine kinase in two-component regulatory system with NarP (NarL) | c2996 | 2857926 | 52.8 | N |

| | | | | | |
|---|---|---|---|---|---|
| ***narU*** | nitrate/nitrite transporter | c1901 | 1738828 | 125 | N |
| ***nei*** | endonuclease VIII | c0793 | 777655 | 34.3 | N |
| ***nfsA*** | nitroreductase A, NADPH-dependent, FMN-dependent | c0984 | 946101 | 34.15 | N |
| ***nohA*** | Prophage Qin DNA packaging protein NU1 homolog | c1568 | 1424721 | 283 | Y |
| ***nohB*** | Prophage QSR' DNA packaging protein NU1 homolog | c3174 | 3046729 | 20.55 | Y |
| ***norV_2*** | C-terminal fragment of flavorubredoxin oxidoreductase (pseudogene) | c3266 | 3116439 | 23.7 | N |
| ***norW*** | NADH:flavorubredoxin oxidoreductase | c3267 | 3116439 | 23.7 | Y |
| ***nrdH*** | glutaredoxin-like protein | c3226 | 3081278 | 849 | Y |
| ***nth*** | DNA glycosylase and apyrimidinic (AP) lyase (endonuclease III) | c2025 | 1872101 | 56.25 | N |
| ***nupC*** | nucleoside (except guanosine) transporter | c2932 | 2795193 | 618.5 | N |
| ***ompR*** | DNA-binding response regulator in two-component regulatory system with EnvZ | c4181 | 3963648 | 16.15 | N |
| ***oppA*** | periplasmic-binding component of an ABC superfamily oligopeptide transporter | c1707 | 1544854 | 17.45 | N |
| ***oxyR*** | DNA-binding transcriptional dual regulator | c4922 | 4688193 | 20 | N |
| ***oxyS*** | OxyS RNA | c5628 | 4688193 | 20 | N |
| ***panD*** | aspartate 1-decarboxylase | c0160 | 156719 | 34.85 | N |
| ***papG_2*** | papG protein | c5179 | 4940337 | 43 | Y |
| ***pepD*** | aminoacyl-histidine dipeptidase (peptidase D) | c0383 | 363950 | 16 | N |
| ***pgaB*** | putatative polysaccharide N-deacetylase/carbohydrate esterase | c1162 | 1122787 | 152 | N |
| ***pgtC*** | regulatory protein | c5202 | 4955438 | 38.5 | Y |
| ***pgtP*** | transporter protein | c5201 | 4955438 | 38.5 | Y |
| ***pheA*** | P-protein | c3120 | 3000348 | 15.2 | N |
| ***phnA*** | conserved protein | c5113 | 4885516 | 25.3 | N |
| ***ppc*** | phosphoenolpyruvate carboxylase | c4915 | 4677686 | 37.15 | N |
| ***priC*** | primosomal replication protein N'' | c0586 | 565475 | 346 | N |
| ***prlC*** | oligopeptidase A | c4297 | 4079238 | 32.75 | N |
| ***pstB*** | phosphate transporter subunit; ATP-binding component of ABC superfamily | c4649 | 4409130 | 21.15 | N |
| ***ptrB_1*** | N-terminal fragment of protease II (pseudogene) | c2256 | 2080440 | 20.9 | N |
| ***ptrB_2*** | C-terminal fragment of protease II (pseudogene) | c2255 | 2080440 | 20.9 | N |
| ***purH*** | bifunctional phosphoribosylaminoimidazolecarboxamide formyltransferase/IMP cyclohydrolase | c4964 | 4738854 | 24.1 | N |
| ***purU*** | formyltetrahydrofolate hydrolase | c1696 | 1533622 | 18.75 | N |
| ***recN*** | recombination and repair protein | c3138 | 3014435 | 79.45 | N |
| ***rffE*** | UDP-N-acetyl glucosamine-2-epimerase | c4706 | 4471358 | 14.45 | N |
| ***rhaT*** | L-rhamnose:proton symporter | c4857 | 4620366 | 1535 | N |
| ***rpiB*** | ribose 5-phosphate isomerase B/allose 6-phosphate isomerase | c5096 | 4872502 | 15.3 | N |
| ***rpiR*** | DNA-binding transcriptional repressor | c5095 | 4872502 | 15.3 | N |
| ***rpmB*** | 50S ribosomal subunit protein L28 | c4461 | 4242556 | 15.75 | Y |
| ***rpoC*** | RNA polymerase, beta prime subunit | c4945 | 4717381 | 16.55 | N |
| ***rrlB*** | 23S rRNA (rrlB) | c5610 | 4700985 | 27.6 | N |
| ***rrsE*** | 16S rRNA (rrnE) | c5612 | 4738854 | 24.1 | N |
| ***rspB*** | predicted oxidoreductase, Zn-dependent and NAD(P)-binding | c1970 | 1814635 | 53.5 | N |
| ***rtcB*** | conserved protein | c4198 | 3984567 | 17.8 | N |
| ***rtcR*** | sigma 54-dependent transcriptional regulator of rtcBA expression | c4199 | 3984567 | 17.8 | N |
| ***ryhB*** | regulatory antisense RNA | c5652 | 4020332 | 4670 | N |
| ***sdaC*** | predicted serine transporter | c3364 | 3203013 | 15.05 | N |
| ***sdhA*** | succinate dehydrogenase, flavoprotein subunit | c0801 | 783328 | 14.7 | N |
| ***sdhC*** | succinate dehydrogenase, membrane subunit, binds cytochrome b556 | c0798 | 781330 | 15.3 | N |
| ***sdiA*** | DNA-binding transcriptional activator | c2330 | 2145538 | 466 | N |
| ***secA*** | preprotein translocase subunit, ATPase | c0116 | 114528 | 109.5 | N |
| ***selA*** | selenocysteine synthase | c4412 | 4196329 | 48.25 | Y |
| ***sfaB*** | Putative F1C and S fimbrial switch regulatory protein | c1238 | 1187346 | 57.4 | Y |
| ***sfaC*** | putative F1C and S fimbrial switch regulatory protein | c1237 | 1187346 | 57.4 | Y |
| ***sitA*** | iron transport protein, periplasmic-binding protein (pseudogene) | c1600 | 1450244 | 526 | Y |

| | | | | | |
|---|---|---|---|---|---|
| ***sitC*** | sitC protein | c1598 | 1448005 | 19.8 | Y |
| ***sodA*** | superoxide dismutase, Mn | c4859 | 4620366 | 1535 | N |
| ***spoT*** | bifunctional (p)ppGpp synthetase II and guanosine-3',5'-bis pyrophosphate 3'-pyrophosphohydrolase | c4475 | 4254322 | 22.95 | N |
| ***sprT*** | conserved protein | c3530 | 3385886 | 19.25 | N |
| ***sucD*** | succinyl-CoA synthetase, NAD(P)-binding, alpha subunit | c0806 | 791400 | 20.05 | N |
| ***sufA*** | Fe-S cluster assembly protein | c2079 | 1920586 | 103.6 | N |
| ***sulA*** | SOS cell division inhibitor | c1095 | 1064889 | 14.85 | N |
| ***sxy*** | CRP-S promoter co-activator | c1096 | 1064889 | 14.85 | N |
| ***symR*** | regulatory sRNA | c5821 | 5165150 | 54.9 | Y |
| ***tauA*** | periplasmic-binding component of an ABC superfamily taurine transporter | c0472 | 460235 | 15.31 | N |
| ***tdcF*** | predicted L-PSP (mRNA) endoribonuclease | c3871 | 3699491 | 29.1 | N |
| ***tff*** | small RNA | c5703 | 199905 | 78.4 | N |
| ***thrS*** | threonyl-tRNA synthetase | c2116 | 1956892 | 20.7 | N |
| ***tldD*** | predicted peptidase | c3999 | 3815606 | 60.1 | N |
| ***tonB*** | TonB protein | c1717 | 1554961 | 153.5 | N |
| ***torD*** | chaperone involved in maturation of TorA subunit of trimethylamine N-oxide reductase system I | c1134 | 1096663 | 16.95 | N |
| ***treB*** | PTS system trehalose-specific EIIBC component | c5339 | 5082191 | 46.1 | N |
| ***trkA*** | NAD-binding component of TrK potassium transporter | c4050 | 3857900 | 17.25 | N |
| ***trpC*** | indole-3-glycerol-phosphate synthase/phosphoribosylanthranilate isomerase | c5733 | 1563103 | 23.54 | N |
| ***ttdA*** | L-tartrate dehydratase, alpha subunit | c3812 | 3647948 | 31.1 | N |
| ***tus*** | inhibitor of replication at Ter, DNA-binding protein | c2002 | 1845253 | 36.25 | N |
| ***uvrB*** | excinulease of nucleotide excision repair, DNA damage recognition component | c0860 | 837213 | 34.55 | N |
| ***waaW*** | UDP-galactose:(galactosyl) LPS alpha1,2-galactosyltransferase | c4450 | 4232472 | 30.3 | Y |
| ***wzy*** | O6 antigen polymerase | c2564 | 2399829 | 15.8 | Y |
| ***xylG*** | fused D-xylose transporter subunits of ABC superfamily: ATP-binding components | c4387 | | 19.5 | N |
| ***yadD*** | predicted transposase | c0162 | 156719 | 34.85 | N |
| ***yagU*** | conserved inner membrane protein | c0399 | 377918 | 31.6 | Y |
| ***yahM*** | predicted protein (pseudogene) | c5710 | 434828 | 48.3 | N |
| ***yahN*** | neutral amino-acid efflux system | c0448 | 436090 | 18.25 | N |
| ***yahO*** | predicted protein | c0449 | 436090 | 18.25 | N |
| ***yaiS*** | conserved protein | c0470 | 460235 | 15.31 | N |
| ***yaiW*** | predicted DNA-binding transcriptional regulator | c0483 | 472851 | 20.95 | N |
| ***ybaN*** | conserved inner membrane protein | c0587 | 565475 | 346 | N |
| ***ybtA*** | Putataive AraC type regulator | c2423 | 2225888 | 1445 | Y |
| ***ybtP*** | Putative inner membrane ABC-transporter | c2422 | 2225888 | 1445 | Y |
| ***ycbJ*** | conserved protein | c1060 | 1022622 | 27.1 | N |
| ***ycbZ*** | predicted peptidase | c1091 | 1061458 | 15.4 | N |
| ***yceJ*** | predicted cytochrome b561 | c1323 | 1262613 | 297 | N |
| ***ycfQ*** | predicted DNA-binding transcriptional regulator | c1385 | 1313122 | 14.75 | Y |
| ***ycfR*** | predicted protein | c1386 | 1313122 | 14.75 | Y |
| ***ycfZ*** | predicted inner membrane protein | c1396 | 1325655 | 24.25 | N |
| ***ycgF*** | predicted FAD-binding phosphodiesterase | c1606 | 1455262 | 16.2 | Y |
| ***ycgZ*** | predicted protein | c1607 | 1455262 | 16.2 | Y |
| ***yciE*** | conserved protein | c1723 | 1558968 | 16.55 | N |
| ***yciF*** | conserved protein | c1724 | 1559726 | 163 | N |
| ***yciI*** | predicted enzyme | c1716 | 1554961 | 153.5 | N |
| ***ydbA*** | predicted autotransporter (pseudogene) | c5683 | 1669237 | 21.7 | N |
| ***ydbA_2*** | C-terminal fragment of a predicted autotransporter (pseudogene) | c1831 | 1669237 | 21.7 | N |
| ***ydcX*** | predicted inner membrane protein | c1870 | 1707547 | 119 | Y |
| ***yddA*** | membrane and ATP-binding components of an ABC superfamily predicted multidrug transporter | c1925 | 1765013 | 657 | N |
| ***ydeU*** | C-terminal fragment of an outer membrane autotransporter (pseudogene) | c2894 | 2748488 | 22.85 | N |
| ***ydhS*** | conserved protein with FAD/NAD(P)-binding domain | c2060 | 1903261 | 20.9 | N |

| | | | | | |
|---|---|---|---|---|---|
| ***ydhX*** | predicted 4Fe-4S ferridoxin-type protein | c2065 | 1907103 | 15.3 | N |
| ***ydhY*** | predicted 4Fe-4S ferridoxin-type protein | c2068 | 1910890 | 54.65 | N |
| ***ydiE*** | conserved protein | c2101 | 1945533 | 1080 | N |
| ***ydiV*** | conserved protein | c2103 | 1947970 | 284 | N |
| ***yebS*** | conserved inner membrane protein | c2242 | 2068061 | 98.5 | N |
| ***yecI*** | predicted ferritin-like protein | c2315 | 2135624 | 168 | N |
| ***yegP*** | predicted protein | c2606 | 2451787 | 52 | Y |
| ***yegU*** | predicted hydrolase | c2626 | 2470773 | 115 | N |
| ***yehB*** | predicted outer membrane protein | c2636 | 2480851 | 16.9 | N |
| ***yehH*** | molybdate metabolism regulator | c2643 | 2487858 | 57.25 | N |
| ***yeiT*** | predicted oxidoreductase | c2679 | 2525580 | 64.5 | N |
| ***yfeC*** | predicted DNA-binding transcriptional regulator | c2934 | 2799784 | 16 | N |
| ***yffB*** | conserved protein | c2998 | 28961945 | 50.15 | N |
| ***yfgF*** | predicted inner membrane protein | c3021 | 2885608 | 46.9 | N |
| ***yfhK*** | predicted sensory kinase in two-component system | c3079 | 2953695 | 29.05 | N |
| ***ygaP*** | predicted inner membrane protein with hydrolase activity | c3217 | 3076232 | 35.9 | Y |
| ***ygcF*** | conserved protein | c3335 | 3177509 | 32 | Y |
| ***ygdG*** | Ssb-binding protein | c3366 | 3206632 | 23.85 | N |
| ***ygeA*** | predicted racemase | c3437 | 3288381 | 20.5 | N |
| ***ygfK*** | predicted oxidoreductase, Fe-S subunit | c3456 | 3312136 | 312.55 | N |
| ***ygfQ*** | predicted transporter | c3462 | 3323876 | 45.45 | N |
| ***yghU*** | predicted glutathionylspermidine-utilizing glutathione transferase | c3726 | 3560129 | 16.2 | N |
| ***ygjK*** | predicted glycosyl hydrolase | c3838 | 3669455 | 15 | N |
| ***yhaO*** | predicted transporter (pseudogene) | c5795 | 3696319 | 14.4 | N |
| ***yhaO_1*** | N-terminal fragment of a predicted transporter (pseudogene) | c3869 | 3696319 | 14.4 | N |
| ***yhaO_2*** | C-terminal fragment of a predicted transporter (pseudogene) | c3868 | 3696319 | 14.4 | N |
| ***yhiR*** | predicted DNA (exogenous) processing protein | c4298 | 4079238 | 32.75 | N |
| ***yhjV*** | predicted transporter | c4354 | 4140226 | 48.9 | N |
| ***yibD*** | predicted glycosyl transferase | c4441 | 4224165 | 22.25 | Y |
| ***yieK*** | predicted 6-phosphogluconolactonase | c4640 | 4400271 | 22.2 | N |
| ***yjbJ*** | predicted stress response protein | c5016 | 4791242 | 20 | N |
| ***yjdA*** | conserved protein with nucleoside triphosphate hydrolase domain | c5114 | 4885516 | 25.3 | N |
| ***yjdQ*** | phage integrase (pseudogene) | c5817 | 4919154 | 18.8 | Y |
| ***yjfL_1*** | N-terminal fragment of a conserved inner membrane protein (pseudogene) | c5268 | 5020380 | 19.6 | N |
| ***yjfL_2*** | C-terminal fragment of a conserved inner membrane protein (pseudogene) | c5269 | 5020380 | 19.6 | N |
| ***yjhA*** | N-acetylnuraminic acid outer membrane channel protein | c5389 | 5134891 | 16 | N |
| ***yjhS*** | conserved protein | c5387 | 5131678 | 14.85 | Y |
| ***yjiW*** | conserved protein | c5422 | 5165150 | 54.9 | Y |
| ***yjjZ*** | predicted protein | c5447 | 5194712 | 5300 | N |
| ***ykgG*** | predicted transporter | c0424 | 408627 | 17.95 | Y |
| ***ykgH*** | predicted inner membrane protein | c0425 | 409825 | 19.9 | Y |
| ***yliF*** | predicted diguanylate cyclase | c0919 | 896568 | 20.7 | N |
| ***ymgI*** | Hypothetical protein | c1618 | 1461122 | 45.55 | Y |
| ***yncD_1*** | N-terminal fragment of a predicted TonB-dependent receptor (pseudogene) | c1876 | 1711868 | 845 | Y |
| ***yncE*** | conserved protein | c1877 | 1711868 | 845 | Y |
| ***yncL*** | conserved protein | c5738 | 1707547 | 119 | Y |
| ***ynfC*** | predicted protein | c1975 | 1818174 | 28.25 | N |
| ***ynfD*** | predicted protein | c1976 | 1818174 | 28.25 | N |
| ***ynfE*** | oxidoreductase subunit | c1977 | 1818694 | 17.4 | N |
| ***yoaD*** | predicted phosphodiesterase | c2221 | 2051222 | 45.8 | Y |
| ***yobD*** | conserved inner membrane protein | c2227 | 2056241 | 489.5 | N |
| ***yoeH*** | predicted transposase (pseudogene) | c5760 | 2322265 | 1383 | Y |

| | | | | | |
|---|---|---|---|---|---|
| ***yojL*** | predicted thiamine biosynthesis lipoprotein | c2756 | 2595634 | 1990 | N |
| ***ypeA*** | predicted acyltransferase with acyl-CoA N-acyltransferase domain | c2968 | 2829830 | 119 | N |
| ***ypfM*** | conserved protein | c5776 | 28961945 | 50.15 | N |
| ***yphC*** | predicted oxidoreductase, Zn-dependent and NAD(P)-binding | c3067 | 2938778 | 23.1 | N |
| ***yqiH*** | Hypothetical fimbrial chaperone yqiH precursor | c3793 | 3627804 | 24.7 | Y |
| ***yqjH*** | predicted siderophore interacting protein | c3823 | 3657157 | 1845 | N |
| ***yqjI*** | predicted transcriptional regulator | c3824 | 3657157 | 1845 | N |
| ***yrbL*** | predicted protein | c3967 | 3781815 | 72.25 | N |
| ***yrfF*** | predicted inner membrane protein | c4169 | 3953727 | 18.1 | N |
| ***yrhB*** | predicted protein | c4235 | 4023214 | 44.5 | N |
| ***ytfB*** | predicted cell envelope opacity-associated protein | c5305 | 5045455 | 25 | Y |
| **c3775** | putative iron compound receptor | c3775 | 3607979 | 2720 | Y |
| **c3774** | Ferric enterobactin transport ATP-binding protein fepC | c3774 | 3607777 | 2545 | Y |
| **c1249** | Hypothetical protein | c1249 | 1198978 | 2115 | Y |
| **c5174** | Putative iron-regulated outer membrane virulence protein | c5174 | 4935947 | 1485 | Y |
| **c5081** | Putative conserved protein | c5081 | 4856192 | 1200 | Y |
| **c1255** | Hypothetical protein | c1255 | 1208113 | 1125 | Y |
| **c1226** | Hypothetical protein | c1226 | 1176371 | 625 | Y |
| **c1601** | Hypothetical protein | c1601 | 1450244 | 526 | Y |
| **c0327** | hypothetical protein | c0327 | 299222 | 463.5 | Y |
| **c1234** | hypothetical protein | c1234 | 1184948 | 382 | Y |
| **c1235** | hypothetical protein | c1235 | 1184948 | 382 | Y |
| **c1485** | Hypothetical protein | c1485 | 1379394 | 80.3 | Y |
| **c1219** | internal fragment of an ISL3 family transposase (pseudogene) | c1219 | 1172463 | 76.7 | Y |
| **c1220** | Phospho-2-dehydro-3-deoxyheptonate aldolase, Trp-sensitive | c1220 | 1172463 | 76.7 | Y |
| **c0023** | Hypothetical protein | c0023 | 19193 | 65.1 | Y |
| **c1910** | Hypothetical protein | c1910 | 1745699 | 52.2 | Y |
| **c0281** | Hypothetical protein | c0281 | 262978 | 50.1 | Y |
| **c5516** | tRNA-OTHER | c5516 | 434828 | 48.3 | Y |
| **c2406** | Hypothetical protein | c2406 | 2210089 | 46.9 | Y |
| **c2407** | Hypothetical protein | c2407 | 2210089 | 46.9 | Y |
| **c0352** | partial transposase | c0352 | 331734 | 43.95 | Y |
| **c0294** | Hypothetical protein | c0294 | 273943 | 39.95 | Y |
| **c2518** | TonB dependent receptor | c2518 | 2357547 | 39.1 | Y |
| **c2470** | N-terminal fragment of a putative polyketide synthase (pseudogene) | c2470 | 2314838 | 31.7 | Y |
| **c3652** | Hypothetical protein yfjI | c3652 | 3489365 | 31.22 | Y |
| **c5160** | Hypothetical transcriptional regulator yfjR | c5160 | 4928531 | 27.35 | Y |
| **c2412** | Hypothetical protein | c2412 | 2214216 | 23.55 | Y |
| **c0346** | N-terminal fragment of a hypothetical protein (pseudogene) | c0346 | 322768 | 20.15 | Y |
| **c1208** | Hypothetical protein | c1208 | 1165274 | 18.3 | Y |
| **c5384** | Hypothetical protein | c5384 | 5129997 | 17.8 | Y |
| **c4279** | PTS system, galactitol-specific IIC component (EIIC-GAT) | c4279 | 4059407 | 17.1 | Y |
| **c3390** | Hypothetical protein | c3390 | 3228862 | 16.55 | Y |
| **c3557** | shiA homolog | c3557 | 3407865 | 15.5 | Y |
| **c5209** | Hypothetical protein ybdN | c5209 | 4964081 | 15.1 | Y |
| **c1410** | Hypothetical protein | c1410 | 1334250 | 14.9 | Y |
| **c1411** | unknown protein encoded by cryptic prophage | c1411 | 1334250 | 14.9 | Y |
| **c4492** | ShiA homolg | c4492 | 4276206 | 14.85 | Y |
| **c1470** | Hypothetical protein | c1470 | 1340976 | 14.7 | Y |
| **c1442** | unknown protein encoded within prophage | c1442 | 1348480 | 14.69 | Y |
| **c1428** | Hypothetical protein | c1428 | 1342714 | 14.35 | Y |
| **c1166** | hypothetical protein | c1166 | 1129235 | 207 | Y |

| | | | | | |
|---|---|---|---|---|---|
| **c4496** | N-terminal fragment of a predicted glycoside hydrolase (pseudogene) | c4496 | 4281546 | 60.1 | Y |
| **c5213** | putative transposase for IS629 | c5213 | 4966472 | 46.1 | Y |
| **c0139** | putative transposase for IS629 | c0139 | 131943 | 44.5 | Y |
| **c1553** | putative transposase for IS629 | c1553 | 1416771 | 43.9 | Y |
| **c1262** | putative transposase for IS629 | c1262 | 1212816 | 43.7 | Y |
| **c1521** | putative transposase for IS629 | c1521 | 1399561 | 43.15 | Y |
| **c5178** | putative transposase for IS629 | c5178 | 4940337 | 43 | Y |
| **c5167** | putative transposase for IS629 | c5167 | 4932242 | 42.5 | Y |
| **c0293** | Hypothetical protein | c0293 | 272723 | 41.75 | Y |
| **c4497** | C-terminal fragment of a predicted glycoside hydrolase (pseudogene) | c4497 | 4282106 | 39.95 | Y |
| **c5035** | Putative 2-oxoglutarate dehydrogenase | c5035 | 4812698 | 35.1 | Y |
| **c0411** | putative LysR-like transcriptional regulator | c0411 | 391969 | 32.8 | Y |
| **c2562** | Glycosyl transferase | c2562 | 2395928 | 31.9 | Y |
| **c2471** | Hypothetical protein | c2471 | 2314838 | 31.7 | Y |
| **c3203** | Hypothetical protein | c3203 | 3063088 | 26 | Y |
| **c3750** | Putative regulator | c3750 | 3583612 | 25.85 | Y |
| **c1259** | internal fragment of a putative inner membrane protein (pseudogene) | c1259 | 1210832 | 24.8 | Y |
| **c1439** | Hypothetical protein | c1439 | 1346824 | 24 | Y |
| **c3636** | Hypothetical protein | c3636 | 3475848 | 22.4 | Y |
| **c3713** | Hypothetical protein | c3713 | 3547658 | 22 | Y |
| **c4213** | Chaperone protein fimC precursor | c4213 | 3998134 | 22 | Y |
| **c1197** | putative enzyme | c1197 | 1153839 | 21.1 | Y |
| **c4516** | Hypothetical protein | c4516 | 4302546 | 20.45 | Y |
| **c0319** | putative oligogalacturonide lyase | c0319 | 290291 | 20.15 | Y |
| **c5295** | Hypothetical protein | c5295 | 5035359 | 19.5 | Y |
| **c3176** | GnsB protein | c3176 | 3048166 | 19.15 | Y |
| **c4521** | hypothetical protein | c4521 | 4304852 | 18.15 | Y |
| **c0363** | Putative RTX family exoprotein A gene | c0363 | 344112 | 17.35 | Y |
| **c2467** | Putative 3-hydroxyacyl-CoA dehydrogenase | c2467 | 2301530 | 16.5 | Y |
| **c5164** | Hypothetical protein | c5164 | 4931346 | 16.45 | Y |
| **c3634** | Hypothetical protein yjhT precursor | c3634 | 3474111 | 16.25 | Y |
| **c4541** | Putative maturase-related protein | c4541 | 4321344 | 16.1 | Y |
| **c3029** | RatA homolog | c3029 | 2896596 | 16 | Y |
| **c0757** | Hypothetical protein | c0757 | 738754 | 15.6 | Y |
| **c4545** | Hypothetical protein | c4545 | 4322289 | 15.25 | Y |
| **c4895** | Hypothetical protein | c4895 | 4654018 | 14.95 | Y |
| **c5424** | putative restriction modification enzyme M subunit (methylase) | c5424 | 5167741 | 14.5 | Y |
| **c4925** | putative citrate permease | c4925 | 4692929 | 14.4 | Y |
| **c3597** | transposase | c3597 | 3440893 | 3620 | Y |
| **c4561** | Hypothetical protein | c4561 | 4333067 | 1730 | Y |
| **c2409** | Hypothetical protein | c2409 | 2211750 | 15.6 | Y |

[1]CFT073 genes that have a Fur binding site as determined by ChIP-seq.

[2]Gene functions, predicted operons, gene names, protein annotations and c numbers are obtained from Ecocyc.

[3]The genomic location and maximum amplitude of Fur binding in the CFT073 genome (NC_004431.1)
as obtained from ChIP-seq and visualized by MochiView genome browser.

[4](Y) or (N) indicates whether the Fur ChIP-seq peak is located in a pathogenicity island.

Table S5B. Fur binding motifs for Chip-seq peaks for CFT073 specific genes

| Gene[1] | c-number | Region searched[2] | Location of the motif[3] | | Matched Sequence[4] | q-value |
|---|---|---|---|---|---|---|
| | | | Start | Stop | | |
| *iucA* | c3627 | NC_004431.1:3468986-3469337 | 200 | 218 | GATAATGAGAATCATTATT | 3.66E-05 |
| *shiF* | c3628 | NC_004431.1:3469051-3469290 | 135 | 153 | GATAATGAGAATCATTATT | 3.22E-05 |
| *ybtP* | c2422 | NC_004431.1:2225751-2225940 | 138 | 156 | GTGAATAATAACCATTATC | 0.00104 |
| *sitA* | c1600 | NC_004431.1:1450110-1450381 | 118 | 136 | GCTAATGATAATCATTTTC | 3.66E-05 |
| *irp2_1* | c2424 | NC_004431.1:2226913-2227238 | 153 | 171 | GAGAATAATAATTATTAAC | 0.00088 |
| *iroN* | c1250 | NC_004431.1:1197956-1198177 | 95 | 113 | AAGAATGATAATTAATATC | 0.00088 |
| *iroB* | c1254 | NC_004431.1:1208004-1208237 | 107 | 125 | GCTAATGATAATAATTACC | 0.00015 |
| *ireA* | c5174 | NC_004431.1:4935789-4936098 | 157 | 175 | GCTAACGCAAATCATTATC | 0.00057 |
| *hma* | c2482 | NC_004431.1:2322182-2322387 | 89 | 107 | TGTAATGATAATTATTATC | 0.00018 |
| *chuA* | c4308 | NC_004431.1:4088203-4088532 | 144 | 162 | GATAATGAGAATTATTATC | 3.66E-05 |
| *chuT* | c4313 | NC_004431.1:4088544-4088873 | 154 | 172 | GATAATCATGATCATTCTC | 0.00015 |
| *c1220* | c1220 | NC_004431.1:1172362-1172559 | 98 | 116 | GATAATGATAATCATTTAA | 0.00014 |
| *mchB* | c1227 | NC_004431.1:1176262-1176495 | 97 | 115 | GTAAATAACAATCATTCTC | 0.00445 |
| *ybtA* | c2423 | NC_004431.1:2225733-2226016 | 156 | 174 | GTGAATAATAACCATTATC | 0.00088 |
| *alpA* | c1169 | NC_004431.1:1131813-1132034 | 35 | 53 | GATAATGAGAATCGACAGC | 0.00225 |
| *c1255* | c1255 | NC_004431.1:1208050-1208247 | 61 | 79 | GCTAATGATAATAATTACC | 0.00016 |
| *c2412* | c2412 | NC_004431.1:2214064-2214417 | 144 | 162 | GGAATTGATAATCATTATT | 0.00088 |

[1]Gene name and c numbers were taken from EcoCyc

[2]The location of the Fur ChIP-seq peak in 5' upstream position of the respective gene from the MochiView genome browser used for searching for the Fur consensus motif "GATAATGATAATCATTATC" using FIMO tool in MEME search engine.

[3]Location of the matched motif within the search region.

[4]Sequence of the matched motif
