# Supplementary material for: Tailoring a Global Iron Regulon to a Uropathogen
Source: mBio. 2020 Mar 24;11(2):e00351-20. doi: 10.1128/mBio.00351-20 (PMC7157518; doi:10.1128/mBio.00351-20)
Supplement: TABLE S6 [file mBio.00351-20-st006.pdf]

Table S6. Genes differentially expressed in CFT073 *fur* mutant and in strains grown in urine or obtained from a urinary tract infection[1]

Gene differentially expressed during UTI

Gene[2]	Protein product[2]	c-number[2]	Expression during UTI[3]	References[4]
iucA	aerobactin synthetase complex subun	c3627	+	a
iucB	iucB protein	c3626	+	a
iucC	iucC protein	c3625	+	a
iucD	iucD protein	c3624	+	a
iutA	iutA protein	c3623	+	f
fepA	iron-enterobactin outer membrane tr	c0669	+	a,d,f
cirA	outer membrane transporter for mon	c2690	+	f
fyuA	Putative pesticin receptor precursor	c2436	+	f
iroN	siderophore receptor IroN	c1250	+	a
iroB	putative glucosyltransferase	c1254	+	c
entC	isochorismate synthase 1	c0680	+	c
entE	Enterobactin synthetase component E	c0681	+	a,c
entA	2,3-dihydro-2,3-dihydroxybenzoate de	c0683	+	c
entF	enterobactin synthase multienzyme c	c0673	+	c
chuT	putative periplasmic binding protein	c4313	+	c
chuW	putative oxygen independent copropo	c4314	+	c
chuS	putative heme/hemoglobin transport	c4307	+	c
hma	haem receptor	c2482	+	f
ireA	Putative iron-regulated outer membra	c5174	+	c
sitB	sitB protein	c1599	+	c
irp2_1	N-terminal fragment of yersiniabactin	c2424	+	a
yeiA	predicted oxidoreductase	c2680	+	b
yddB	predicted porin protein	c1924	+	b
c3610	putative tail component of prophage	c3610	+	c
ybdZ	conserved protein	c0672	+	c
c1220	Phospho-2-dehydro-3-deoxyheptonate	c1220	+	b,c
papA	papA protein	c3592	+	a
yncE	conserved protein	c1877	+	c
yeaR	conserved protein	c2204	+	c

Gene differentially expressed in urine [6]

Gene[2]	Protein product[2]	c-number[2]	Expression in urine[3]	References[4]
iucD	iucD protein	c3624	+	c
iutA	iutA protein	c3623	+	c,f
tonB	TonB protein	c1717	+	f
fepA	iron-enterobactin outer membrane tr	c0669	+	f
iroB	putative glucosyltransferase	c1254	+	c
iroN	siderophore receptor IroN	c1250	+	f
fyuA	Putative pesticin receptor precursor	c2436	+	f
fhuA	ferrichrome outer membrane transpo	c0185	+	f
fhuE	ferric-rhodotorulic acid outer membra	c1374	+	f
iha	catecholate siderophore receptor/adh	c3610	+	f
chuA	outer membrane heme/hemoglobin r	c4308	+	f
chuW	putative oxygen independent copropo	c4314	+	e,f
chuX	orf; hypothetical protein	c4315	+	e,f
chuS	putative heme/hemoglobin transport	c4307	+	e,f
hma	haem receptor	c2482	+	e,f
sitA	iron transport protein, periplasmic-bi	c1600	+	e,f
ireA	Putative iron-regulated outer membra	c5174	+	f
c3775	putative iron compound receptor	c3775	+	e,f
artJ	Arginine-binding periplasmic protein	c0993	+	c
argC	N-acetyl-gamma-glutamylphosphate	c4917	+	c
argB	acetylglutamate kinase	c4918	+	c
mchB	mchB protein	c1227	+	c
sat	Aecreted auto transpoter toxin	c3619	+	f
sdhB	succinate dehydrogenase, FeS subuni	c0802	-	e
glcB	malate synthase G	c3705	-	e
ilvB	acetolactate synthase I, large subunit	c4596	-	e
fumA	fumarate hydratase (fumarase A), ae	c2004	-	e
yjiY	predicted inner membrane protein	c5429	+	c
yncE	conserved protein	c1877	+	c
yeaR	conserved protein	c2204	+	c
papA	papA protein	c3592	-	f

papA_2	papA protein	c5188	-	f

[1]List of genes from our dataset that were previously shown to be differentially expressed during UTI and /or during growth in urine.

[2]Gene functions, predicted operons, gene names, protein annotations and c numbers are obtained from Ecocyc.

[3]Expression during UTI or during growth in urine: Upregulation (+) and downregulation (-).

[4]References:

a= Bielecki *et al* ., *mBio* , 2014, "*In vivo* mRNA profiling of uropathogenic *Escherichia coli* from diverse phylogroups reveals common and group-specific gene expression profiles".

b= Subhaschandrabose *et al* ., *Plos Pathogens* , 2013, "Genome-wide detection of fitness genes in uropathogenic *Escherichia coli* during systemic Infection".

c= Synder *et al* ., *Infect Immun* , 2004, " Transcriptome of uropathogenic *Escherichia coli* during Urinary Tract Infection".

d= Subhaschandrabose *et al* ., *PNAS* , 2014, Host-specific induction of *Escherichia coli* fitness genes during human urinary tract infection".

e= Hagan *et al* ., *Plos Pathogen* , 2010, "*Escherichia coli* global gene expression in urine from women with Urinary Tract Infection".

f= Mobley, *Pathogens* , 2016, "Measuring *Escherichia coli* Gene Expression during Human Urinary Tract Infections".
